# Effectiveness and Safety of Treatments for Early‐Stage Merkel Cell Carcinoma: A Systematic Review and Meta‐Analysis of Randomized and Non‐Randomized Studies

**DOI:** 10.1002/cam4.70553

**Published:** 2025-01-03

**Authors:** Yves Paul Vincent Mbous, Rowida Mohamed, Usha Sambamoorthi, Murtuza Bharmal, Khalid M. Kamal, Traci LeMasters, Joanna Kolodney, George A. Kelley

**Affiliations:** ^1^ School of Pharmacy, Department of Pharmaceutical Systems and Policy, Robert C. Byrd Health Sciences Center [North] West Virginia University Morgantown West Virginia USA; ^2^ Department of Obstetrics and Gynecology, Biological Sciences Division The University of Chicago Chicago Illinois USA; ^3^ College of Pharmacy, Department of Pharmacotherapy University of North Texas Health Science Center Fort Worth Texas USA; ^4^ AstraZeneca Oncology Outcomes Research Boston Massachusetts USA; ^5^ OPEN Health Bethesda Maryland USA; ^6^ Department of Hematology/Oncology, School of Medicine, Robert C. Byrd Health Sciences Center [North] West Virginia University Morgantown West Virginia USA; ^7^ Department of Epidemiology and Biostatistics, School of Public Health, Robert C. Byrd Health Sciences Center West Virginia University Morgantown West Virginia USA; ^8^ School of Public and Population Health Boise State University Boise Idaho USA

**Keywords:** disease‐free survival, disease‐specific survival, local recurrence, Merkel cell carcinoma, meta‐analysis, overall survival, regional recurrence

## Abstract

**Objective:**

The lack of consensus on the benefits and harms of standard therapies, including surgery (SRx), radiotherapy (RTx), chemotherapy (CTx), and their combinations among early‐stage MCC, prompted this study.

**Methods:**

A systematic review and meta‐analysis of randomized and non‐randomized studies published between January 01, 1972, and January 31, 2023, and having overall survival (OS), local recurrence (LR), regional recurrence (RR), disease‐specific survival (DSS), and/or disease‐free survival (DFS) as outcomes was conducted using the Cochrane Central Register of Controlled Trials (CENTRAL), PubMed (NCBI), Scopus (ELSEVIER), and Web of Science (CLAVIRATE) databases. Hazard ratios (HRs) and their variances were pooled using the inverse variance heterogeneity model.

**Results:**

Forty‐nine studies representing 46,215 participants were included in the meta‐analysis. A statistically significant improvement in OS was observed for groups administered adjuvant RTx (SRx + RTx) compared to SRx only (HR = 0.78, 95% CI, 0.62–0.99), albeit with statistically significant heterogeneity (*Q* = 532.30, *p* < 0.001) and a large amount of inconsistency (*I* ^*2*^ = 94%, 95% CI, 93.0–95.5). Both LR (HR = 1.52, 95% CI, 0.37–6.19) and RR (HR = 0.41, 95% CI, 0.09–1.78) were not statistically significant. In addition, DSS (HR = 0.58, 95% CI, 0.24–1.40) was not statistically significant but DFS was (HR = 0.35, 95% CI, 0.13–0.93). Subgroup analyses revealed that adjuvant radiotherapy was more effective in local than regional MCC. The E‐value suggested that the RTx dose was a confounder of the observed effectiveness of adjuvant RTx; and also, the use of CTx following adjuvant RTx, did not impact the strength of evidence for OS.

**Conclusions:**

Although adjuvant RTx improves survival and recurrence outcomes among early‐stage MCC, the safety and effectiveness of standard therapies in MCC remains poorly studied and, thus, affects the synthesis of evidence across important patient and clinical characteristics. Future research on the comparative effectiveness of different therapies is needed.

## 1 Introduction

Merkel cell carcinoma (MCC) is a rare and highly aggressive cutaneous neuroendocrine carcinoma [1]. Although rare, MCC incidence rates are increasing worldwide, especially among the older population. The annual incidence rate of MCC was 0.13 per 100,000 inhabitants in the European Union between 1995 and 2002 [2]. Post‐2000s, it increased to 0.31 per 100,000 in Denmark, 0.33 per 100,000 in Sweden, and 1.15 per 100,000 persons in Italy [3, 4, 5].

Since 2000, MCC incidence has increased by 95% in the United States, and this is attributed to an aging population, improved diagnostic accuracy, and, possibly, an increase in the prevalence of known risk factors (age, exposure to ultraviolet (UV) radiation, Merkel cell polyomavirus) [6]. In 2016, the incidence rate was 1.03 among men and 0.45 per 100,000 persons among women, denoting an average annual percentage change of 2.7% in the U.S. [7].

Although the majority of MCC patients present with local disease (66%) [8], treatment‐focused research has been limited to late and advanced stages [9]. This research gap remains despite evidence that early and aggressive treatment of MCC results in better disease control. For example, it is estimated that 26%–36% of lesions present with nodal metastasis at diagnosis, whereas 6%–16% have synchronous distant metastasis [10]. Overall, up to 50% of patients present with nodal and distant stages at diagnosis, potentially leaving the other half with early‐stage MCC [11]. The high rate of disease progression in MCC and its proclivity to metastasize should, in principle, make treatment for early stages a priority, given that progression to metastatic MCC is observed within 3 years post‐diagnosis [12]. In addition, it has been reported that ~80% of all recurrences (locoregional or distant) also occur 2–3 years post‐diagnosis [13, 14].

**FIGURE 1:**
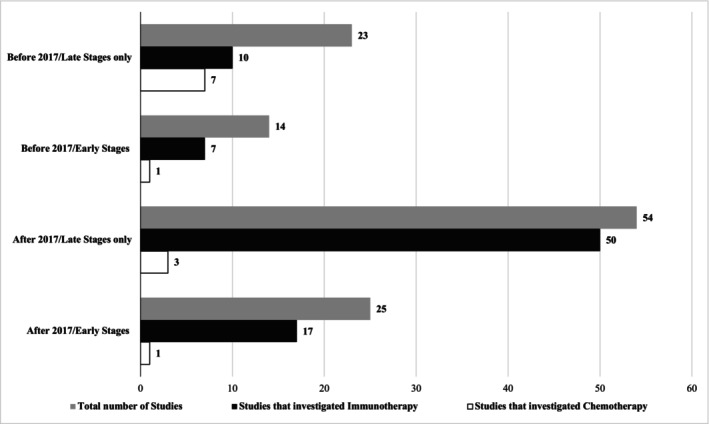
Distribution of RCTs and observational studies registered in clinicaltrials.gov and based on MCC treatment and staging. Studies with early stages included also MCC with other stages, whereas studies with late stages featured only patients with late or advanced stages.

An in‐depth screening of the U.S. clinical trials online registry (clinicaltrials.gov) by the first two authors (Yves Paul Mbous‐YPM and Rowida Mohamed‐RM) found that the vast majority of studies focused on advanced and late‐stage MCC (Figure 1). Since its inception, this registry has included 115 randomized and non‐randomized studies on MCC. Of the randomized controlled trials (RCTs) and non‐randomized studies pre‐2017 (2017 being the year of approval of avelumab for MCC), 14 (50%) included early‐stage MCC, three (10.7%) featured locoregional and metastatic stages, and 11 (39.3%) included all stages. The leading investigatory treatment was immunotherapy (*N* = 14, 50%; 57% of which were immune checkpoint inhibitors ‐ICIs). After 2017, 21 RCTs (24%) included early MCC stages alongside other stages. Interestingly, pre‐2017, 14 (50%) trials had investigated the efficacy of new drugs in early MCC. Post‐2017, 21 studies (24%) included, in some capacity, early‐stage MCC patients. Only post‐2017 did we find trials that strictly included patients with local MCC (2.3%).

Pre‐ or post‐2017, ICIs remain the most investigated treatment in local MCC, with less attention given to standard regimens such as surgery (SRx), radiotherapy (RTx), or chemotherapy (CTx). The effectiveness of the standard of care thus remains unelucidated, to the detriment of locoregionally staged MCC that must contend with secondary evidence (observational studies) to choose the best management pathway. This is perhaps one of the reasons why scientists at the 2018 International Workshop on MCC Research (IWMCC) called for prioritizing research areas that focused on the role of RTx and the optimal management for early‐stage MCC [1]. Also, among RCTs and non‐randomized studies conducted among early‐stage MCC post‐2017, ICIs were the most commonly tested agents. If this trend indicates an eventual increase in the uptake of immunotherapy in the therapeutic market for early‐stage MCC, then it is even more imperative to assess the current effectiveness of standard treatments. Meanwhile, an optimal treatment algorithm for patients with early‐stage MCC remains uncertain. For instance, adjuvant RTx and/or CTx are sometimes used to remove residual disease in order to reduce the risk of recurrence. Unfortunately, the value of RTx in locoregional MCC as monotherapy or as a combination therapy complement to CTx is highly debated. Even a comparison of SRx against SRx with adjuvant RTx is plagued by selection bias as only patients with primary MCC typically receive SRx, and are thus likely to have smaller tumor sizes and/or restricted tumor growth compared to those with locoregional disease [15, 16]. Adjuvant RTx is certainly recommended for nodal MCC; however, in light of mixed results on the clinical benefits of RTx, the NCCN Guidelines encourage enrollment in a clinical trial or neoadjuvant immunotherapy [17]. Adjuvant systemic therapy (CTx) is not reported to provide any survival benefit, and is not routinely recommended [17]. In light of this uncertainty, no consensus exists on the best quality of care per stage among patients with MCC, and no comparative systematic review or meta‐analysis has investigated the survival benefit that SRx, RTx, or CTx confers compared to one another or a combination, in the setting of early‐stage MCC.

While three previous systematic reviews have examined the safety and effectiveness of these modalities in early MCC stages, their analyses were limited by one or more of the following elements: (1) review of peer‐reviewed materials published from 2014 to 2019 [18]; (2) no quantitative summary conducted [18]; (3) where meta‐analysis was conducted, it featured recurrence and mortality outcomes but did not stratify findings according to MCC stages or other important tumor characteristics [19]; (4) inclusion of studies with Stage IV MCC [20]; (5) no clear delineation of treatment interventions (CTx + RTx was sometimes treated as just RTx), and no moderator analysis was conducted to measure the influence of the use of other treatments [19, 20]; (6) used a “less accurate” random‐effects model to pool effect sizes [19, 20]; and (7) did not focus on safety among early MCC stages. In the present work, we seek to fill these gaps by reviewing studies that have compared the benefits and harms of standard therapies in early‐stage MCC in order to fully comprehend the benefits they provide to patients as well as the limits faced by clinicians in this sphere. We use a systematic review and aggregate data meta‐analytic approach to investigate the effectiveness and safety of therapeutic agents and eligible comparators for treating early‐stage MCC. The current study will also employ an updated, more rigorous design than previous systematic reviews. First, we compile all existing evidence on MCC dating back to 1972, the year of the first published report on MCC [21] through 31st January 2023. Second, we included information on additional survival and recurrence outcomes as well as moderator analyses. We hope to make this evidence relevant to clinicians and policymakers by stratifying across stages and other tumor characteristics (of relevance to clinical practice and sometimes used in lieu of staging, including tumor depth, size, number of nodes, etc.) in an effort to provide practical, evidence‐based information on current therapies, including their shortcomings.

## 2 Methods

### 2.1 Overview

We followed the general guidelines of the Cochrane Collaboration [22], Preferred Reporting Items for Systematic Reviews and Meta‐Analyses literature search extension statement (PRISMA‐S) [23], and Preferred Reporting Items for Systematic Reviews and Meta‐Analyses (PRISMA) [23, 24] for this work. The protocol for this study was registered in the Open Science Framework (osf.io/2zfcn). Post hoc changes from our protocol are described throughout the methods. A PRISMA checklist can be found in Appendix S1.

### 2.2 Study Eligibility Criteria

Eligible studies included the following: (1) adult patients (18 years or older) with a diagnosis of early MCC (Stage I, II, or III); (2) included one of the following treatment interventions: “SRx”, “RTx”, “CTx”, “SRx + RTx”, “SRx + CTx”, “SRx + CRTx (chemoradiotherapy)”, or “RTx + CTx” or comparators “SRx”, “RTx”, “CTx”, or biopsy; (3) patients who were either naïve or refractory to the aforementioned interventions; (4) randomized controlled trials (RCTs), prospective cohort, retrospective cohort, case–control, and case‐series designs; and (5) published in the English language. We abided by the staging of the Updated American Joint Committee on Cancer, *8th Edition*. We converted old staging systems, wherever possible, to the corresponding stages or the overarching strata (local, regional) [25]. Exclusion criteria included studies with a case report design, unpublished work (unpublished theses, dissertations, editorials, commentaries, etc.), and studies published in non‐English language.

### 2.3 Study Search and Selection

To the best of our knowledge, the first report on MCC was published in 1972 [21]. Thus, our search included peer‐reviewed publications from 01st January 1972 until 31st January 2023. Four databases were searched by YPM and RM to identify eligible studies: PubMed (NCBI), Scopus (ELSEVIER), Cochrane Central Register of Controlled Trials (CENTRAL), and Web of Science (CLARIVATE). Reference harvesting of selected articles was also conducted. Individual database search strategies are detailed in Appendix S2. Initial results from each database were imported by YPM into Mendeley reference management software (version 1.19.4, Elsevier, London, UK) and then exported into a Microsoft Excel worksheet (v.16.37, 2020, Office 365; Microsoft Corporation, Redmond, Washington, USA) using JabRef bibliographic software (v5.1, the JabRef Development team, open‐sourced). Duplicates were removed both electronically and manually by YPM. The precision of the searches was calculated by dividing the number of studies included by the overall number of studies screened. The Number‐Needed‐to Read (NNR) was then calculated as the reciprocal of the precision [26]. YPM and RM reviewed studies for eligibility by screening the titles and abstracts based on the eligibility criteria. If the inclusion/exclusion decision could not be made based on the title and abstract, the full texts were retrieved for further examination. The reasons for exclusion were recorded and categorized according to the patient/population, intervention, comparison, outcome(s), and study design/setting (PICOS) framework. Disagreements were resolved by GAK. Inter‐rater agreement was assessed using Gwet's AC1 statistic [27].

### 2.4 Data Abstraction

An electronic codebook was initially developed by YPM using an Excel spreadsheet (version 16.37) based on elements derived from previous research [28, 29]. The codebook was then pilot‐tested and reviewed by the research team. Detailed information regarding the elements extracted is described in our protocol (osf.io/2zfcn). The primary outcomes were overall survival (OS), disease‐specific survival (DSS), disease‐free survival (DFS), local recurrence (LR), regional recurrence (RR), distant recurrence (DR), local recurrence‐free survival (LRFS), regional recurrence‐free survival (RRFS), distant recurrence‐free survival (DRFS), and safety measures. The safety measures included the onset, frequency, and duration of any adverse event (AE), serious or otherwise, any grade 3–4 AE, treatment‐related AE, treatment withdrawals, as well as treatment withdrawals as a result of AE and/or death. Secondary outcomes included recurrence‐free survival (RFS), time to progression, response rate (RR), and duration of response (DoR), provided concomitantly with primary outcomes. The extracted outcome data included post‐treatment values. YPM and RM extracted data from each selected article independently. Any disagreements were discussed and resolved until a 100% agreement was reached, and if not, consultation with GAK. Prior to resolving any discrepant items, inter‐rater agreement was assessed using Gwet's AC1 statistic [27].

### 2.5 Risk of Bias, Strength/Certainty of Evidence

The risk of bias was assessed independently using the MethodologicAl Standards for Epidemiological Research (MASTER) instrument [30]. Any disagreements were discussed and resolved until a 100% agreement was reached. Inter‐rater agreement was assessed using Gwet's AC1 statistic [27]. The overall strength/certainty of evidence among the included studies was evaluated using the Grading of Recommendations Assessment, Development, and Evaluation (GRADE) instrument [31]. All assessments were conducted independently by YPM and RM. Disagreements were resolved by consensus and, if necessary, consultation with GAK. Inter‐rater agreement was assessed using Gwet's AC1 statistic [27].

### 2.6 Statistical Analysis

#### 2.6.1 Effect Size Calculation and Pooling

Per best practices, the quantitative analysis for each outcome, i.e., meta‐analysis, was limited to those outcomes in which there were at least six effect sizes. This criterion resulted in omitting the safety outcomes for meta‐analysis, leaving OS, LR, RR, DSS, and DFS for pooling. In addition, a post hoc modification of our protocol entailed the use of hazard ratios (HR) and their variances as our individual effect size metric versus the standardized effect size. In studies, where hazard ratios were not explicitly provided, they were approximated using methods described by Parmar and Stewart [32]. The post hoc decision to use HR was based on the belief that it would facilitate the interpretation of our quantitative analysis. OS represents the overall risk of death over a certain point in time, and thus, an HR > 1 indicates a shorter survival than the control‐referenced group and vice‐versa. The same can be said for DSS, the risk of death due to MCC, and DFS, the risk of recurrence over a specific period of time. LR refers to the risk of developing (lack of control over) local recurrence, and RR denotes the risk of developing (lack of control over) regional recurrence. Calculations were refined to match the intended reference group to blend with these definitions.

The inverse variance heterogeneity (IVhet) model was used to pool effect sizes for all outcomes, and 95% confidence intervals (CIs) were also generated. We chose the IVhet model given that it has been shown to be more accurate with respect to 95% confidence interval coverage than other models such as the traditional random‐effects model of Dersimonian and Laird, as well as the restricted maximum likelihood model [33, 34, 35]. In addition, a recent article recommended the IVhet model for pooling results for an aggregate data meta‐analysis [36]. Non‐overlapping 95% CIs were considered statistically significant. Forest plots were used to display individual and pooled point estimates.

#### 2.6.2 Stability and Validity of Outcomes

Heterogeneity was examined using the Q statistic, with *p* ≤ 0.10 considered statistically significant [37]. Inconsistency was examined using I‐squared (*I* ^*2*^). Values < 25%, 25%–50%, and > 50% were considered to represent small, medium, and large amounts of between‐study inconsistency, respectively [38]. Two‐tailed z alpha values ≤ 0.05 and non‐overlapping 95% confidence intervals were considered statistically significant. Small‐study effects (e.g., publication bias) were assessed qualitatively using the Doi plot and quantitatively using the Luis Furuya‐Kanamori (LFK) index [39]. LFK values of ±1, between ±1 and ±2, and > ±2 were used to indicate no, minor, and major asymmetry, respectively [39]. Influence analyses were conducted to examine the influence of each study on the overall results. A cumulative meta‐analysis, ranked by year, was performed to examine the accumulation of results over time [40]. Outlier analysis was also conducted by excluding results for those effect sizes in which their 95% confidence intervals fell entirely outside the pooled 95% CI. Subgroup analyses were conducted for outcomes with at least four effect sizes per group, as recommended by the Cochrane Handbook [41]. This included OS, RR, DSS, and DFS. These subgroup analyses were conducted according to geographical region, estimation method of HRs, and staging (local, regional, locoregional, distant) wherever possible. Stratification per stage conformed to the definition of local, regional, locoregional, and distant stages as defined in the American Joint Committee on Cancer (AJCC) Staging Manual, *8th Edition* [25]. Conversion to the AJCC *8th Edition* staging system was made for studies that used previous systems. To allow for what might be expected if a new trial was conducted, a post hoc decision was made to calculate 95% prediction intervals (PIs) as well as a *z*‐test for subgroup differences. Two‐tailed alpha values < 0.05 were used to determine between‐group statistical significance.

#### 2.6.3 Meta‐Regression

Meta‐regression analyses based on the IVhet model were used to examine the relationship between OS, LR, RR, DSS, DFS, and selected potential predictor covariates (age, stage, anatomic site, type of treatment used, follow‐up length, tumor depth, RTx dose, number of participants, etc.) listed in full detail in our protocol (osf.io/2zfcn). Analyses were limited to variables with at least 10 effect sizes for a continuous covariate and four effect sizes per group for a categorical covariate [42]. Two‐tailed z‐values ≤ 0.05 and non‐overlapping 95% CIs were considered to represent a statistically significant association. The following software was used for analysis: Stata (version 16), including the user‐written “metan” and LFK routines within Stata; Mendeley (version 1.19.4); JabRef (version 5.1); Microsoft Excel (version 16.37) with the add‐in for Excel, MetaXL (version 5.3); and the Comprehensive Meta‐Analysis Prediction Intervals worksheet.

#### 2.6.4 Unmeasured Confounding

To account for unmeasured confounding in observational studies, the E‐value was calculated based on the pooled value for the following outcomes: OS, LR, RR, DSS, and DFS [43]. The E‐value is a type of sensitivity analysis used to evaluate the minimum strength of association, on the risk ratio scale, that an unmeasured confounder would need to have with both the outcome and treatment to nullify the observed effect [43].

## 3 Results

### 3.1 Study Characteristics

**FIGURE 2:**
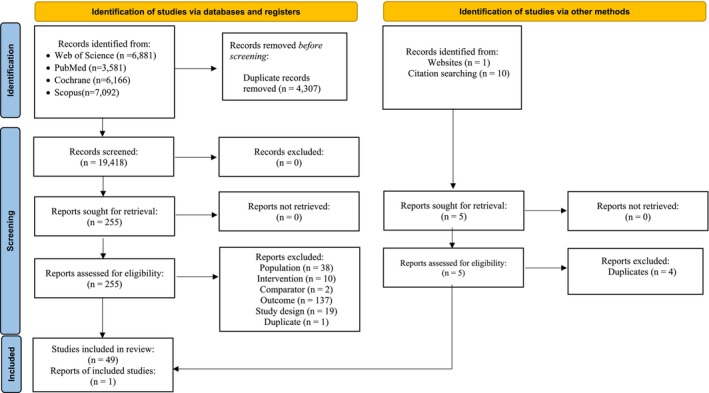
Flowch, art of screening and selection process leading to the final study pool.

A flow diagram depicting the results of the screening process is shown in Figure 2. Gwet's AC1 statistics, a measure of inter‐rater reliability [44] for title/abstract and full‐text screening, were 0.99 and 0.96, respectively. Of the 19,418 abstracts and citations and 255 full texts examined, 49 articles were selected for systematic review [15, 16, 45, 46, 47, 48, 49, 50, 51, 52, 53, 54, 55, 56, 57, 58, 59, 60, 61, 62, 63, 64, 65, 66, 67, 68, 69, 70, 71, 72, 73, 74, 75, 76, 77, 78, 79, 80, 81, 82, 83, 84, 85, 86, 87, 88, 89, 90], and a subset of 43 articles were chosen for meta‐analysis [15, 16, 45, 46, 47, 48, 49, 50, 51, 52, 53, 54, 55, 56, 57, 58, 59, 60, 61, 62, 63, 64, 65, 66, 67, 68, 69, 70, 71, 72, 73, 74, 75, 76, 78, 79, 83, 84, 86, 87, 88, 89, 90]. Appendix S2 includes the list of excluded studies at the full‐text screening step with reasons for exclusion. At the full‐text screening stage, reasons for exclusion included the following: a different cancer population, lack of comparator treatments [91], different types or no emphasis on treatments, used [13, 92], outcomes reported for a combination therapy with no stage stratification [93], duplicates of previously selected studies [94], and different study designs (disease‐specific) [95]. Twenty‐seven selected studies were conducted in the U.S. [16, 45, 46, 49, 55, 56, 58, 61, 62, 63, 64, 66, 68, 69, 70, 71, 72, 75, 76, 77, 79, 80, 81, 83, 84, 87, 88]. The remaining studies were conducted outside the U.S.: Australia (*n* = 10) [47, 51, 54, 60, 65, 73, 74, 78, 85], Canada (*n* = 1) [52], France (*n* = 5) [15, 48, 53, 67, 89], Germany (*n* = 2) [50, 90], Italy (*n* = 1) [57], Sweden (*n* = 1) [86], the Netherlands (*n* = 1) [59], and the United Kingdom (*n* = 1) [82]. Only two studies used a RCT design [53, 85], while the remainder used a retrospective cohort design [15, 16, 45, 46, 47, 48, 49, 50, 51, 52, 54, 55, 56, 57, 58, 59, 60, 61, 62, 63, 64, 65, 66, 67, 68, 69, 70, 71, 72, 73, 74, 75, 76, 77, 78, 79, 80, 81, 82, 83, 84, 86, 87, 88, 89, 90, 96]. The NNR was 396 articles for every one article included, with a precision of 0.25%. The studies were published between 1990 and 2022.

### 3.2 Participant Characteristics

Appendix S3 shows patients' characteristics. Information on the type of facilities that provided care to MCC patients was provided in nine cases. These included academic medical centers (*n* = 9) [45, 48, 50, 58, 75, 77, 84, 88, 90], community medical centers (*n* = 1) [75], private medical centers (*n* = 1) [45], and others (*n* = 1) [88]. Academic centers provided care to at least 42.8%, and to at most 100% of patients in the respective studies. Community medical centers catered to 45% of included patients, whereas other medical centers provided care to 45.8% of included MCC patients. The number of patients per study ranged from 18 [65] to 14,414 [72]. The median age of participants ranged from 65 [16] to 83 years [48]. The smallest sample of male MCC patients was 24% [64] while the highest was 83.3% [46]. Twelve studies included various types of information on race [46, 52, 56, 61, 62, 68, 71, 75, 76, 81, 84, 88]. Five studies described races other than Whites [62, 75, 84, 88], and one presented race as a composite of Whites and Non‐Whites groups [68]. Whites were the predominant racial group, making up between 85% and 100% of all MCC patients in each study. Education level was not explicitly provided but was used in one study as a confounding factor in the prediction of receipt of radiotherapy [97]. Income levels were reported across two studies [75, 84], with the majority of patients earning at least a median annual household income of $48,000 (59.3%–71%) [75, 84]. Marital status was provided in only one study, with 61.3% of participants reported as being married [56]. Seventeen studies provided information on the immune status of their MCC population [15, 16, 48, 49, 51, 52, 54, 55, 66, 67, 69, 70, 74, 75, 77, 80, 81], sixteen of which included immunosuppressed patients in their analysis [15, 16, 48, 49, 51, 52, 54, 55, 66, 67, 69, 70, 74, 75, 77, 80, 81]. The number of immunocompromised patients ranged between 1 and 191 [74, 77]. Histological analyses of Merkel cell polyomavirus (McPyV) were provided in two studies [48, 86]. Immunochemistry analyses were used at diagnosis across 10 studies [45, 51, 53, 58, 66, 67, 69, 70, 76, 80], whereas imaging (computerized tomography, positron emission tomography, ultrasound) was used at diagnosis in 13 studies [48, 50, 51, 52, 53, 54, 64, 65, 67, 70, 73, 76, 85]. Sentinel lymph node biopsy was reportedly used across 19 studies [15, 16, 45, 48, 50, 52, 55, 56, 59, 66, 67, 69, 70, 75, 76, 80, 81, 89]. The tumor depth was reported in 21 studies [45, 49, 51, 54, 55, 57, 58, 61, 63, 64, 69, 70, 71, 73, 74, 76, 77, 80, 84, 87, 89], and ranged from 4.5 mm to 37 mm (median) [63, 77], whereas the median tumor diameter (reported across 21 studies) ranged from 4.5 mm to 100 mm [55, 84].

### 3.3 Treatment Characteristics

Forty‐four studies reported the use of surgery (Appendix S3) [15, 16, 45, 46, 47, 48, 49, 50, 51, 52, 53, 54, 55, 56, 57, 58, 59, 60, 61, 62, 64, 65, 66, 67, 68, 69, 70, 71, 72, 73, 74, 75, 76, 77, 78, 79, 80, 81, 84, 86, 87, 88, 89, 90]. Different surgical procedures were used, including wide local excision—WLE (*n* = 20), narrow local excision (*n* = 2), lymph node dissection (*n* = 2), lymphadenectomy (*n* = 2), Mohs micrographic surgery ‐MMS (*n* = 6), and resection (*n* = 2). The most common surgical margins were between 1 and 2 cm (*n* = 10) out of twenty studies that reported it. Adjuvant RTx was used across 41 studies [15, 16, 45, 46, 47, 48, 49, 50, 51, 52, 53, 54, 55, 56, 57, 58, 59, 60, 61, 62, 65, 66, 67, 68, 69, 70, 71, 72, 73, 74, 75, 78, 79, 80, 81, 84, 86, 87, 88, 89, 90]. Overall, the RTx total prescribed dose ranged from 45 to 66 Gy [86, 87]. Total prescribed doses administered to the primary tumor ranged from 40 to 55 Gy [87, 89], whereas regional nodes were administered total prescribed doses between 45 to 60 Gy [87]. The predominant RTx fields were 3–5 cm margins (*n* = 41). Prophylactic RTx administered to the nodal basin or definitive RTx (RTx used as sole monotherapy) was provided in 15 studies [45, 46, 49, 51, 53, 54, 60, 62, 63, 64, 66, 81, 84, 89, 98]. MCC patients received on average five fractions of RTx per week (*n* = 8) [50, 63, 64, 65, 66, 86, 89, 90] with average doses of 2 Gy per fraction (*n* = 10) [51, 53, 54, 62, 63, 64, 73, 74, 86, 89]. Twenty‐four studies reported the use of CTx [45, 47, 48, 49, 51, 52, 54, 58, 60, 63, 65, 66, 69, 70, 73, 74, 78, 79, 81, 84, 85, 88, 90]. Eleven studies reported the names of the agents used for systemic therapy. The most common drugs used included etoposide (*n* = 7), carboplatin (*n* = 5), and cisplatin (*n* = 2). The median follow‐up post‐diagnosis ranged from 16 months to 48 months [45, 63].

### 3.4 Risk of Bias and GRADE Assessment

**FIGURE 3:**
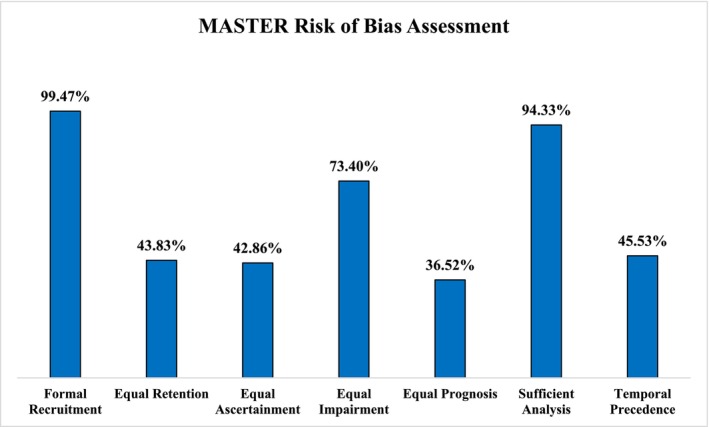
Pooled risk of bias of the selected studies.

The overall agreement rate, as measured by the Gwet AC1 statistic, was 0.94 for the MASTER assessment. In Figure 3, The MASTER risk of bias assessment informs on the percentage of studies that met each of the safeguards pertaining to formal recruitment, equal retention, equal ascertainment, equal impairment, equal prognosis, sufficient analysis, and temporal precedence. As the domains of bias differ (selection, information, analytic, confounding, and design‐related bias) among safeguards, the results shown represent the percentages of studies that, on average, scored “1” for all domain items per safeguard. A total of 99.5% and 94.3% of our selected studies fulfilled the criteria for formal recruitment and sufficient analysis, respectively. Only 36.5% of studies met all criteria for equal prognosis. Less than 50% of included studies satisfied the requirements for equal retention (43.8%), equal ascertainment (42.8%), and temporal precedence (45.5%).

GRADE results, shown in Appendix S4, reinforce the risk of bias results as the certainty of the evidence provided across all outcomes ranged from “Low” to “Moderate” given the serious risk of bias and inconsistency recorded.

### 3.5 Outcome Assessment

#### 3.5.1 Changes in OS

**FIGURE 4:**
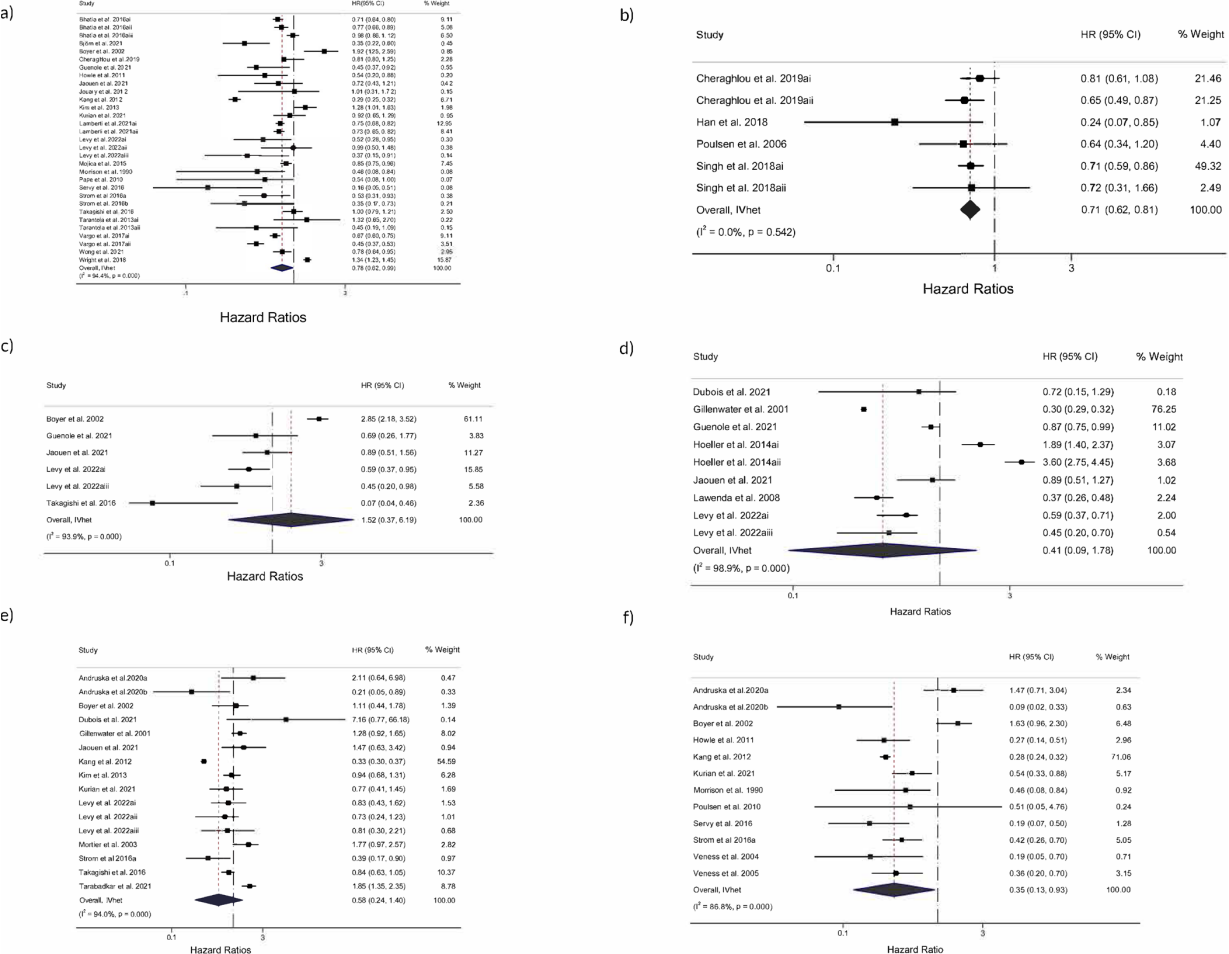
Forest plot for HRs for changes in (a) OS for adjuvant RTx, (b) OS for addition of CTx, (c) LR, (d) RR, (e) DSS, and (f) DFS. The black‐filled squares, sized according to the weight contributing to the overall effect, represent changes in HR from each study while the left and right extremes of the squares represent the lower and upper 95% confidence intervals for changes in outcome (OS, LR, RR, DSS, DFS) from each study. The black diamond represents the pooled effect size change in outcome (OS, LR, RR, DSS, DFS) while the left and right extremes of the diamonds represent the pooled lower and upper 95% confidence intervals for changes in outcome. The red dashed vertical line through the middle of the diamond represents the pooled mean effect while the black dashed vertical line represents the zero (0) point. % Weight: Percentage weight of a particular study.

Thirty‐one studies included extractable data to quantitatively summarize the pooled changes in OS (hazard ratios), as shown in the forest plot of Figure 4a. SRx + RTx showed a statistically significant reduced risk of death (HR = 0.78, 95% CI, 0.62–0.99), statistically significant heterogeneity (Q = 532.30, *p* < 0.001), and a large amount of inconsistency (*I* ^*2*^ = 94%, 95% CI, 93.0–95.5) compared to SRx only. The absolute between‐study variance of the true effect size, tau‐squared ($\tau^{2}$), was 0.16, while the 95% PI for what might be expected if a new trial was conducted in similar populations included one (95% PI, 0.33–1.83). Minor asymmetry suggestive of small‐study effects (publication bias, etc.) was observed (LFK = −1.67, Appendix S5: Figure 1a). Although seven outliers were detected, their deletion from the model did not have a major effect on the overall findings, although they contracted the 95% CI (HR = 0.77, 95% CI, 0.70–0.84, *Q* = 53.97, *p* < 0.001, *I* ^*2*^ = 57.4%, 95% CI, 32.9–72.9). Influence analysis with each study deleted from the model once showed that results were statistically significant across all but nine deletions, with all nine deletions slightly including or overlapping with one (Appendix S5: Figure 2a). Cumulative meta‐analysis revealed that results were statistically significant in 2017 and from 2021 [75] onwards (Appendix S5: Figure 3a).

As shown in Figure 4b, there was a statistically significant reduced risk of death with the use of CTx (HR = 0.71, 95% CI, 0.62–0.81), statistically non‐significant heterogeneity (*Q* = 4.05, *p* = 0.54), and no inconsistency (*I* ^*2*^ = 0%, 95% CI, 0–68.7). $\tau^{2}$ was 0. The 95% PI (0.62–0.81) revealed what one might expect if they conducted their own study. No asymmetry was observed (LFK = −0.83, Appendix S5: Figure 1b). No outliers were detected. Influence analysis showed that results were statistically significant across all deletions (Appendix S5: Figure 2b). Cumulative meta‐analysis revealed that results have been statistically significant since 2018 (Appendix S5: Figure 3b).

#### 3.5.2 Changes in LR

Six studies quantitatively compared LR between SRx and SRx + RTx (Figure 4c). Across six studies, the adjuvant use of RTx increased the proportion of local recurrences among MCC patients. There was an increased risk of LR, but it was not statistically significant (HR = 1.52, 95% CI, 0.37–6.19). Statistically significant heterogeneity (*Q* = 65.2, *p* < 0.001) and a large amount of inconsistency (*I* ^*2*^ = 93.9%, 95% CI, 86.1–95.9) were observed. $\tau^{2}$ was 0.95 while the 95% PI was 0.06–43.93. No asymmetry was observed (LFK = 0.64, Appendix S5: Figure 1c). In addition, no outliers were detected. Results were not statistically significant when each study was deleted from the model (Appendix S5: Figure 2c). Cumulative meta‐analysis revealed that results were not statistically significant across all years (Appendix S5: Figure 3c).

#### 3.5.3 Changes in RR

Nine studies compared quantitatively the proportion of regional recurrence between SRx and SRx + RTx (Figure 4d). There was no statistically significant reduced risk of RR (HR = 0.41, 95% CI, 0.09–1.78), but statistically significant heterogeneity (*Q* = 699.3, *p* < 0.001), and a large amount of inconsistency (*I* ^*2*^ = 98.9%, 95% CI, 98.5–99.1) with the use of adjuvant RTx was observed. $\tau^{2}$ was 0.95. The 95% PIs ranged from 0.02 to 7.50. No asymmetry was observed (LFK = −0.27, Appendix S5: Figure 1d). One outlier was detected, and its removal did not affect the results (HR = 0.38, 95% CI, 0.11–1.25, Q = 371.2, *p* < 0.001, *I* ^*2*^ = 98.1%, 95% CI, 97.4–98.6). Influence analysis with each study deleted from the model once showed that results were not statistically significant across all deletions (Appendix S5: Figure 2d). Cumulative meta‐analysis revealed that results were not statistically significant across all years (Appendix S5: Figure 3d).

#### 3.5.4 Changes in DSS

Sixteen studies compared SRx vs. SRx + RTx in terms of DSS (Figure 4e). There was no statistically significant reduction in the risk of death by MCC (HR = 0.58, 95% CI, 0.24 to 1.40). Statistically significant heterogeneity (*Q* = 249.8, *p* < 0.001) and a large amount of inconsistency (*I* ^*2*^ = 94.0%, 95% CI, 91.7–95.7) with the use of adjuvant RTx were observed. $\tau^{2}$ was 0.61. The 95% PI included one (95% PI, 0.08–4.01). Minor asymmetry was observed (LFK = −1.15, Appendix S5: Figure 1e). No outliers were detected. None of the results were statistically significant when each study was deleted from the model once (Appendix S5: Figure 2e). Cumulative meta‐analysis showed that results were not statistically significant across the years (Appendix S5, Figure 3e).

#### 3.5.5 Changes in DFS

Twelve studies compared SRx versus SRx + RTx in terms of DFS. Figure 4f shows a statistically significant increase in time from treatment until recurrence (HR = 0.35, 95% CI, 0.13–0.93), statistically significant heterogeneity (*Q* = 83.3, *p* < 0.001), and a large amount of inconsistency (*I* ^*2*^ = 86.8%, 95% CI, 78.7–91.8) with the use of adjuvant RTx. The absolute between‐study variance of the true effect size, tau‐squared ($\tau^{2}$), was 0.48. The 95% PIs did not include one (95% PI, 0.05–2.35). No asymmetry was observed (LFK= –0.33, Appendix S5: Figure 1f). One outlier was detected, yet no major difference was observed after its removal (HR = 0.31, 95% CI 0.16–0.59). In five instances, the results were non‐significant when each study was deleted from the model. (Appendix S5: Figure 2f). Cumulative meta‐analysis showed that results were statistically significant in 2016 [69], and then again in 2021 [52] (Appendix S5: Figure 3f).

#### 3.5.6 Subgroup Analyses

##### 3.5.6.1 Changes in OS

Subgroup analyses for changes in OS can be found in (Appendix S5: Figure 4a). For studies conducted in the US versus other countries, HRs were 0.88 (95% CI, 0.70 to 1.11, *Q* = 235.7, *p* < 0.001, *I* ^*2*^ = 92.8%) and 0.59 (95% CI, 0.36–0.99, *Q* = 180.6, *p* < 0.001, *I* ^*2*^ = 93.4%), respectively. Stage‐wise, HRs were 0.72 (95% CI, 0.60–0.87, *Q* = 77.5, *p* < 0.001, *I* ^*2*^ = 84.5%), 1.17 (95% CI, 0.81–1.69, *Q* = 33.3, *p* < 0.001, *I* ^*2*^ = 91.0%) and 0.63 (95% CI, 0.38–1.05, *Q* = 227.7, *p* < 0.001, *I* ^*2*^ = 94.3%) for local, regional, and locoregional stages, respectively (Appendix S5: Figure 4b). In terms of methodology, HRs were 0.83 (95% CI, 0.69–1.00, *Q* = 250.2, *p* < 0.001, *I* ^*2*^ = 91.6%) and 0.47 (95% CI, 0.16–1.40, *Q* = 172.7, *p* < 0.001, *I* ^*2*^ = 95.4%) respectively for studies which explicitly provided HRs and those for which the HRs had to be estimated (Appendix S5: Figure 4c). *Z*‐tests suggested that changes in OS did not statistically differ across all these subgroups except between local and regional stages (*Z*_diff_ = 2.69, *p* = 0.007). The magnitude of this difference (Diff = 0.3, 95% CI −0.16 to 0.76) suggests that the true difference in OS after the use of adjuvant RTx between local and regional stages probably falls in the range −0.16 to 0.76 and thus included the null (0) (Appendix S6: Table 1).

##### 3.5.6.2 Changes in RR

All subgroup analyses' results for changes in RR can be found in Appendix S5: Figure 5. In terms of methodology, HRs were 0.37 (95% CI, 0.03–4.45, *Q* = 561.4, *p* < 0.001, *I* ^*2*^ = 99.3%) and 0.73 (95% CI, 0.39–1.37, *Q* = 26.2, *p* < 0.001, *I* ^*2*^ = 88.6%) respectively for studies which explicitly provided HRs and those for which the HRs had to be estimated. *Z*‐tests suggested that changes in RR did not statistically differ across all these subgroups (Appendix S6: Table 1).

##### 3.5.6.3 Changes in DSS

In Appendix S5: Figure 6a, subgroup analyses for changes in DSS are shown. In terms of geographical location, HRs were 1.16 (95% CI, 0.81–1.66, *Q* = 34.9, *p* < 0.001, *I* ^*2*^ = 77.1%) and 0.37 (95% CI, 0.11–1.25, *Q* = 36.2, *p* < 0.001, *I* ^*2*^ = 83.4%), respectively for studies conducted in the US versus other countries. In terms of methodology, HRs were 0.99 (95% CI, 0.64–1.53, *Q* = 21.2, *p* < 0.001, *I* ^*2*^ = 57.5%) and 0.52 (95% CI, 0.17–1.64, *Q* = 197.4, *p* < 0.001, *I* ^*2*^ = 97.5%), respectively, for studies which explicitly provided HRs and those for which the HRs had to be estimated (Appendix S5: Figure 6b). *Z*‐tests suggested that changes in DSS differed statistically in terms of methodology (*Z*_Diff_ = 2.7, *p* = 0.007) and geographical location (*Z*_diff_ = 5.99, *p* < 0.001) (Appendix S6: Table 1).

##### 3.5.6.4 Changes in DFS

Subgroup analyses' results for changes in DFS can be found in Appendix S5: Figure 7a. In terms of geographical location, HRs were 0.85 (95% CI, 0.31–2.31, *Q* = 29.5, *p* < 0.001, *I* ^*2*^ = 86.4%) and 0.29 (95% CI, 0.22–0.40, *Q* = 8.19, *p* < 0.22, *I* ^*2*^ = 26.8%), respectively for studies conducted in the US versus other countries. In terms of methodology, HRs were 0.45 (95% CI, 0.26–0.78, *Q* = 20.84, *p* = 0.004, *I* ^*2*^ = 66.4%) and 0.33 (95% CI, 0.06–1.72, *Q* = 57.5, *p* < 0.001, *I* ^*2*^ = 94.8%), respectively, for studies which explicitly provided HRs and those for which the HRs had to be estimated (Appendix S5: Figure 7b). *Z*‐tests suggested that changes in DFS differed statistically in terms of geographical location (*Z*_diff_ = 2.44, *p* = 0.014) (Appendix S6: Table 1).

#### 3.5.7 Meta‐Regression

Meta‐regression results can be found in Appendix S6: Table 2. For changes in OS (SRx vs. SRx + RTx), positive associations were found with sentinel lymph node biopsy (SLNB), tumor depth, the use of MMS, total RTx dose, and the use of CTx, among others. The association with SLNB and the total RTx dose were statistically significant. Negative non‐significant associations were found for the study percentage of males and the receipt of wide local excision (WLE). LR had negative associations with the use of CTx; RR and DSS had a negative association with the use of WLE or MMS, respectively, but DSS had a positive significant association with the use of CTx; DFS had a positive association with MMS and a negative one with the use of CTx.

#### 3.5.8 Unmeasured Confounding

E‐values are shown in Appendix S6: Table 3. For changes in OS between SRx + RTx versus SRx, the E‐value was 1.66 with a corresponding confidence interval of 1.09. This suggests that residual confounding could explain the observed association if there exists an unmeasured moderator that has a relative association as large as 1.66. Looking at the meta‐regression HRs, we found that the total RTx dose with HR of 1.57 (95% CI, 1.14–2.17) could be one of the factors whose association may exceed 1.66. The study percentage of males with an HR of 0.01 or the use of CTx (HR = 1.08) seemed less likely to impact the confound the association between the adjuvant RTx and better outcomes, and, as such, it is likely that RTx effectiveness does not depend on the use of CTx.

#### 3.5.9 Safety

Safety outcomes could not be quantitatively described because although seven studies reported some information on cancer care toxicity [47, 50, 53, 71, 85, 89, 96], only four presented results that could have been summarized (Appendix S3) [47, 53, 85, 96]. The number of patients who experienced toxicity ranged from 0 to 41 [47, 71]. The high reported number of toxic events was consistent with the use of CTx. Only one study reported the number of patients who discontinued therapy because of the experienced toxicity [50]. Toxicity outcomes measured included the frequency of acute or late toxicities (Grade 1,2, 3, or 4 events), white cell toxicity, and skin toxicity.

## 4 Discussion

The pooled effect of HRs showed that using adjuvant RTx led to a statistically significant reduced risk of death and improved DFS among early‐stage MCC patients. Although a beneficial effect was shown for DSS and greater control for regional recurrences, these findings were not statistically significant. However, it should be noted that SRx only achieved better local control than adjuvant RTx. While these results demonstrate the superiority of adjuvant RTx combination therapy, they need to be interpreted carefully due to statistically significant heterogeneity, considerable inconsistency, and small‐study effects. The PIs suggest that using CTx in a new population may benefit early‐stage MCC in terms of OS. However, for LR, RR, DFS, and DSS, in a new population, some may benefit while others may not. Subgroup analyses for all outcomes revealed no statistical difference between methodology for HR calculation (original vs. estimated), geographical region, and statistical significance, despite high inconsistency and significant heterogeneity. There was a statistically significant difference in OS following the use of adjuvant RTx between local and regional MCC, with RTx improving OS more among local than regional. For DSS, there was a statistically significant difference in terms of geographic location (i.e., results obtained from studies conducted in the USA differed significantly from those led in other countries around the world) and HR calculation methodology. DFS differed statistically based on geographical location.

The MASTER risk of bias showed serious concerns with respect to attrition, missing data, and analysis accounting for missing data, blinding of outcome assessors, participants/caregivers and data analysis, randomization process, concealment of allocation procedure, balance of key baseline characteristics across groups, selection before exposure, carry‐over or refractory effects, dose, and duration of intervention influence on outcome. To the best of our knowledge, this is the first application of the MASTER instrument for the analytical design of clinical research studies; therefore, no comparisons exist in the literature. Although serious concerns were uncovered during the MASTER assessment, it is difficult to pinpoint the importance of specific underperforming safeguards in alleviating the extent of bias as they are not equally responsible [30, 99]. The strength of evidence, assessed by GRADE, suggested that the pooled findings were moderate (OS, RR, DFS) and low certainty (LR, DSS). Serious concerns were raised with respect to the risk of bias, inconsistency, and small‐study effects, i.e., publication bias.

Meta‐regression results suggested that the receipt of MMS increased the risk of death when adjuvant RTx was used, whereas WLE reduced the risk of death. Whereas tumor depth and the use of CTx had no effect on OS, the total RTx dose increased the risk of death. A recent study reported an improved OS in MCC patients treated with MMS compared to WLE [100], although conflicting evidence has suggested that OS does not truly differ between MCC treated with MMS and WLE [83, 101]. In terms of RR, MMS showed a positive association with control of nodal MCC recurrence, which aligns with findings in the literature [102]. Previous work also showed that MMS offered better local control with no decrease in MCC‐specific deaths [103], although this could not be evaluated in this study.

In this study, the use of adjuvant RTx created a selection and treatment bias issue that needs to be carefully considered during the interpretation of results. Previous work had suggested that CTx had no effect on OS following the administration of adjuvant RTx [65], which is consistent with our meta‐regression results, where the use of CTx in adjuvant RTx treated MCC had no effect on the OS. As SLNB assists in risk stratification and radiation treatment provision, its positive association with overall and MCC‐specific reduced risk of death was expected (consistent with previous findings) [104].

### 4.1 Evaluation of Results Compared to Previous Systematic Reviews and Meta‐Analyses

Our findings are in agreement with what is found in the literature. A previous systematic review and meta‐analysis of RTx use across all stages found an overall reduced risk of death (HR = 0.81, 95% CI, 0.75–0.86), and a reduced risk of recurrence (0.45, 95% CI, 0.32–0.62). Although the meta‐analysis included studies with Stage IV patients, the vast majority were early‐stage MCC [20]. Another study showed that adjuvant RTx reduced recurrences among primary MCC, as shown throughout this work [19].

### 4.2 Implications for Research

This work highlighted several issues to consider with respect to reporting clinical research studies. Across most studies, even those that linked registry to claims data, there was a lack of socioeconomic factors, including median household income, insurance coverage, education, race/ethnicity, employment status, and marital status. This inadequate reporting extended to methods of diagnosis that were not widely discussed. Other cancer factors, including time since diagnosis, time until therapy, cancer stage, the stratification of tumor size, lymphovascular invasion, and the pathological margins, limited our ability to examine evidence across very important moderators. Cancer treatment, especially in relation to CTx, was not adequately described. The type of RTx administered was not commonly provided (external beam or hypofractionated RTx). These “flaws” are concerning as medical records or electronic health records (the most common data source among our studies) contain more comprehensive information than registry or claims data. To the best of our knowledge, no guideline exists for reporting clinical retrospective or prospective studies on this topic. The closest existing checklist for reporting epidemiological studies, the Strengthening the Reporting of Observational Studies in Epidemiology (STROBE), does not provide a paradigm for eliciting relevant information from observational studies.

### 4.3 Clinical Implications

Our findings suggest that adjuvant RTx may be effective for treating patients with early‐stage MCC compared to surgical monotherapy. On the other hand, no firm conclusion can be taken with respect to CTx as very few studies seek to stratify and compare the outcomes of patients offered different modalities in early‐stage MCC. Further, the safety of these treatments is highly under‐assessed in this population. For all the side effects and long‐term complications of cancer treatment, the fact that safety is not actively reported is deeply concerning.

### 4.4 Study Limitations

The following limitations need to be considered when interpreting our findings: (1) the inability to conduct some pre‐planned analyses, for example, the differential in OS between SRx treatments (WLE and MMS), and quantitative safety assessments; (2) the statistically significant heterogeneity, a large amount of unidentified inconsistency and small‐study effects (publication bias, etc.) observed; (3) the estimation of HRs that may not necessarily completely reflect the observed outcome differential; (4) the inherent biases of the original studies included in this systematic review with meta‐analysis; (5) underreported data that could have assisted in conducting comprehensive meta‐regression analyses, for example, the number of recurrent patients presented with new treatment; and (5) the potential for ecological fallacy, specifically Simpson's paradox, since this was an aggregate data meta‐analysis [105].

## 5 Conclusions

The investigation of the effectiveness and safety of treatment among early‐stage MCC suggests that the highest recorded benefit follows the administration in adjuvant settings of RTx; more so in local MCC than regional or locoregional. However, in this study, we were confronted with serious issues pertaining to the stratification of evidence, the shortage of studies evaluating safety, and the underreporting of critical baseline characteristics.

## Author Contributions

**Yves Paul Vincent Mbous:** conceptualization (lead), data curation (lead), formal analysis (lead), investigation (lead), methodology (lead), project administration (lead), resources (lead), software (lead), validation (equal), visualization (lead), writing – original draft (lead), writing – review and editing (equal). **Rowida Mohamed:** conceptualization (supporting), data curation (lead), formal analysis (supporting), investigation (equal), methodology (equal), resources (supporting), validation (equal), writing – review and editing (equal). **Usha Sambamoorthi:** conceptualization (supporting), investigation (supporting), methodology (supporting), project administration (supporting), supervision (equal), writing – original draft (supporting), writing – review and editing (supporting). **Murtuza Bharmal:** conceptualization (equal), investigation (supporting), methodology (supporting), supervision (supporting), writing – review and editing (supporting). **Khalid M. Kamal:** resources (supporting), validation (supporting), writing – review and editing (supporting). **Traci LeMasters:** resources (supporting), validation (supporting), writing – review and editing (supporting). **Joanna Kolodney:** resources (supporting), validation (supporting), writing – review and editing (supporting). **George A. Kelley:** conceptualization (lead), data curation (equal), formal analysis (lead), investigation (lead), methodology (lead), project administration (lead), resources (lead), supervision (lead), validation (lead), visualization (lead), writing – original draft (lead), writing – review and editing (equal).

## Conflicts of Interest

The authors declare no conflicts of interest.

## Supporting information


Appendix S1.

Appendix S2.

Appendix S3.

Appendix S4.

Appendix S5.

Appendix S6.

## Data Availability

Data sharing is not applicable to this article as no new data were created or analyzed in this study.
