# Supplementary material for: Effectiveness and Safety of Treatments for Early‐Stage Merkel Cell Carcinoma: A Systematic Review and Meta‐Analysis of Randomized and Non‐Randomized Studies
Source: Cancer Med. 2025 Jan 3;14(1):e70553. doi: 10.1002/cam4.70553 (PMC11696246; doi:10.1002/cam4.70553)
Supplement: Supplementary file 2 — Appendix S2. [file CAM4-14-e70553-s006.docx]

|  |  | **Table 1:** Search strategy template for PubMed |  |
| --- | --- | --- | --- |
|  | **Concept** | **Search terms** |  |
|  | Population | *MeSH terms:* merkel cell carcinoma, merkel cells. | |
|  |  | *Free text terms:* merkel cell, merkel cell tumor, merkel cell cancer, merkle tumor, merkel cell carcinoma, merkel cell carcinoma*, merkel cell tumour, merkel cell neoplasm*, small blue cell carcinoma of the skin, trabecular cell carcinoma, neuroendocrine carcinoma of the skin, neuroendocrine, neuroendocrine cancer, neuroendocrine tumour, skin carcinoma, merkel cell*, small cell carcinoma | |
|  |  |  | |
|  | Intervention | *MeSH terms:* general surgery, surgical procedures-operative, surgery, mohs’ surgery, mohs’ micrographic surgery, lymph node excision, dissection, lymph node excision, adjuvant radiotherapy, adjuvant radiation, adjuvant radiation oncology, adjuvant chemotherapy, chemoradiotherapy adjuvant, neoadjuvant therapy | |
|  |  |  | |
|  |  | *Free text terms:* surger*, wide local excision, local excision, nodal dissection irradiation, lymphadenectomy, mohs’ micrographic surgery, mohs’ surgery, moh surgery, moh’s surgery, Moh micrographic surgery, moh* micrographic surgery, chemotherapy, chemotherapy*, adjuvant radiation therapy, adjuvant radiation*, adjuvant radio*, adjuvant chemo*, adjuvant chemotherap*, chemoradiation therapy, adjuvant chemoradiotherapy*. | |
|  |  |  | |
|  |  |  | |
|  | Comparator | *MeSH terms:* chemotherapy, induction chemotherapy, maintenance chemotherapy, consolidation chemotherapy, drug therapy, chemoradiotherapy, radiotherapy, neoadjuvant radiotherapy, radiosurgery, chemoradiotherapy, neoadjuvant therapy. | |
|  |  | *Free text terms:* chemotherapy, induction chemotherapy*, maintenance chemotherapy*, consolidation chemotherapy*, drug therap*, chemoradiotherapy, radiotherapy*, radiosurgery, radiation therapy, radiation oncology, neoadjuvant therap*. | |
|  | Outcomes | *MeSH terms: survival, disease-free survival, disease progression, event-free survival, progression-free survival, survival analysis, proportional hazards models, drug-related side effects and adverse reactions, withholding treatment* | |
|  |  |  | |
|  |  | *Free text terms:* overall survival, progression-free survival, progression free survival, cancer-specific survival, cancer specific survival, disease specific survival, disease specific survival, disease-free survival, disease free survival, relapse-free survival, relapse free survival, recurrence-free survival, hazard ratio, adverse effect, adverse event, treatment withdrawal, treatment withholding. | |
|  |  |  | |
|  | Study |  |  |
|  | design/setting | *MeSH terms*: longitudinal studies, cohort studies, case-control studies randomized controlled trial, controlled clinical trial . | |
|  |  | *Free text terms:* cohort, cohort stud*, historical cohort stud*, concurrent stud*, prospective cohort, prospective stud*, prospective, retrospective cohort, retrospective stud*, retrospective, case-control, case control, case-control study case control study, case-referent stud*, case referent stud*, case-comparison stud*, case comparison stud*,case-base stud*, case base stud*, nested case-control stud*, nested case control stud*, matched case-control stud*, matched case control stud*, randomized clinical trial*, clinical trial, RCT* | |

**Ready for PubMed**

1. merkel cell carcinoma[MeSH Terms]) OR (merkel cells[MeSH Terms]) Filters: from 1970 – 2021.
2. merkel cell[Title/Abstract] OR merkel cell tumor[Title/Abstract] OR merkel cell cancer[Title/Abstract] OR merkle tumor[Title/Abstract] OR merkel cell carcinoma[Title/Abstract] OR merkel cell carcinoma*[Title/Abstract] OR merkel cell tumour[Title/Abstract] OR merkel cell tumor[Title/Abstract] OR merkel cell neoplasm*[Title/Abstract] OR small blue cell carcinoma of the skin[Title/Abstract] OR trabecular cell carcinoma[Title/Abstract] OR neuroendocrine carcinoma of the skin[Title/Abstract] OR neuroendocrine[Title/Abstract] OR neuroendocrine cancer[Title/Abstract] OR neuroendocrine tumour[Title/Abstract] OR neuroendocrine tumor[Title/Abstract] OR skin carcinoma[Title/Abstract] OR merkel cell*[Title/Abstract] OR small cell carcinoma[Title/Abstract].
3. (general surgery[MeSH Terms] OR operative surgical procedures[MeSH Terms] OR surgery[MeSH Terms] OR mohs’ surgery[MeSH Terms] OR mohs’ micrographic surgery[MeSH Terms] OR lymph node excision[MeSH Terms] OR dissection[MeSH Terms] OR lymph node excision[MeSH Terms] OR adjuvant radiotherapy[MeSH Terms] OR adjuvant radiation[MeSH Terms] OR adjuvant radiation oncology[MeSH Terms] OR adjuvant chemotherapy[MeSH Terms] OR chemoradiotherapy adjuvant[MeSH Terms] OR neoadjuvant therapy[MeSH Terms] OR chemotherapy[MeSH Terms] OR induction chemotherapy[MeSH Terms] OR maintenance chemotherapy[MeSH Terms] OR consolidation chemotherapy[MeSH Terms] OR drug therapy[MeSH Terms] OR chemoradiotherapy[MeSH Terms] OR radiotherapy[MeSH Terms] OR neoadjuvant radiotherapy[MeSH Terms] OR radiosurgery[MeSH Terms] OR chemoradiotherapy[MeSH Terms] OR neoadjuvant therapy[MeSH Terms] OR chemotherapy[MeSH Terms] OR induction chemotherapy[MeSH Terms] OR maintenance chemotherapy[MeSH Terms] OR consolidation chemotherapy[MeSH Terms] OR drug therapy[MeSH Terms] OR chemoradiotherapy[MeSH Terms] OR radiotherapy[MeSH Terms] OR neoadjuvant radiotherapy[MeSH Terms] OR radiosurgery[MeSH Terms] OR chemoradiotherapy[MeSH Terms] OR neoadjuvant therapy[MeSH Terms]) Filters: from 1970 – 2021.
4. (surger*[Title/Abstract] OR wide local excision[Title/Abstract] OR local excision[Title/Abstract] OR nodal dissection irradiation[Title/Abstract] OR lymphadenectomy[Title/Abstract] OR mohs’ micrographic surgery[Title/Abstract] OR mohs’ surgery[Title/Abstract] OR moh surgery[Title/Abstract] OR moh’s surgery[Title/Abstract] OR Moh micrographic surgery[Title/Abstract] OR moh* micrographic surgery[Title/Abstract] OR chemotherapy[Title/Abstract] OR chemotherapy*[Title/Abstract] OR adjuvant radiation therapy[Title/Abstract] OR adjuvant radiation*[Title/Abstract] OR adjuvant radio*[Title/Abstract] OR adjuvant chemo*[Title/Abstract] OR adjuvant chemotherap*[Title/Abstract] OR chemoradiation therapy[Title/Abstract] OR adjuvant chemoradiotherapy*[Title/Abstract] OR chemotherapy[Title/Abstract] OR induction chemotherapy*[Title/Abstract] OR maintenance chemotherapy*[Title/Abstract] OR consolidation chemotherapy*[Title/Abstract] OR drug therap*[Title/Abstract] OR chemoradiotherapy[Title/Abstract] OR radiotherapy*[Title/Abstract] OR radiosurgery[Title/Abstract] OR radiation therapy[Title/Abstract] OR radiation oncology[Title/Abstract] OR neoadjuvant therap*[Title/Abstract].)
5. (survival[MeSH Terms] OR disease-free survival[MeSH Terms] OR disease progression[MeSH Terms] OR event-free survival[MeSH Terms] OR progression-free survival[MeSH Terms] OR survival analysis[MeSH Terms] OR proportional hazards models[MeSH Terms]) Filters: from 1970 – 2021.
6. (overall survival[Title/Abstract] OR progression-free survival[Title/Abstract] OR progression free survival[Title/Abstract] OR cancer-specific survival[Title/Abstract] OR cancer specific survival[Title/Abstract] OR disease specific survival[Title/Abstract] OR disease specific survival[Title/Abstract] OR disease-free survival[Title/Abstract] OR disease free survival[Title/Abstract] OR relapse-free survival[Title/Abstract] OR relapse free survival[Title/Abstract] OR recurrence-free survival[Title/Abstract] OR hazard ratio[Title/Abstract])
7. (longitudinal studies[MeSH Terms] OR cohort studies[MeSH Terms] OR case-control studies randomized controlled trial[MeSH Terms] OR controlled clinical trial[MeSH Terms] ) Filters: from 1970 – 2021.
8. (cohort[Title/Abstract] OR cohort stud*[Title/Abstract] OR historical cohort stud*[Title/Abstract] OR concurrent stud*[Title/Abstract] OR prospective cohort[Title/Abstract] OR prospective stud*[Title/Abstract] OR prospective[Title/Abstract] OR retrospective cohort[Title/Abstract] OR retrospective stud*[Title/Abstract] OR retrospective[Title/Abstract] OR case-control[Title/Abstract] OR case control[Title/Abstract] OR case-control study case control study[Title/Abstract] OR case-referent stud*[Title/Abstract] OR case referent stud*[Title/Abstract] OR case-comparison stud*[Title/Abstract] OR case comparison stud*[Title/Abstract] OR case-base stud*[Title/Abstract] OR case base stud*[Title/Abstract] OR nested case-control stud*[Title/Abstract] OR nested case control stud*[Title/Abstract] OR matched case-control stud*[Title/Abstract] OR matched case control stud*[Title/Abstract] OR randomized clinical trial*[Title/Abstract] OR clinical trial[Title/Abstract] OR RCT*[Title/Abstract] )

**List of excluded studies**

1. Ahmad, Tessnim R.; Vasudevan, Harish N.; Lazar, Ann A.; Chan, Jason W.; George, Jonathan R.; Alvarado, Michael D.; Yu, Siegrid S.; Daud, Adil; Yom, Sue S.. Should Sentinel Lymph Node Biopsy Status Guide Adjuvant Radiation Therapy in Patients With Merkel Cell Carcinoma?. Advances in radiation oncology. Outcome
2. Allen, Peter J.; Bowne, Wilbur B.; Jaques, David P.; Brennan, Murray F.; Busam, Klaus; Coit, Daniel G.. Merkel cell carcinoma: Prognosis and treatment of patients from a single institution. Journal of Clinical Oncology. Intervention
3. Alonso, R. S.; Lahbabi, I.; Ben Hassel, M.; Boisselier, P.; Chaari, N.; Lesimple, T.; Chevrier, S.; de Crevoisier, R.. Merkel cell carcinoma: Outcome and role of radiotherapy. CANCER RADIOTHERAPIE. Study design
4. Andtback, H. B.; Bjornhagen-Safwenberg, V.; Shi, H.; Lui, W. O.; Masucci, G. V.; Villabona, L.. Sex Differences in Overall Survival and the Effect of Radiotherapy in Merkel Cell Carcinoma-A Retrospective Analysis of A Swedish Cohort. CANCERS. Outcome
5. Arron, Sarah T.; Canavan, Theresa; Yu, Siegrid S.. Organ transplant recipients with Merkel cell carcinoma have reduced progression-free, overall, and disease-specific survival independent of stage at presentation.. Journal of the American Academy of Dermatology. Intervention
6. Arroyave, Aaron J.; Lewis, James M.; Landry, Miles; McLoughlin, James M.; Enomoto, Laura M.. Merkel Cell Polyomavirus Antibody Titer Predicts Recurrence-Free Survival.. Annals of surgical oncology. Intervention
7. Asgari, Maryam M.; Sokil, Monica M.; Warton, E. Margaret; Iyer, Jayasri; Paulson, Kelly G.; Nghiem, Paul. Effect of host, tumor, diagnostic, and treatment variables on outcomes in a large cohort with Merkel cell carcinoma.. JAMA dermatology. Outcome
8. Assouline, A.; Levy, A.; Mazeron, J.-J.; Chargari, C.; Krzisch, C.. [Management of Merkel cell carcinoma: Role of radiotherapy in elderly patients].. Cancer radiotherapie : journal de la Societe francaise de radiotherapie oncologique. Study design
9. Assouline, A.; Tai, P.; Levy, A.; Yu, E.; Lian, J. D.; Joseph, K.; Miale, T.; Krzisch, C.. Management for 145 cases of Merkel cell carcinoma. BULLETIN DU CANCER. Study design
10. Bajetta, Emilio; Celio, Luigi; Platania, Marco; Lo Vullo, Salvatore; Patuzzo, Roberto; Maurichi, Andrea; Santinami, Mario. Single-institution series of early-stage Merkel cell carcinoma: long-term outcomes in 95 patients managed with surgery alone.. Annals of surgical oncology. Outcome
11. Balakrishnan, V.; Berry, S.; Stew, B.; Sizeland, A.. Benefits of combined modality treatment of Merkel cell carcinoma of the head and neck: single institution experience.. The Journal of laryngology and otology. Outcome
12. Beenken, Samuel W.; Urist, Marshall M.. Treatment options for Merkel cell carcinoma.. Journal of the National Comprehensive Cancer Network : JNCCN. Study design
13. Benessahraoui, M.; Dalstein, V.; Lorchel, F.; Algros, M.-P.; Puzenat, E.; Louvat, P.; Hassam, B.; Humbert, P.-H.; Aubin, F.. [Merkel cell carcinoma: descriptive study of 24 cases (1993-2001)].. La Revue de medecine interne. Study design
14. Bhanegaonkar, A.; Liu, F. X.; Boyd, M.; Fulcher, N.; Kim, R.; Krulewicz, S.; Smith, J.; Cowey, C. L.. Real-World Clinical Outcomes in Patients with Locally Advanced or Metastatic Merkel Cell Carcinoma Treated in U.S. Oncology Clinical Practices: Results from SPEAR-Merkel. . Comparator
15. Bichakjian, C. K.; Harms, K. L.; Schwartz, J. L.. Selective Use of Adjuvant Therapy in the Management of Merkel Cell Carcinoma. JAMA ONCOLOGY. Duplicate of No. 40
16. Bielamowiez, S.; Smith, D.; Abemayor, E.. Merkel cell carcinoma: An aggressive skin neoplasm. . Outcome
17. Bischof, M.; van Kampen, M.; Huber, P.; Wannenmacher, M.. Merkel cell carcinoma: the role of radiation therapy in general management.. Strahlentherapie und Onkologie : Organ der Deutschen Rontgengesellschaft ... [et al]. Outcome
18. Bjorn-Andtback, H.; Masucci, G. V.; Bjornhagen-Safwenberg, V.; Lui, W. O.; Villabona, L. E. E.. Prognostic differences and the effect of radiotherapy on survival in Merkel cell carcinoma: A retrospective analysis of a Swedish cohort.. JOURNAL OF CLINICAL ONCOLOGY. Outcome
19. Bleicher, J.; Asare, E. A.; Flores, S.; Bowles, T. L.; Bowen, G. M.; Hyngstrom, J. R.. Oncologic outcomes of patients with Merkel Cell Carcinoma (MCC): A multi-institutional cohort study. AMERICAN JOURNAL OF SURGERY. Outcome
20. Bloomstein, J. D.; Eisen, D. B.. Merkel cell carcinoma treatment with Mohs micrographic surgery versus wide local excision: A retrospective cohort survival analysis. . Outcome
21. Blythe, John N. St J.; Macpherson, David; Reuther, William J.; Ethunandan, Madan; Ilankovan, Velupillai; Sharma, Sanjay; Anand, Rajiv A.; Mellor, Timothy K.; Kerawala, Cyrus; Brennan, Peter A.. Management of early stage cutaneous Merkel cell carcinoma of the head and neck.. The British journal of oral & maxillofacial surgery. Intervention
22. Bowe, C. M.; Bean, T.; Loke, R.; Gallagher, N.; Rooney, J.; Surwald, C.; Dhanda, J.; Moody, A.; Bisase, B.; Norris, P.; Barrett, A. W.; Lachanas, V.; Doumas, S.. Merkel cell carcinoma of the head and neck in the south-east of England.. The British journal of oral & maxillofacial surgery. Outcome
23. Brewer, J. D.; Shanafelt, T. D.; Otley, C. C.; Roenigk, R. K.; Cerhan, J. R.; Kay, N. E.; Weaver, A. L.; Call, T. G.. Chronic lymphocytic leukemia is associated with decreased survival of patients with malignant melanoma and merkel cell carcinoma in a SEER population-based study. . Population
24. Broida, S. E.; Chen, X. T.; Baum, C. L.; Brewer, J. D.; Block, M. S.; Jakub, J. W.; Pockaj, B. A.; Foote, R. L.; Markovic, S. N.; Hieken, T. J.; Houdek, M. T.. Merkel cell carcinoma of unknown primary: Clinical presentation and outcomes. JOURNAL OF SURGICAL ONCOLOGY. Outcome
25. Bryant, M. K.; Ward, C.; Gaber, C. E.; Strassle, P. D.; Ollila, D. W.; Laks, S.. Decreased survival and increased recurrence in Merkel cell carcinoma significantly linked with immunosuppression. JOURNAL OF SURGICAL ONCOLOGY. Population
26. Butala, A. A.; Jain, V.; Reddy, V. K.; Sebro, R. A.; Song, Y.; Karakousis, G.; Mitchell, T. C.; Lukens, J. N.; Shabason, J. E.. Impact ofTumor-InfiltratingLymphocytes on Overall Survival in Merkel Cell Carcinoma. ONCOLOGIST. Outcome
27. Candrian, C.; Ruedi, Th; Furrer, M.. [Merkel cell carcinoma. Retrospective analysis of 4 cases with special reference to diagnosis, therapy and long-term outcome].. Swiss surgery = Schweizer Chirurgie = Chirurgie suisse = Chirurgia svizzera. Outcome
28. Chandra, Sunandana; Zheng, Ying; Pandya, Shivani; Yu, Ting; Kearney, Mairead; Wang, Li; Kim, Ruth; Phatak, Hemant. Real-world outcomes among US Merkel cell carcinoma patients initiating immune checkpoint inhibitors or chemotherapy.. Future oncology (London, England). Comparator
29. Chang, John Wen-Cheng; Chang, Yao-Yu; Huang, Yen-Lin; Lo, Yun-Feng; Ho, Tsung-Ying; Huang, Yi-Ting; Chen, Huan-Wu; Yeh, Chun-Nan; Wu, Chiao-En. Merkel cell carcinoma in Taiwan: A series of 24 cases and literature review.. Medicine. Intervention
30. Chen, Michelle M.; Roman, Sanziana A.; Sosa, Julie A.; Judson, Benjamin L.. The role of adjuvant therapy inthe management of head and neck merkel cell carcinoma: An analysis of 4815 patients. JAMA Otolaryngology - Head and Neck Surgery. Population
31. Chen, S.-W.; Chang, S.-T.; Ho, C.-H.; Wang, J.-S.; Wang, R. C.; Takeuchi, K.; Chuang, S.-S.. Merkel cell carcinoma in Taiwan: A rare tumour with a better prognosis in those harbouring Merkel cell polyomavirus. . Outcome
32. Cheraghlou, Shayan; Sadda, Praneeth; Agogo, George O.; Girardi, Michael. A machine-learning modified CART algorithm informs Merkel cell carcinoma prognosis.. The Australasian journal of dermatology. Intervention
33. Chipidza, Fallon E.; Thakuria, Manisha; Schoenfeld, Jonathan D.; Silk, Ann W.; Catalano, Paul J.; Yoon, Charles H.; Hanna, Glenn J.; DeCaprio, James A.; Tishler, Roy B.; Margalit, Danielle N.. Association between treatment center experience and survival after diagnosis of stage I to III Merkel cell carcinoma treated with surgery with or without postoperative radiation therapy.. JAAD. Study design
34. Chust, M.; Gaspar, C.; Hernandez, A.; Burriel, C.; Casamayor, J.; Sanmartin, O.; Arribas, L.; Lopez, J.; Guillem, V.. Merkel cell carcinoma. Experience in the treatment of 9 cases and long term follow-up . . Study design
35. Ciazynska, M.; Szczepaniak, Katarzyna; Pabianek, Marta; Nejc, Dariusz; Reich, Adam; Ulanska, M.; Owczarek, Witold; Skibinska, M.; Narbutt, Joanna; Lesiak, Aleksandra; Szczepaniak, Katarzyna; Pabianek, Marta; Nejc, Dariusz; Reich, Adam; Owczarek, Witold; Joanna; Lesiak, Aleksandra. Primary Merkel Cell Carcinoma: A Retrospective Analysis of 31 Cases in Poland. DERMATOLOGY AND THERAPY. Outcome
36. Cirillo, F.; Vismarra, M.; Cafaro, I.; Martinotti, M.. Merkel cell carcinoma: A retrospective study on 48 cases and review of literature. . Population
37. Clark, Jonathan R.; Veness, Michael J.; Gilbert, Ralph; O'Brien, Christopher J.; Gullane, Patrick J.. Merkel cell carcinoma of the head and neck: is adjuvant radiotherapy necessary?. Head & neck. Outcome
38. Collins, M. K.; Cameron, F. G.. Solitary regional bony recurrence in Merkel cell carcinoma.. Australasian radiology. Outcome
39. Conic, Rosalynn R. Z.; Ko, Jennifer; Saridakis, Stephanie; Damiani, Giovanni; Funchain, Pauline; Vidimos, Allison; Gastman, Brian R.. Sentinel lymph node biopsy in Merkel cell carcinoma: Predictors of sentinel lymph node positivity and association with overall survival.. Journal of the American Academy of Dermatology. Intervention
40. Cook, Maclean; Baker, Kelsey; Redman, Mary; Lachance, Kristina; Nguyen, Macklin H.; Parvathaneni, Upendra; Bhatia, Shailender; Nghiem, Paul; Tseng, Yolanda D.. Differential Outcomes Among Immunosuppressed Patients With Merkel Cell Carcinoma: Impact of Immunosuppression Type on Cancer-specific and Overall Survival.. American journal of clinical oncology. Outcome
41. Cook, M. M.; Schaub, S. K.; Goff, P. H.; Fu, A.; Park, S. Y.; Hippe, D. S.; Liao, J. J.; Apisarnthanarax, S.; Bhatia, S.; Tseng, Y. D.; Nghiem, P. T.; Parvathaneni, U.. Postoperative, Single-Fraction Radiation Therapy in Merkel Cell Carcinoma of the Head and Neck. ADVANCES IN RADIATION ONCOLOGY. Outcome
42. Dancey, A. L.; Rayatt, S. S.; Soon, C.; Ilchshyn, A.; Brown, I.; Srivastava, S.. Merkel cell carcinoma: a report of 34 cases and literature review.. Journal of plastic, reconstructive & aesthetic surgery : JPRAS. Outcome
43. Danino-Garcia, M.; Dominguez-Cruz, J. J.; Perez-Ruiz, C.; Conejo-Mir, J.; Pereyra-Rodriguez, J. J.; Danino-Garcia, M.; Dominguez-Cruz, J. J.; Perez-Ruiz, C.; Conejo-Mir, J.; Pereyra-Rodriguez, J. J.. Clinical and Epidemiological Characteristics of Merkel Cell Carcinoma in a Series of 38 Patients. Actas Dermo-Sifiliograficas. Outcome
44. Dasanu, Constantin A.; Del Rosario, Michael; Codreanu, Ion; Lu, Yani; Farrell, Stephanie; Hyams, David M.; Plaxe, Steven C.. Merkel cell carcinoma: long-term follow-up of a single institution series and clinical outcomes by immunological status.. Dermatology online journal. Population
45. Dawe, Nicholas; Sainsbury, David; Veeramani, Siva; Ragbir, Maniram; Ahmed, Omar A.. Merkel cell carcinoma of the head and neck: challenges in implementing best practice.. Annals of plastic surgery. Population
46. Desai, Amar D.; Behbahani, Sara; Samie, Faramarz H.. Predictors of time to definitive surgery and survival in Merkel cell carcinoma: analysis of the US National Cancer Database.. Clinical and experimental dermatology. Outcome
47. Dinges, L. A.; Eichkorn, T.; Regnery, S.; Hoerner-Rieber, J.; Debus, J.; Hassel, J. C.; Lang, K. S.. Postoperative Radiotherapy and the Role of Regional Lymph Node Irradiation in Localized Merkel Cell Carcinoma: A Single-Center Retrospective Analysis. CANCERS. Intervention
48. Dudzisz-Sledz, Monika; Sobczuk, Pawel; Kozak, Katarzyna; Switaj, Tomasz; Kosela-Paterczyk, Hanna; Czarnecka, Anna Malgorzata; Falkowski, Slawomir; Rogala, Pawel; Morysinski, Tadeusz; Spalek, Mateusz Jacek; Zdzienicki, Marcin; Goryn, Tomasz; Zietek, Marcin; Cybulska-Stopa, Bozena; Klek, Stanis{\l}aw; Kaminska-Winciorek, Grazyna; Ziolkowska, Barbara; Szumera-Cieckiewicz, Anna; Rutkowski, Piotr. Treatment of Locally Advanced Merkel Cell Carcinoma-A Multi-Center Study.. Cancers. Outcome
49. Eichorn, F. C.; Dubey, A.; Pathak, K. A.. Merkel Cell Carcinoma of the Head and Neck. PLASTIC SURGERY. Outcome
50. Ely, Haines; Pascucci, Annabella. Merkel cell carcinoma: treatment with bleomycin.. Dermatology online journal. Outcome
51. Eng, Tony Y.; Boersma, Melisa G. K.; Fuller, Clifton D.; Cavanaugh, Sean X.; Valenzuela, Fabio; Herman, Terence S.. Treatment of merkel cell carcinoma.. American journal of clinical oncology. Outcome
52. Eng, Tony Y.; Naguib, Marco; Fuller, Clifton D.; Jones, William E. 3rd; Herman, T. S.. Treatment of recurrent Merkel cell carcinoma: an analysis of 46 cases.. American journal of clinical oncology. Outcome
53. Esposito, Andrew; Jacobs, Daniel; Ariyan, Stephan; Galan, Anjela; Kluger, Harriet; Clune, James; Weiss, Sarah; Tran, Thuy; Olino, Kelly. Merkel Cell Carcinoma: Changing Practice Patterns and Impact on Recurrence-Free and Overall Survival at a Single Institution and Nationally. Annals of Surgical Oncology. Population
54. Ezaldein, Harib H.; Ventura, Alessandra; DeRuyter, Nicolaas P.; Yin, Emily S.; Giunta, Alessandro. Understanding the influence of patient demographics on disease severity, treatment strategy, and survival outcomes in merkel cell carcinoma: a surveillance, epidemiology, and end-results study.. Oncoscience. Outcome
55. Farley, C. R.; Perez, M. C.; Soelling, S. J.; Delman, K. A.; Harit, A.; Wuthrick, E. J.; Messina, J. L.; Sondak, V. K.; Zager, J. S.; Lowe, M. C.. Merkel cell carcinoma outcomes: does AJCC8 underestimate survival?. Annals of surgical oncology. Outcome
56. Fenig, E.; Brenner, B.; Katz, A.; Rakovsky, E.; Hana, M. B.; Sulkes, A.. The role of radiation therapy and chemotherapy in the treatment of Merkel cell carcinoma.. Cancer. Outcome
57. Fennig, S.; Landman, Y.; Brenner, R.; Billan, S.; Fenig, E.. Merkel cell carcinoma in lymph nodes with and without primary origin. CANCER MEDICINE. Outcome
58. Ferrandiz-Pulido, C.; Gomez-Tomas, A.; Llombart, B.; Mendoza, D.; Marcoval, J.; Piaserico, S.; Baykal, C.; Bouwes-Bavinck, J. N.; Racz, E.; Kanitakis, J.; Harwood, C. A.; Cetkovska, P.; Geusau, A.; Del Marmol, V.; Masferrer, E.; Orte Cano, C.; Ricar, J.; de Oliveira, W. R.; Salido-Vallejo, R.; Ducroux, E.; Gkini, M. A.; Lopez-Guerrero, J. A.; Kutzner, H.; Kempf, W.; Secckin, D.. Clinicopathological features, MCPyV status and outcomes of Merkel cell carcinoma in solid-organ transplant recipients: a retrospective, multicentre cohort study.. Journal of the European Academy of Dermatology and Venereology : JEADV. Population
59. Fiedler, Eckhard; Vordermark, Dirk. Outcome of Combined Treatment of Surgery and Adjuvant Radiotherapy in Merkel Cell Carcinoma.. Acta dermato-venereologica. Outcome
60. Fields, Ryan C.; Busam, Klaus J.; Chou, Joanne F.; Panageas, Katherine S.; Pulitzer, Melissa P.; Allen, Peter J.; Kraus, Dennis H.; Brady, Mary S.; Coit, Daniel G.. Five hundred patients with Merkel cell carcinoma evaluated at a single institution.. Annals of surgery. Outcome
61. Fields, Ryan C.; Busam, Klaus J.; Chou, Joanne F.; Panageas, Katherine S.; Pulitzer, Melissa P.; Kraus, Dennis H.; Brady, Mary S.; Coit, Daniel G.. Recurrence and survival in patients undergoing sentinel lymph node biopsy for merkel cell carcinoma: analysis of 153 patients from a single institution.. Annals of surgical oncology. Outcome
62. Fields, Ryan C.; Busam, Klaus J.; Chou, Joanne F.; Panageas, Katherine S.; Pulitzer, Melissa P.; Allen, Peter J.; Kraus, Dennis H.; Brady, Mary S.; Coit, Daniel G.. Recurrence after complete resection and selective use of adjuvant therapy for stage I through III Merkel cell carcinoma.. Cancer. Outcome
63. Fleming, Kirsten E.; Ly, Thai Yen; Pasternak, Sylvia; Godlewski, Marek; Doucette, Steve; Walsh, Noreen M.. Support for p63 expression as an adverse prognostic marker in Merkel cell carcinoma: report on a Canadian cohort.. Human pathology. Outcome
64. Fochtmann-Frana, A.; Haymerle, G.; Loewe, R.; Grasl, M. C.; Pammer, J.; Rath, T.; Perisanidis, C.; Erovic, B. M.. Incurable, progressive Merkel cell carcinoma: A single-institution study of 54 cases.. . Outcome
65. Foote, Matthew; Harvey, Jennifer; Porceddu, Sandro; Dickie, Graeme; Hewitt, Susan; Colquist, Shoni; Zarate, Dannie; Poulsen, Michael. Effect of radiotherapy dose and volume on relapse in Merkel cell cancer of the skin.. International journal of radiation oncology, biology, physics. Outcome
66. Foote, Matthew; Veness, Michael; Zarate, Dannie; Poulsen, Michael. Merkel cell carcinoma: the prognostic implications of an occult primary in stage IIIB (nodal) disease.. Journal of the American Academy of Dermatology. Outcome
67. Frohm, Marcus L.; Griffith, Kent A.; Harms, Kelly L.; Hayman, James A.; Fullen,
68. Douglas R.; Nelson, Christine C.; Wong, Sandra L.; Schwartz, Jennifer L.; Bichakjian, Christopher K.. Recurrence and Survival in Patients With Merkel Cell Carcinoma Undergoing Surgery Without Adjuvant Radiation Therapy to the Primary Site.. JAMA dermatology. Intervention
69. Garcia-Zamora, E.; Ganuza, M. V.; Martin-Alcalde, J.; Medrano, R. M.; Moraleda, F. P.; Lopez-Estebaranz, J. L.. Merkel Cell Carcinoma: A Description of 11 Cases. ACTAS DERMO-SIFILIOGRAFICAS. Study design
70. Ghadjar, Pirus; Kaanders, Johannes H.; Poortmans, Philipp; Zaucha, Renata; Krengli, Marco; Lagrange, Jean L.; {\{O}}zsoy. The Essential Role of Radiotherapy in the Treatment of Merkel Cell Carcinoma: A Study From the Rare Cancer Network. International Journal of Radiation Oncology*Biology*Physics. Intervention
71. Go, C. C.; Kim, D. H.; Briceno, C. A.. A SEER analysis of survival and prognostic factors in merkel cell carcinoma of the head and neck region. . Outcome
72. Goldberg, Stephanie R.; Neifeld, James P.; Frable, William J.. Prognostic value of tumor thickness in patients with Merkel cell carcinoma.. Journal of surgical oncology. Outcome
73. Gollard, R.; Weber, R.; Kosty, M. P.; Greenway, H. T.; Massullo, V.; Humberson, C.. Merkel cell carcinoma: review of 22 cases with surgical, pathologic, and therapeutic considerations.. Cancer. Outcome
74. Gonzalez-Romero, N.; Lobato-Izagirre, A.; Blanch-Rius, L.; Cancho-Galan, G.; Izu-Belloso, R.. Merkel cell carcinoma. A retrospective observational study (1998-2018) in a University Hospital . . Study design
75. Grandpeix, C.; Bonvalot, S.; Petrow, P.; Fraitag, S.; Gounod, N.; Avril, M. F.. [Continued complete remission of Merkel cell carcinoma with in-transit metastasis after treatment with isolated limb perfusion regional chemotherapy].. Annales de dermatologie et de venereologie. Study design
76. Grotz, Travis E.; Joseph, Richard W.; Pockaj, Barbara A.; Foote, Robert L.; Otley, Clark C.; Bagaria, Sanjay P.; Weaver, Amy L.; Jakub, James W.. Negative Sentinel Lymph Node Biopsy in Merkel Cell Carcinoma is Associated with a Low Risk of Same-Nodal-Basin Recurrences.. Annals of surgical oncology. Outcome
77. Gupta, Sheela G.; Wang, Linda C.; Penas, Pablo F.; Gellenthin, Martina; Lee, Stephanie J.; Nghiem, Paul. Sentinel lymph node biopsy for evaluation and treatment of patients with Merkel cell carcinoma: The Dana-Farber experience and meta-analysis of the literature.. Archives of dermatology. Outcome
78. Haerle, Stephan K.; Shiau, Carolyn; Goldstein, David P.; Qiu, Xin; Erovic, Boban M.; Ghazarian, Danny; Xu, Wei; Irish, Jonathan C.. Merkel cell carcinoma of the head and neck: potential histopathologic predictors.. The Laryngoscope. Outcome
79. Hanna, G. J.; Kacew, A. J.; Tanguturi, A. R.; Grote, H. J.; Vergara, V.; Brunkhorst, B.; Rabinowits, G.; Thakuria, M.; LeBoeuf, N. R.; Ihling, C.; DeCaprio, J. A.; Lorch, J. H.. Association of Programmed Death 1 Protein Ligand (PD-L1) Expression With Prognosis in Merkel Cell Carcinoma. FRONTIERS IN MEDICINE. Outcome
80. Harary, Maya; Kavouridis, Vasileios K.; Thakuria, Manisha; Smith, Timothy R.. Predictors of survival in neurometastatic Merkel cell carcinoma.. European journal of cancer (Oxford, England : 1990). Population
81. Harounian, J. A.; Molin, N.; Galloway, T. J.; Facs, D. R.; Bauman, J.; Farma, J.; Reddy, S.; Lango, M. N.. Effect of Sentinel Lymph Node Biopsy andLVIon Merkel Cell Carcinoma Prognosis and Treatment. LARYNGOSCOPE. Outcome
82. Harrington, Chris; Kwan, Winkle. Outcomes of Merkel cell carcinoma treated with radiotherapy without radical surgical excision.. Annals of surgical oncology. Outcome
83. Harrington, Chris; Kwan, Winkle. Radiotherapy and Conservative Surgery in the Locoregional Management of Merkel Cell Carcinoma: The British Columbia Cancer Agency Experience.. Annals of surgical oncology. Outcome
84. Haymerle, Georg; Fochtmann, Alexandra; Kunstfeld, Rainer; Pammer, Johannes; Erovic, Boban M.. Management of Merkel cell carcinoma of unknown primary origin: the Vienna Medical School experience.. European archives of oto-rhino-laryngology : official journal of the European Federation of Oto-Rhino-Laryngological Societies (EUFOS) : affiliated with the German Society for Oto-Rhino-Laryngology - Head and Neck Surgery. Outcome
85. Haymerle, Georg; Fochtmann, Alexandra; Kunstfeld, Rainer; Pammer, Johannes; Erovic, Boban M.. Merkel cell carcinoma: Overall survival after open biopsy versus wide local excision.. Head & neck. Outcome
86. Heidenreich, A.; Hulskamp, P.; Mezzadri, N.; Bugari, G.; Celeste, F.; Chirife, A. M.; Buxhoeveden, R. B.. Markel cell neuroendocrinal carcinoma . . Study design
87. K Hohaus, E Köstler, J Schönlebe, E Klemm, U Wollina. Merkel cell carcinoma--a retrospective analysis of 17 cases.. Journal of the European Academy of Dermatology and Venereology : JEADV. Outcome
88. Housman, Douglas M.; Decker, Roy H.; Wilson, Lynn D.. Regarding adjuvant radiation therapy in merkel cell carcinoma: selection bias and its affect on overall survival.. . Outcome
89. Hui, Andrew C.; Stillie, Alison L.; Seel, Matthew; Ainslie, Jill. Merkel cell carcinoma: 27-year experience at the Peter MacCallum Cancer Centre.. International journal of radiation oncology, biology, physics. Outcome
90. Hurley, C. M.; ALNafisee, D.; Jones, D.; Kelly, J. L.; Regan, P. J.; Hussey, A. J.; McInerney, N.. Head and Neck Merkel Cell Carcinoma: A 12-Year Single Institutional Experience.. JPRAS open. Outcome
91. ISRCTN16290169. Rational treatment selection for Merkel Cell Carcinoma (MCC). https://trialsearch.who.int/Trial2.aspx?TrialID=ISRCTN16290169. Study design
92. Iyer, Jayasri G.; Storer, Barry E.; Paulson, Kelly G.; Lemos, Bianca; Phillips, Jerri Linn; Bichakjian, Christopher K.; Zeitouni, Nathalie; Gershenwald, Jeffrey E.; Sondak, Vernon; Otley, Clark C.; Yu, Siegrid S.; Johnson, Timothy M.; Liegeois, Nanette J.; Byrd, David; Sober, Arthur; Nghiem, Paul. Relationships among primary tumor size, number ofÂ involved nodes, and survival for 8044 cases ofÂ Merkel cell carcinoma.. Journal of the American Academy of Dermatology. Outcome
93. Iyer, Jayasri G.; Parvathaneni, Upendra; Gooley, Ted; Miller, Natalie J.; Markowitz, Elan; Blom, Astrid; Lewis, Christopher W.; Doumani, Ryan F.; Parvathaneni, Kaushik; Anderson, Austin; Bestick, Amy; Liao, Jay; Kane, Gabrielle; Bhatia, Shailender; Paulson, Kelly; Nghiem, Paul. Single-fraction radiation therapy in patients with metastatic Merkel cell carcinoma.. Cancer medicine. Population
94. Iyer, Jayasri G.; Blom, Astrid; Doumani, Ryan; Lewis, Christopher; Tarabadkar, Erica S.; Anderson, Austin; Ma, Christine; Bestick, Amy; Parvathaneni, Upendra; Bhatia, Shailender; Nghiem, Paul. Response rates and durability of chemotherapy among 62 patients with metastatic Merkel cell carcinoma.. Cancer medicine. Population
95. Jabbour, James; Cumming, Robert; Scolyer, Richard A.; Hruby, George; Thompson, John F.; Lee, Stephen. Merkel cell carcinoma: assessing the effect of wide local excision, lymph node dissection, and radiotherapy on recurrence and survival in early-stage disease--results from a review of 82 consecutive cases diagnosed between 1992 and 2004.. Annals of surgical oncology. Outcome
96. Jackson, Philippa C.; Wallis, Katy; Allgar, Victoria; Lind, Michael J.; Stanley, Paul R. W.. Merkel cell carcinoma in East Yorkshire: A case series and literature review of current management.. Journal of plastic, reconstructive & aesthetic surgery : JPRAS. Outcome
97. Jacobs, Daniel; Olino, Kelly; Park, Henry S.; Clune, James; Cheraghlou, Shayan; Girardi, Michael; Burtness, Barbara; Kluger, Harriet; Judson, Benjamin L.. Primary Treatment Selection for Clinically Node-Negative Merkel Cell Carcinoma of the Head and Neck.. Otolaryngology--head and neck surgery : official journal of American Academy of Otolaryngology-Head and Neck Surgery. Outcome
98. Johnson, Matthew E.; Zhu, Fang; Li, Tianyu; Wu, Hong; Galloway, Thomas J.; Farma, Jeffrey M.; Perlis, Clifford S.; Turaka, Aruna. Absolute lymphocyte count: a potential prognostic factor for Merkel cell carcinoma.. Journal of the American Academy of Dermatology. Outcome
99. Kachare, Swapnil D.; Wong, Jan H.; Vohra, Nasreen A.; Zervos, Emmanuel E.; Fitzgerald, Timothy L.. Sentinel lymph node biopsy is associated with improved survival in Merkel cell carcinoma.. Annals of surgical oncology. Outcome
100. Kanagaratnam, B.; Shah, A.; Anand, G.. Palliative radiotherapy for Merkel cell carcinoma: Single-centre experience and review of the literature. . Study design
101. Kervarrec, Thibault; Gaboriaud, Pauline; Berthon, Patricia; Zaragoza, Julia; Schrama, David; Houben, Roland; Le Corre, Yannick; Hainaut-Wierzbicka, Ewa; Aubin, Francois; Bens, Guido; Domenech, Jorge; Guyetant, Serge; Touze, Antoine; Samimi, Mahtab. Merkel cell carcinomas infiltrated with CD33+ myeloid cells and CD8+ T cells are associated with improved outcome. Journal of the American Academy of Dermatology. Outcome
102. Kervarrec, Thibault; Zaragoza, Julia; Gaboriaud, Pauline; Le Gouge, Amelie; Beby-Defaux, Agnes; Le Corre, Yannick; Hainaut-Wierzbicka, Ewa; Aubin, Francois; Bens, Guido; Michenet, Patrick; Maillard, Herve; Touze, Antoine; Samimi, Mahtab; Guyetant, Serge. Differentiating Merkel cell carcinoma of lymph nodes without a detectable primary skin tumor from other metastatic neuroendocrine carcinomas: The ELECTHIP criteria.. Journal of the American Academy of Dermatology. Outcome
103. Kirchberger, M. C.; Heppt, M. V.; Schuler, G.; Berking, C.; Heinzerling, L.. Merkel Cell Carcinoma of the Head and Neck Compared to Other Anatomical Sites in a Real-World Setting: Importance of Surgical Therapy for Facial Tumors. FACIAL PLASTIC SURGERY. Outcome
104. Kline, Laura; Coldiron, Brett. Mohs Micrographic Surgery for the Treatment of Merkel Cell Carcinoma.. Dermatologic surgery : official publication for American Society for Dermatologic Surgery [et al.]. Outcome
105. Knopf, Andreas; Bas, Murat; Hofauer, Benedikt; Mansour, Naglaa; Stark, Thomas. Clinicopathological characteristics of head and neck Merkel cell carcinomas.. Head & neck. Outcome
106. Koh, C. S. L.; Veness, M. J.. Role of definitive radiotherapy in treating patients with inoperable Merkel cell carcinoma: The Westmead Hospital experience and a review of the literature. AUSTRALASIAN JOURNAL OF DERMATOLOGY.
107. Kokoska, E. R.; Kokoska, M. S.; Collins, B. T.; Stapleton, D. R.; Wade, T. P.. Early aggressive treatment for Merkel cell carcinoma improves outcome.. American journal of surgery. Outcome
108. V Koljonen , T Böhling , G Granhroth c, E Tukiainen . Merkel cell carcinoma: a clinicopathological study of 34 patients.. European journal of surgical oncology : the journal of the European Society of Surgical Oncology and the British Association of Surgical Oncology. Outcome
109. Kotteas, E. A.; Pavlidis, N.. Neuroendocrine Merkel cell nodal carcinoma of unknown primary site: Management and outcomes of a rare entity. . Outcome
110. Kwan, Kera; Ghazizadeh, Shabnam; Moon, Andy S.; Runger. Merkel Cell Carcinoma: A 28-Year Experience.. Otolaryngology--head and neck surgery : official journal of American Academy of Otolaryngology-Head and Neck Surgery. Population
111. Lawenda, B. D.; Thiringer, J. K.; Foss, R. D.; Johnstone, P. A.. Merkel cell carcinoma arising in the head and neck: optimizing therapy.. American journal of clinical oncology.
112. Lemos, Bianca D.; Storer, Barry E.; Iyer, Jayasri G.; Phillips, Jerri Linn; Bichakjian, Christopher K.; Fang, L. Christine; Johnson, Timothy M.; Liegeois-Kwon, Nanette J.; Otley, Clark C.; Paulson, Kelly G.; Ross, Merrick I.; Yu, Siegrid S.; Zeitouni, Nathalie C.; Byrd, David R.; Sondak, Vernon K.; Gershenwald, Jeffrey E.; Sober, Arthur J.; Nghiem, Paul. Pathologic nodal evaluation improves prognostic accuracy in Merkel cell carcinoma: analysis of 5823 cases as the basis of the first consensus staging system.. Journal of the American Academy of Dermatology. Outcome
113. Liao, Wen-Chieh; Keng, Chen; Ma, Hsu; Hsu, Chih-Yi. Primary Merkel Cell Carcinoma: The Clinical Experience of Taipei Veterans General Hospital Revisited.. Annals of plastic surgery. Outcome
114. Linjawi, A.; Jamison, W. B.; Meterissian, S.. Merkel cell carcinoma: important aspects of diagnosis and management.. The American surgeon. Outcome
115. Liu, Kevin X.; Milligan, Michael G.; Schoenfeld, Jonathan D.; Tishler, Roy B.; Ng, Andrea K.; Devlin, Phillip M.; Fite, Elliott; Rabinowits, Guilherme; Hanna, Glenn J.; Silk, Ann W.; Yoon, Charles H.; Thakuria, Manisha; Margalit, Danielle N.. Characterization of clinical outcomes after shorter course hypofractionated and standard-course radiotherapy for stage I-III curatively-treated Merkel cell carcinoma.. Radiotherapy and oncology : journal of the European Society for Therapeutic Radiology and Oncology. Outcome
116. Lonardo, M. T.; Marone, U.; Apice, G.; Ferrara, E.; De Chiara, A.; Cerra, R.; Chiofalo, M. G.; Mozzillo, N.. Merkel cell carcinoma: experience of 14 cases and literature review.. Journal of experimental & clinical cancer research : CR. Outcome
117. Lopez Prior, V.; Llombart Cussac, B.. The role of 18F-FDG PET/CT in the management of Merkel cell carcinoma: The experience of 51 studies in our institution . . Outcome
118. Louafi, A.; Chaussard, H.; Binder, J.-P.; Revol, M.; Servant, J.-M.. [Merkel cell carcinoma: study of 24 cases and review of the literature].. Annales de chirurgie plastique et esthetique. Outcome
119. Mahajan, Sonia; Barker, Christopher A.; Mauguen, Audrey; D'Angelo, Sandra P.; Yeh, Randy; Pandit-Taskar, Neeta. (18)F-FDG PET/CT for Posttreatment Surveillance Imaging of Patients with Stage III Merkel Cell Carcinoma.. Journal of nuclear medicine : official publication, Society of Nuclear Medicine. Outcome
120. Matkowski, Rafal; Lata, Ewelina; Zietek, Marcin; Halon, Agnieszka; Forgacz, Jozef; Szynglarewicz, Bartlomiej. Multidisciplinary management in Merkel cell carcinoma.. The Journal of dermatological treatment. Outcome
121. Matsushita, E.; Hayashi, N.; Fukushima, A.; Ueno, H.. Evaluation of treatment and prognosis of Merkel cell carcinoma of the eyelid in Japan. . Outcome
122. Mattavelli, I.; Patuzzo, R.; Torri, V.; Gallino, G.; Maurichi, A.; Lamera, M.; Valeri, B.; Bolzonaro, E.; Barbieri, C.; Tolomio, E.; Moglia, D.; Nespoli, A. M.; Galeone, C.; Saw, R.; Santinami, M.. Prognostic factors in Merkel cell carcinoma patients undergoing sentinel node biopsy.. European journal of surgical oncology : the journal of the European Society of Surgical Oncology and the British Association of Surgical Oncology. Outcome
123. Maza, S.; Trefzer, U.; Hofmann, M.; Schneider, S.; Voit, C.; Krossin, T.; Zander, A.; Audring, H.; Sterry, W.; Munz, D. L.. Impact of sentinel lymph node biopsy in patients with Merkel cell carcinoma: results of a prospective study and review of the literature. EUROPEAN JOURNAL OF NUCLEAR MEDICINE AND MOLECULAR IMAGING. Outcome
124. McAfee, W. J.; Morris, C. G.; Mendenhall, C. M.; Werning, J. W.; Mendenhall, N. P.; Mendenhall, W. M.. Merkel cell carcinoma - Treatment and outcomes. CANCER. Outcome
125. Medina-Franco, H.; Urist, M. M.; Fiveash, J.; Heslin, M. J.; Bland, K. I.; Beenken, S. W.. Multimodality treatment of Merkel cell carcinoma: case series and literature review of 1024 cases.. Annals of surgical oncology. Outcome
126. Mendenhall, William M.; Kirwan, Jessica M.; Morris, Christopher G.; Amdur, Robert J.; Werning, John W.; Mendenhall, Nancy P.. Cutaneous Merkel cell carcinoma.. American journal of otolaryngology. Outcome
127. Morand, G. B.; Madana, J.; Da Silva, S. D.; Hier, M. P.; Mlynarek, A. M.; Black, M. J.. Merkel cell carcinoma of the head and neck: poorer prognosis than non-head and neck sites.. The Journal of laryngology and otology. Outcome
128. Muller, A.; Keus, Ronald; Neumann, Norbert; Lammering, Guido; Schnabel, Thomas; Muller. Management of Merkel cell carcinoma: Case series of 36 patients. ONCOLOGY REPORTS. Population
129. Nathu, R. M.; Mendenhall, W. M.; Parsons, J. T.. Merkel cell carcinoma of the skin.. Radiation oncology investigations. Outcome
130. Nguyen, My-Lien; Mohammad-Zadeh, Ana; Krempl, Greg; Razaq, Mohammad; Collins, Lindsey; Zahoor, Talal; Zhao, Daniel; Henson, Christina. Merkel cell carcinoma: treatment and outcomes over a 10-year period at a high-volume academic center.. International journal of dermatology. Population
131. Nguyen, Brian J.; Meer, Elana A.; Bautista, Sana A.; Kim, Diana H.; Etzkorn, Jeremy R.; McGeehan, Brendan; Miller, Christopher J.; Briceno, Cesar A.. Mohs Micrographic Surgery for Facial Merkel Cell Carcinoma.. Journal of cutaneous medicine and surgery. Outcome
132. O'Connor, W. J.; Roenigk, R. K.; Brodland, D. G.. Merkel cell carcinoma. Comparison of Mohs micrographic surgery and wide excision in eighty-six patients.. Dermatologic surgery : official publication for American Society for Dermatologic Surgery [et al.]. Outcome
133. Ott, M. J.; Tanabe, K. K.; Gadd, M. A.; Stark, P.; Smith, B. L.; Finkelstein, D. M.; Souba, W. W.. Multimodality management of Merkel cell carcinoma.. Archives of surgery (Chicago, Ill. : 1960). Outcome
134. Patel, M.; Newlands, C.; Whitaker, S.. Single-centre experience of primary cutaneous Merkel cell carcinoma of the head and neck between 1996 and 2014. . Outcome
135. Patel, Sagar A.; Qureshi, Muhammad M.; Mak, Kimberley S.; Sahni, Debjani; Giacalone, Nicholas J.; Ezzat, Waleed; Jalisi, Scharukh; Truong, Minh Tam. Impact of total radiotherapy dose on survival for head and neck Merkel cell carcinoma after resection.. Head & neck. Outcome
136. Patel, Sagar A.; Qureshi, Muhammad M.; Sahni, Debjani; Truong, Minh Tam. Identifying an Optimal Adjuvant Radiotherapy Dose for Extremity and Trunk Merkel Cell Carcinoma Following Resection: An Analysis of the National Cancer Database.. JAMA dermatology. Outcome
137. Paulson, Kelly G.; Iyer, Jayasri G.; Blom, Astrid; Warton, E. Margaret; Sokil, Monica; Yelistratova, Lola; Schuman, Louise; Nagase, Kotaro; Bhatia, Shailender; Asgari, Maryam M.; Nghiem, Paul. Systemic immune suppression predicts diminished Merkel cell carcinoma-specific survival independent of stage.. The Journal of investigative dermatology. Outcome
138. Paulson, Kelly G.; Iyer, Jayasri G.; Simonson, William T.; Blom, Astrid; Thibodeau, Renee M.; Schmidt, Miranda; Pietromonaco, Stephanie; Sokil, Monica; Warton, E. Margaret; Asgari, Maryam M.; Nghiem, Paul. CD8+ lymphocyte intratumoral infiltration as a stage-independent predictor of Merkel cell carcinoma survival: a population-based study.. American journal of clinical pathology. Outcome
139. Paulson, Kelly G.; Lewis, Christopher W.; Redman, Mary W.; Simonson, William T.; Lisberg, Aaron; Ritter, Deborah; Morishima, Chihiro; Hutchinson, Kathleen; Mudgistratova, Lola; Blom, Astrid; Iyer, Jayasri; Moshiri, Ata S.; Tarabadkar, Erica S.; Carter, Joseph J.; Bhatia, Shailender; Kawasumi, Masaoki; Galloway, Denise A.; Wener, Mark H.; Nghiem, Paul. Viral oncoprotein antibodies as a marker for recurrence of Merkel cell carcinoma: A prospective validation study. Cancer. Outcome
140. Pectasides, D.; Papaxoinis, G.; Pectasides, E.; Galani, H.; Razi, E.; Katodrytis, N.; Fountzilas, G.; Economopoulos, T.. Merkel cell carcinoma of the skin: A retrospective study of 24 cases by the Hellenic Cooperative Oncology Group. Oncology. Outcome
141. Penicaud, M.; Cammilleri, S.; Giorgi, R.; Taieb. [Prognostic value of sentinel lymph node in Merkel cell carcinoma of the head and neck].. Revue de laryngologie - otologie - rhinologie. Study design
142. Perez, Matthew C.; de Pinho, Felipe R.; Holstein, Amanda; Oliver, Daniel E.; Naqvi, Syeda M. H.; Kim, Youngchul; Messina, Jane L.; Burke, Erin; Gonzalez, Ricardo J.; Sarnaik, Amod A.; Cruse, C. Wayne; Wuthrick, Evan J.; Harrison, Louis B.; Sondak, Vernon K.; Zager, Jonathan S.. Resection Margins in Merkel Cell Carcinoma: Is a 1-cm Margin Wide Enough?. Annals of surgical oncology. Outcome
143. Pergolizzi, J. Jr; Sardi, A.; Pelczar, M.; Conaway, G. L.. Merkel cell carcinoma: an aggressive malignancy.. The American surgeon. Study design
144. Peters, G. B. 3rd; Meyer, D. R.; Shields, J. A.; Custer, P. L.; Rubin, P. A.; Wojno, T. H.; Bersani, T. A.; Tanenbaum, M.; Peters III, G. B.; Meyer, D. R.; Shields, J. A.; Custer, P. L.; Rubin, P. A.; Wojno, T. H.; Bersani, T. A.; Tanenbaum, M.. Management and prognosis of Merkel cell carcinoma of the eyelid. Ophthalmology. Outcome
145. Poulsen, M.; Rischin, D.; Walpole, E.; Harvey, J.; Macintosh, J.; Ainslie, J.; Hamilton, C.; Keller, J.; Tripcony, L.. Analysis of toxicity of Merkel cell carcinoma of the skin treated with synchronous carboplatin/etoposide and radiation: a Trans-Tasman Radiation Oncology Group study.. International journal of radiation oncology, biology, physics. Outcome
146. Poulsen, Michael; Rischin, Danny; Walpole, Euan; Harvey, Jennifer; Mackintosh, John; Ainslie, Jill; Hamilton, Chris; Keller, Jacqui; Tripcony, Lee. High-risk Merkel cell carcinoma of the skin treated with synchronous carboplatin/etoposide and radiation: a Trans-Tasman Radiation Oncology Group Study--TROG 96:07.. Journal of clinical oncology : official journal of the American Society of Clinical Oncology. Outcome
147. Poulsen, Michael; Round, Caroline; Keller, Jacqui; Tripcony, Lee; Veness, Michael. Factors influencing relapse-free survival in Merkel cell carcinoma of the lower limb--a review of 60 cases.. International journal of radiation oncology, biology, physics. Population
148. Poulsen, Michael; Macfarlane, David; Veness, Michael; Estall, Venessa; Hruby, George; Kumar, Mahesh; Pullar, Andrew; Tripcony, Lee; Rischin, Danny. Prospective analysis of the utility of 18-FDG PET in Merkel cell carcinoma of the skin: A Trans Tasman Radiation Oncology Group Study, TROG 09:03.. Journal of medical imaging and radiation oncology. Outcome
149. Rabinowits, Guilherme; Lezcano, Cecilia; Catalano, Paul J.; McHugh, Patricia; Becker, Hailey; Reilly, Megan M.; Huang, Julian; Tyagi, Ayushi; Thakuria, Manisha; Bresler, Scott C.; Sholl, Lynette M.; Shapiro, Geoffrey I.; Haddad, Robert; DeCaprio, James A.. Cabozantinib in Patients with Advanced Merkel Cell Carcinoma.. The oncologist. Population
150. Rattani, A.; Gaskins, J.; McKenzie, G.; Scharf, V. K.; Broman, K.; Pisu, M.; Holder, A.; Dunlap, N.; Schwartz, D.; Yusuf, M. B.. Patterns of Care and Data Quality in a National Registry of Black and White Patients with Merkel Cell Carcinoma. CANCERS. Population
151. Redmond, J. 3rd; Perry, J.; Sowray, P.; Vukelja, S. J.; Dawson, N.. Chemotherapy of disseminated Merkel-cell carcinoma.. American journal of clinical oncology. Population
152. Ren, Kehui; Yin, Xufeng; Zhou, Bingrong. Effects of surgery on survival of patients aged 75 years or older with Merkel cell carcinoma.. Cancer medicine. Outcome
153. Ricard, A.-S.; Sessiecq, Q.; Siberchicot, F.; Jouary, T.; Laurentjoye, M.. Sentinel lymph node biopsy for head and neck Merkel cell carcinoma: a preliminary study.. European annals of otorhinolaryngology, head and neck diseases. Outcome
154. Ricci, Costantino; Morandi, Luca; Ambrosi, Francesca; Righi, Alberto; Gibertoni, Dino; Maletta, Francesca; Agostinelli, Claudio; Corradini, Angelo Gianluca; Uccella, Silvia; Asioli, Silvia; Sessa, Fausto; La Rosa, Stefano; Papotti, Mauro Giulio; Asioli, Sofia. Intron 4-5 hTERT DNA Hypermethylation in Merkel Cell Carcinoma: Frequency, Association with Other Clinico-pathological Features and Prognostic Relevance.. Endocrine pathology. Outcome
155. Richter, I.; Jirasek, T.; Cermakova, E.; Bartos, J.; Buchler. Expression and prognostic significance of programmed deathligand 1 (PD-L1) in Merkel cell carcinoma. Jbuon. Outcome
156. Ríos-Martín JJ, Rodriguez-Salas N, Vázquez-Doval FJ, Llombart B, Rojas-Ferrer N, González-Vela MC, Zulueta T, Monteagudo C, Aneiros-Fernández J, Beato MJ, Carrillo R. . Clinical and pathological features of Merkel cell carcinoma: A 4-year follow-up observational retrospective study in Spain.. Cancer epidemiology. Outcome
157. Sahi, H.; Their, J.; Gissler, M.; Koljonen, V.. Merkel Cell Carcinoma Treatment in Finland in 1986-2016-A Real-World Data Study. CANCERS. Population
158. Saito, Akira; Tsutsumida, Arata; Furukawa, Hiroshi; Saito, Noriko; Mol, William; Sekido, Mitsuru; Sasaki, Satoru; Oashi, Kohei; Kimura, Chu; Yamamoto, Yuhei. Merkel cell carcinoma of the face: an analysis of 16 cases in the Japanese.. Journal of plastic, reconstructive & aesthetic surgery : JPRAS. Population
159. Samlowski, W. E.; Moon, J.; Tuthill, R. J.; Heinrich, M. C.; Balzer-Haas, N. S.; Merl, S. A.; DeConti, R. C.; Thompson, J. A.; Witter, M. T.; Flaherty, L. E.; Sondak, V. K.. A Phase II Trial of Imatinib Mesylate in Merkel Cell Carcinoma (Neuroendocrine Carcinoma of the Skin) A Southwest Oncology Group Study (S0331). AMERICAN JOURNAL OF CLINICAL ONCOLOGY-CANCER CLINICAL TRIALS. Population
160. Sandel, Henry D. 4th; Day, Terry; Richardson, Mary S.; Scarlett, Matthew; Gutman, Katharine A.; Sandel IV, H. D.; Day, Terry; Richardson, Mary S.; Scarlett, Matthew; Gutman, Katharine A.. Merkel cell carcinoma: Does tumor size or depth of invasion correlate with recurrence, metastasis, or patient survival?. Laryngoscope. Outcome
161. Sattler, Elke; Geimer, Till; Sick, Isabell; Flaig, Michael J.; Ruzicka, Thomas; Berking, Carola; Kunte, Christian. Sentinel lymph node in Merkel cell carcinoma: to biopsy or not to biopsy?. The Journal of dermatology. Outcome
162. Savage, P.; Constenla, D.; Fisher, C.; Thomas, J. M.; Gore, M. E.. The natural history and management of Merkel cell carcinoma of the skin: a review of 22 patients treated at the Royal Marsden Hospital.. Clinical oncology (Royal College of Radiologists (Great Britain)). Population
163. Schmerling, Rafael A.; Casas, Jose G.; Cinat, Gabriela; Ospina, Fabio Ernesto Grosso; Kassuga, Luiza E. B. P.; Tlahuel, Jorge Luis Martinez; Mazzuoccolo, Luis Daniel. Burden of disease, early diagnosis, and treatment of Merkel cell carcinoma in Latin America. Journal of Global Oncology. Study design
164. Senchenkov, Alex; Barnes, Sunni A.; Moran, Steven L.. Predictors of survival and recurrence in the surgical treatment of merkel cell carcinoma of the extremities.. Journal of surgical oncology. Outcome
165. Shah, M. H.; Varker, K. A.; Collamore, M.; Zwiebel, J. A.; Coit, D.; Kelsen, D.; Chung, K. Y.. G3139 (Genasense) in patients with advanced merkel cell carcinoma. . Outcome
166. Shaikh, W. R.; Sobanko, J. F.; Etzkorn, J. R.; Shin, T. M.; Miller, C. J.. Utilization patterns and survival outcomes after wide local excision or Mohs micrographic surgery for Merkel cell carcinoma in the United States, 2004-2009. . Outcome
167. Shannon, A. B.; Straker III, R. J.; Carr, M. J.; Sun, J.; Landa, K.; Baecher, K.; Lynch, K.; Bartels, H. G.; Panchaud, R.; Keele, L. J.; Lowe, M. C.; Slingluff, C. L.; Jameson, M. J.; Tsai, K. Y.; Faries, M. B.; Beasley, G. M.; Sondak, V. K.; Karakousis, G. C.; Zager, J. S.; Miura, J. T.. An Internally Validated Prognostic Risk-Score Model for Disease-Specific Survival in Clinical Stage I and II Merkel Cell Carcinoma. . Outcome
168. Shnayder, Yelizaveta; Weed, Donald T.; Arnold, David J.; Gomez-Fernandez, Carmen; Bared, Anthony; Goodwin, W. Jarrard; Civantos, Francisco J.. Management of the neck in Merkel cell carcinoma of the head and neck: University of Miami experience.. Head & neck. Outcome
169. Sims, John R.; Grotz, Travis E.; Pockaj, Barbara A.; Joseph, Richard W.; Foote, Robert L.; Otley, Clark C.; Weaver, Amy L.; Jakub, James W.; Price, Daniel L.. Sentinel lymph node biopsy in Merkel cell carcinoma: The Mayo Clinic experience of 150 patients.. Surgical oncology. Outcome
170. Smith, Valerie A.; MaDan, Olivia P.; Lentsch, Eric J.. Tumor location is an independent prognostic factor in head and neck Merkel cell carcinoma.. Otolaryngology--head and neck surgery : official journal of American Academy of Otolaryngology-Head and Neck Surgery. Outcome
171. Smith, Franz O.; Yue, Binglin; Marzban, Suroosh S.; Walls, Brooke L.; Carr, Michael; Jackson, Ryan S.; Puleo, Christopher A.; Padhya, Tapan; Cruse, C. Wayne; Gonzalez, Ricardo J.; Sarnaik, Amod A.; Schell, Michael J.; DeConti, Ronald C.; Messina, Jane L.; Sondak, Vernon K.; Zager, Jonathan S.. Both tumor depth and diameter are predictive of sentinel lymph node status and survival in Merkel cell carcinoma.. Cancer. Outcome
172. Soltani, Ali M.; Allan, Bassan J.; Best, Matthew J.; Panthaki, Zubin J.; Thaller, Seth R.. Merkel cell carcinoma of the hand and upper extremity: current trends and outcomes.. Journal of plastic, reconstructive & aesthetic surgery : JPRAS. Population
173. Song, Y.; Zheng, C.; Shannon, A. B.; Fraker, D. L.; Miura, J. T.; Karakousis, G. C.. Sentinel lymph node positivity and overall survival in immunosuppressed patients with Merkel cell carcinoma: a national cohort study.. British Journal of Dermatology.. Outcome
174. Soult, Michael C.; Feliberti, Eric C.; Silverberg, Marc L.; Perry, Roger R.. Merkel cell carcinoma: high recurrence rate despite aggressive treatment.. The Journal of surgical research. Population
175. Sridharan, Vishwajith; Muralidhar, Vinayak; Margalit, Danielle N.; Tishler, Roy B.; DeCaprio, James A.; Thakuria, Manisha; Rabinowits, Guilherme; Schoenfeld, Jonathan D.. Merkel Cell Carcinoma: A Population Analysis on Survival.. Journal of the National Comprehensive Cancer Network : JNCCN. Population
176. Stokes, Jayme B.; Graw, Katherine S.; Dengel, Lynn T.; Swenson, Brian R.; Bauer, Todd W.; Slingluff, Craig L. Jr; Ledesma, Elihu J.. Patients with Merkel cell carcinoma tumors < or = 1.0 cm in diameter are unlikely to harbor regional lymph node metastasis.. Journal of clinical oncology : official journal of the American Society of Clinical Oncology. Population
177. Sundaresan, P.; Hruby, G.; Hamilton, A.; Hong, A.; Boyer, M.; Chatfield, M.; Thompson, J. F.. Definitive radiotherapy or chemoradiotherapy in the treatment of Merkel cell carcinoma.. Clinical oncology (Royal College of Radiologists (Great Britain)). Outcome
178. Tai, P. T.; Yu, E.; Winquist, E.; Hammond, A.; Stitt, L.; Tonita, J.; Gilchrist, J.. Chemotherapy in neuroendocrine/Merkel cell carcinoma of the skin: case series and review of 204 cases.. Journal of clinical oncology : official journal of the American Society of Clinical Oncology. Outcome
179. Tai, P. T.; Yu, E.; Tonita, J.; Gilchrist, J.. Merkel cell carcinoma of the skin.. Journal of cutaneous medicine and surgery. Population
180. Tai, Patricia; Yu, Edward; Assouline, Avi; Lian, Ji Dong; Joseph, Kurian; Miale, Thomas; Krzisch, Claude. Multimodality management for 145 cases of Merkel cell carcinoma.. Medical oncology (Northwood, London, England). Outcome
181. Tarantola, Tina I.; Vallow, Laura A.; Halyard, Michele Y.; Weenig, Roger H.; Warschaw, Karen E.; Weaver, Amy L.; Roenigk, Randall K.; Brewer, Jerry D.; Otley, Clark C.. Unknown primary Merkel cell carcinoma: 23 new cases and a review.. Journal of the American Academy of Dermatology. Population
182. Tarantola, Tina I.; Vallow, Laura A.; Halyard, Michele Y.; Weenig, Roger H.; Warschaw, Karen E.; Grotz, Travis E.; Jakub, James W.; Roenigk, Randall K.; Brewer, Jerry D.; Weaver, Amy L.; Otley, Clark C.. Prognostic factors in Merkel cell carcinoma: analysis of 240 cases.. Journal of the American Academy of Dermatology. Outcome
183. Tennvall J, Biörklund A, Johansson L, Akerman M. . Merkel cell carcinoma: management of primary, recurrent and metastatic disease. A clinicopathological study of 17 patients.. European journal of surgical oncology : the journal of the European Society of Surgical Oncology and the British Association of Surgical Oncology. Outcome
184. Terushkin, Vitaly; Brodland, David G.; Sharon, Danny J.; Zitelli, John A.. Mohs surgery for early-stage Merkel cell carcinoma (MCC) achieves local control better than wide local excision Â± radiation therapy with no increase in MCC-specific death.. International journal of dermatology. Outcome
185. Timmer, Ferdinand C. A.; Klop, W. M. C.; Relyveld, Germaine N.; Crijns, Marianne B.; Balm, A. J. M.; van den Brekel, Michiel W. M.; Lohuis, Peter J. F. M.. Merkel cell carcinoma of the head and neck: emphasizing the risk of undertreatment.. European archives of oto-rhino-laryngology : official journal of the European Federation of Oto-Rhino-Laryngological Societies (EUFOS) : affiliated with the German Society for Oto-Rhino-Laryngology - Head and Neck Surgery. Outcome
186. Trofymenko, Oleksandr; Zeitouni, Nathalie C.; Kurtzman, Drew J. B.. Factors associated with advanced-stage Merkel cell carcinoma at initial diagnosis and the use of radiation therapy: Results from the National Cancer Database.. Journal of the American Academy of Dermatology. Population
187. Tseng, Yolanda D.; Nguyen, Macklin H.; Baker, Kelsey; Cook, Maclean; Redman, Mary; Lachance, Kristina; Bhatia, Shailender; Liao, Jay J.; Apisarnthanarax, Smith; Nghiem, Paul T.; Parvathaneni, Upendra. Effect of Patient Immune Status on the Efficacy of Radiation Therapy and Recurrence-Free Survival Among 805 Patients With Merkel Cell Carcinoma.. International journal of radiation oncology, biology, physics. Outcome
188. Uitentuis, Sanne E.; Louwman, Marieke W. J.; van Akkooi, Alexander C. J.; Bekkenk, Marcel W.. Treatment and survival of Merkel cell carcinoma since 1993: A population-based cohort study in The Netherlands.. Journal of the American Academy of Dermatology. Population
189. van Veenendaal, L. M.; Madu, M. F.; Tesselaar, M. E. T.; Verhoef, C.; Grunhagen, D. J.; van Akkooi, A. C. J.. Efficacy of isolated limb perfusion (ILP) in patients with Merkel cell carcinoma (MCC): A multicenter experience. EJSO. Outcome
190. van Veenendaal, Linde M.; van Akkooi, Alexander C. J.; Verhoef, Cees; Grunhagen. Merkel cell carcinoma: Clinical outcome and prognostic factors in 351 patients.. Journal of surgical oncology. Population
191. Vayntraub, Aleksander; Tayeb, Nadine; Squires, Bryan; Mehnert, Janice M.; Hassan Ii, Quais; Sebastian, Nikhil T.; Deryaniyagala, Rohan; Quinn, Thomas J.. The Association of Radiation Therapy and Chemotherapy on Overall Survival in Merkel Cell Carcinoma: A Population-Based Analysis.. Cureus. Population
192. Victor, N. S.; Morton, B.; Smith, J. W.. Merkel cell cancer: Is Prophylactic lymph node dissection indicated?. AMERICAN SURGEON. Outcome
193. Viola, G.; Visca, P.; Bucher, S.; Migliano, E.; Lopez, M.. [Merkel cell carcinoma].. La Clinica terapeutica. Outcome
194. von der Grun, J.; Winkelmann, Ria; Meissner, Markus; Wieland, Ulrike; Silling, Steffi; Martin, Daniel; Fokas, Emmanouil; Rodel, C.; Rodel, F.; Balermpas, Panagiotis; von der Grun. Merkel Cell Polyoma Viral Load and Intratumoral CD8+ Lymphocyte Infiltration Predict Overall Survival in Patients With Merkel Cell Carcinoma. Frontiers in Oncology. Outcome
195. Voog, E.; Biron, P.; Martin, J. P.; Blay, J. Y.. Chemotherapy for patients with locally advanced or metastatic Merkel cell carcinoma. CANCER. Population
196. Wilder, R. B.; Harari, P. M.; Graham, A. R.; Shimm, D. S.; Cassady, J. R.. Merkel cell carcinoma. Improved locoregional control with postoperative radiation therapy.. Cancer. Population
197. Wilson, L. D.; Housman, D.; Smith, B. D.; Veness, M. J.. Merkel cell carcinoma: Improved outcome with the addition of adjuvant therapy . . Outcome
198. Wong, K. C.; Zuletta, F.; Clarke, S. J.; Kennedy, P. J.. Clinical management and treatment outcomes of Merkel cell carcinoma.. The Australian and New Zealand journal of surgery. Outcome
199. Yaghi, Marita; Benedetto, Paul; Greskovich, John; Haber, Roger; Dominguez, Barbara; Liang, Hong; Nahleh, Zeina; Arteta-Bulos, Rafael. Merkel cell carcinoma: Epidemiology, disease presentation, and current clinical practice outcomes.. JAAD international. Population
200. Yan, Lu; Sun, Ledong; Guan, Zhiguang; Wei, Shanshan; Wang, Yanru; Li, Pengfei. Analysis of cutaneous Merkel cell carcinoma outcomes after different surgical interventions.. Journal of the American Academy of Dermatology. Population
201. Yildiz F, Büyüksimsek M, Sakalar T, Aydin O, Turna H, Babacan NA, Yildiz B, Arslan C, Özdemir N, Urun Y, Uysal M. . Merkel cell carcinoma in Turkey: A multicentric study. Journal of cancer research and therapeutics. Population
202. Yin, X.; She, H.; Martin, L.; Carrero, K.; Ma, W.; Zhou, B.. Nomogram prediction for the overall survival and cancer-specific survival of patients diagnosed with Merkel cell carcinoma. Annals of translational medicine. Population
203. Yusuf, Mehran; Gaskins, Jeremy; Tennant, Paul; Bumpous, Jeffrey; Dunlap, Neal. Survival Impact of Time to Initiation of Adjuvant Radiation for Merkel Cell Carcinoma: An Analysis of the National Cancer Database.. Practical radiation oncology. Outcome
204. Yusuf, M.; Gaskins, J.; Wall, W.; Tennant, P.; Bumpous, J.; Dunlap, N.. Optimal adjuvant radiotherapy dose for stage I, II or III Merkel cell carcinoma: An analysis of the National Cancer Database. . Outcome
205. Yusuf, M.; Gaskins, J.; May, M. E.; Mandish, S.; Wall, W.; Fisher, W.; Tennant, P.; Jorgensen, J.; Bumpous, J.; Dunlap, N.. Immune status and the efficacy of adjuvant radiotherapy for patients with localized Merkel cell carcinoma of the head and neck.. Clinical & translational oncology : official publication of the Federation of Spanish Oncology Societies and of the National Cancer Institute of Mexico. Outcome
206. Yusuf, Mehran B.; Gaskins, Jeremy; Rattani, Abbas; McKenzie, Grant; Mandish, Steven; Wall, Weston; Farley, Alyssa; Tennant, Paul; Bumpous, Jeffrey; Dunlap, Neal. Immune Status in Merkel Cell Carcinoma: Relationships With Clinical Factors and Independent Prognostic Value.. Annals of surgical oncology. Outcome
