# Supplementary material for: Effectiveness and Safety of Treatments for Early‐Stage Merkel Cell Carcinoma: A Systematic Review and Meta‐Analysis of Randomized and Non‐Randomized Studies
Source: Cancer Med. 2025 Jan 3;14(1):e70553. doi: 10.1002/cam4.70553 (PMC11696246; doi:10.1002/cam4.70553)
Supplement: Supplementary file 3 — Appendix S3. [file CAM4-14-e70553-s004.docx]

| **Authors** | | **No of Participants** | | **Age** | | **Sex - Male (%)** | | **Clinically node positive (N)** | | **Immunosuppressed (N)** | **SNLB % (positive)** | | **Tumor location (Head and neck)** | | **Tumor location (Upper Extremities)** | | **Tumor location (Lower Extremities)** | | **Median Tumor (mm)** | **Surgery -only (N)** | | **aRT (N)** | | **Median follow-up (months)** |
| --- | --- | --- | --- | --- | --- | --- | --- | --- | --- | --- | --- | --- | --- | --- | --- | --- | --- | --- | --- | --- | --- | --- | --- | --- |
| **Effectiveness Outcome** | | | | | | | | | | | | | | | | | | | | | | | | |
| Andruska et al. 2020a | | 79 | | 73 | | 0.65 | |  | | 5 | 51 | | 41 | | 16 | | 12 | | 4.5 | 54 | | 25 | | 28.6 |
| Andruska et al. 2020b | | 72 | | 74 | | 0.8 | | 72 | | 7 | 0.54 | | 33 | | 24 | | 10 | |  | *50* | | *32* | | *20* |
| Andruska et al. 2021 | | 6156 | | 77 | | 0.594 | |  | | 191 |  | | 2732 | | 1904 | | 959 | | 4.5 |  | |  | | 35 |
| Bhatia et al. 2016 | | 6908 | | 76 | | 0.61 | |  | |  |  | | 3054 | |  | | 3125 | |  |  | | 2976 | |  |
| Björn et al. 2021 | | 113 | | 76 | | 0.43 | |  | |  |  | | 53 | | 24 | | 20 | |  | 66 | | 47 | | - |
| Blythe et al. 2014 | | 39 | | 78.7 | | 0.49 | |  | |  |  | |  | |  | |  | |  |  | |  | | 19.4 |
| Boyer et al. 2002 | | 45 | | 74 | |  | |  | |  |  | | 31 | |  | |  | | 12 | 25 | | 20 | | 24 |
| Cheraghlou et al. 2019 | | 736 | | 73 | | 0.674 | |  | |  |  | |  | |  | |  | |  | 211 | | 390 | |  |
| Dubois et al. 2021 | | 84 | | 79 | | 0.31 | |  | |  |  | | 50 | |  | |  | | 25 |  | | 31 | | 79.5 |
| Eich et al. 2002 | | 30 | | 73 | | 0.42 | | 13 | |  |  | | 13 | |  | |  | |  | 14 | | 16 | | 22 |
| Fang et al. 2010 | | 50 | | 68 | | 0.6 | | 50 | |  |  | | 3 | |  | |  | | 19 |  | | 22 | | 17 |
| Frohm et al. 2016 | | 104 | | - | | 0.53 | |  | | 6 | 24 | | 44 | | 22 | | 19 | |  |  | |  | | 34.3 |
| Gillenwater et al. 2001 | | 66 | | 68.4 | | 0.833 | | 7 | |  |  | | 66 | |  | |  | |  | 34 | | 26 | |  |
| Gorayski et al. 2017 | | 88 | | 68.1 | | 0.716 | |  | |  |  | | 33 | | 16 | | 16 | |  | - | | 13 | | 38.5 |
| Guenole et al. 2021 | | 77 | | 83 | | 0.44 | |  | | 28 | 10 | | 40 | | 9 | | 19 | |  |  | | 61 | | 25.7 |
| Han et al. 2018 | | 88 | | 71 | | 0.74 | |  | | 17 |  | | 38 | | 21 | | 13 | | 25 | 14 | | 42 | |  |
| Hoeller et al. 2014 | | 61 | | 68 | | 0.46 | | 25 | |  |  | | 9 | | 21 | | 12 | |  | 25 | | 26 | |  |
| Howle et al. 2011 | | 136 | | 75 | | 0.43 | |  | | 5 |  | | 64 | |  | | 40 | | 11 | 53 | | 69 | | 38 |
| Jaouen et al. 2021 | | 214 | | - | | 0.435 | | 48 | | 28 | 69 | | 77 | |  | | 109 | |  | 45 | | 169 | | 50.7 |
| Jouary et al. 2012 | | 83 | | 70.9 | | 0.36 | |  | |  |  | | 36 | | 14 | | 28 | |  |  | | 44 | | 58 |
| Kang et al. 2012 | | 62 | | 74 | | 0.68 | | 19 | | 14 |  | | 32 | | 8 | | 9 | | 15 | 19 | | 43 | |  |
| Kim et al. 2013 | | 747 | | 73.6 | | 0.57 | |  | |  |  | | 301 | | 203 | | 141 | |  | 343 | | 404 | |  |
| Kurian et al. 2021 | | 217 | | 79 | | 0.576 | | 26 | | 36 | 62 | | 99 | |  | |  | |  | 101 | | 116 | | 40 |
| Lamberti et al. 2021 | | 5644 | | 77 | | 0.623 | |  | |  |  | |  | |  | |  | | 17 | 2962 | | 2682 | |  |
| Lawenda et al. 2008 | | 36 | | 7.6 | | 0.61 | |  | |  |  | |  | |  | |  | | 17 | 14 | | 16 | | 30 |
| Levy et al. 2022 | | 182 | | 73.8 | | 0.56 | |  | |  | 30 | | 78 | |  | |  | |  | 88 | | 94 | | 34.5 |
| Meeuwissen et al. 1995 | | 80 | | 74 | | 0.65 | |  | |  |  | | 51 | | 7 | | 8 | |  |  | | 34 | | 20 |
| Mojica et al. 2007 | | 1665 | | 74 | | 0.59 | |  | |  |  | |  | |  | |  | | 20 | 689 | | 477 | | 40 |
| Morrison et al. 1990 | | 54 | | 70 | | 0.78 | |  | |  |  | | 38 | |  | |  | |  | 37 | | 12 | |  |
| Mortier et al. 2003 | | 26 | | 81 | | 0.31 | |  | |  |  | | 14 | |  | |  | | 29.5 |  | | 16 | | 48 |
| Pape et al. 2011 | | 50 | | 75.14 | | 0.24 | |  | |  |  | | 31 | |  | |  | | 17.8 | 25 | | 25 | | 76.8 |
| Poulsen et al. 2010 | | 60 | | 76 | | 0.43 | |  | |  |  | |  | |  | |  | |  |  | | 62 | |  |
| Poulsen et al. 2006 | | 102 | | 69.75 | | 0.7 | |  | |  |  | | 22 | | 8 | | 8 | |  | 17 | |  | |  |
| Servy et al. 2016 | | 87 | | 70.7 | | 0.437 | |  | | 8 |  | | 27 | |  | |  | |  | 12 | | 75 | | 39.3 |
| Sexton et al. 2014 | | 42 | | 70.5 | | 0.74 | | 16 | | 5 | 16 | | 17 | |  | |  | |  | 24 | | 8 | | 42 |
| Singh et al. 2018 | | 1795 | | - | | 0.582 | |  | |  |  | | 761 | |  | |  | |  | - | | 747 | |  |
| Strom et al. 2016a | | 171 | | 74 | | 0.725 | |  | | 17 | 52 | | 71 | | 51 | | 31 | | 15 | - | |  | | 33 |
| Strom et al. 2016b | | 113 | | 77 | | 0.752 | | 25 | | 18 |  | | 39 | |  | |  | | 10 |  | |  | | 27 |
| Takagishi et al. 2016 | | 46 | | 66.5 | | 0.5 | |  | |  | 54 | |  | |  | |  | | 8 | 23 | | 23 | | 44.4 |
| Tarabadkar et al. 2021 | | 188 | | 65 | | 0.617 | | 45 | | 18 | 164 | | 66 | |  | |  | |  | 48 | | 140 | | 48 |
| Tarantola et al. 2013 | | 240 | | 70.1 | | 0.7 | | 32 | |  | 74 | | 111 | | 45 | | 46 | | 22 |  | |  | |  |
| Vargo et al. 2017 | | 14414 | |  | |  | |  | |  |  | |  | |  | |  | |  | 3150 | | 3033 | | 35 |
| Veness et al. 2004 | | 37 | | 75 | | 0.65 | | 8 | |  |  | | 37 | |  | |  | | 12 | 16 | | 20 | | 26 |
| Veness et al. 2005 | | 86 | | 75 | | 0.57 | | 35 | | 1 |  | | 47 | | 17 | | 12 | | 12 | 36 | | 38 | | 31 |
| Wong et al. 2021 | | 2330 | |  | | 0.624 | |  | |  |  | |  | | 307 | | 2023 | |  | 1065 | | 1265 | |  |
| Wright et al. 2018 | | 2454 | | 70 | | 0.565 | |  | |  |  | | 222 | | 302 | | 978 | | 85 | 1227 | | 1227 | |  |
| **Safety outcome** | | | | | | | | | | | | | | | | | | | | | | | | |
| **Authors** | **No of patients who experienced toxicity** | | **Event measured** | | **Grade** | | **Freq (Control group)** | | **Freq (Intervention group)** | | | **Neutrophils** | | **Freq (Control group)** | | **Freq (Intervention group)** | | **White Cell count** | | | **Freq (Control group)** | | **Freq (Intervention group)** | |
| Dubois et al.2021 | 7 | |  | |  | |  | |  | | |  | |  | |  | |  | | |  | |  | |
| Gorayski et al. 2015 | 41 | | Skin toxicity and white cell toxicity | | ≥ 3 | | No CT Group: 4/13 | | CT Group: 37/75 | | | ≥3 | | 8/13 | | 36/75 | | ≥3 | | | 4/13 | | 37/75 | |
| Hoeller et al. 2014 | 2 | |  | |  | |  | |  | | |  | |  | |  | |  | | |  | |  | |
| Jouary et al. 2011 | 22 | | Skin toxicity | | Grade 1: 32; Grd 2: 12; Grade 3: 1 | | Group A (N=39); Grade 1:15.4%; Grade 2: 7.7% | | Group B (N=44); Grade 1:22.7%; Grade 2: 6.8% | | |  | |  | |  | |  | | |  | |  | |
| Takagishi et al. 2016 | 0 | | Skin toxicity | |  | |  | |  | | |  | |  | |  | |  | | |  | |  | |
| Poulsen et al. 2001 | 40 | | Toxicity | | Grade 3/4: N:63%; | |  | |  | | | Grade 3/4: 60% | |  | |  | |  | | |  | |  | |
| Poulsen et al. 2008 | 11 | | Toxicity | | Grade 3/4: N = 4 | |  | |  | | | Grade 3/4: N=7 | |  | |  | | Grade 2: N=6; Grade 3/4: N=4 | | |  | |  | |
