# Supplementary material for: Effectiveness and Safety of Treatments for Early‐Stage Merkel Cell Carcinoma: A Systematic Review and Meta‐Analysis of Randomized and Non‐Randomized Studies
Source: Cancer Med. 2025 Jan 3;14(1):e70553. doi: 10.1002/cam4.70553 (PMC11696246; doi:10.1002/cam4.70553)
Supplement: Supplementary file 4 — Appendix S4. [file CAM4-14-e70553-s001.docx]

| **Certainty assessment** | | | | | | | **Number of patients** | | **Effect** | | **Certainty** | **Importance** |
| --- | --- | --- | --- | --- | --- | --- | --- | --- | --- | --- | --- | --- |
| **№ of studies** | **Study design** | **Risk of bias** | **Inconsistency** | **Indirectness** | **Imprecision** | **Other considerations** | **Surgery and Adjuvant Radiotherapy** | **Surgery** | **Relative (95% CI)** | **Absolute (95% CI)** |  |  |
| **OS (follow-up: median 35 months; assessed with: time to event)** | | | | | | | | | | | | |
| 31 | non-randomized studies and one open label randomized trial | serious^a^ | serious^b^ | not serious | not serious | publication bias strongly suspected strong association dose response gradient^c^ | 13252 participants | 12969 participants | **HR 0.78**  (0.62 to 0.99)  [OS] | **89 more per 1,000**  (from 3 more to 173 more) | ⨁⨁⨁◯ Moderate | CRITICAL |
| **LR (follow-up: median 17 months; assessed with: time to event)** | | | | | | | | | | | | |
| 6 | non-randomized studies | serious | serious^d^ | not serious | not serious | publication bias strongly suspected dose response gradient^e^ | 367 participants | 181 participants | **HR 1.52**  (0.37 to 6.19)  [LR] | **119 more per 1,000**  (from 176 fewer to 590 more) | ⨁⨁◯◯ Low | CRITICAL |
| **RR (follow-up: median 34.5 months; assessed with: time to event)** | | | | | | | | | | | | |
| 9 | non-randomized studies | serious^a^ | serious^f^ | not serious | not serious | dose response gradient | 423 participants | 231 participants | **HR 0.41**  (0.09 to 1.78)  [RR] | **75 fewer per 1,000**  (from 118 fewer to 90 more) | ⨁⨁⨁◯ Moderate | CRITICAL |
| **DSS (follow-up: median 34.5 months; assessed with: time to event)** | | | | | | | | | | | | |
| 16 | non-randomized studies | serious^a^ | serious^g^ | not serious | not serious | publication bias strongly suspected dose response gradient^g^ | 1139 participants | 830 participants | **HR 0.58**  (0.24 to 1.40)  [DSS] | **136 more per 1,000**  (from 41 fewer to 449 more) | ⨁⨁◯◯ Low | CRITICAL |
| **DFS (follow-up: median 31 months; assessed with: time to event)** | | | | | | | | | | | | |
| 12 | non-randomized studies | serious^a^ | serious^h^ | not serious | not serious | dose response gradient | 512 participants | 403 participants | **HR 0.35**  (0.13 to 0.93)  [DFS] | **289 more per 1,000**  (from 25 more to 421 more) | ⨁⨁⨁◯ Moderate | CRITICAL |

**CI:** confidence interval; **HR:** hazard Ratio

#### Explanations

a. The MASTER instrument revealed serious flaws with respect to the safeguards of equal retention, equal ascertainment, equal prognosis, and temporal precedence. These flaws pertained mostly to selection bias, analytic bias, information bias, confounding, and design-related bias.

b. Per the quantitative analysis, we uncovered inconsistency values higher than 99%

c. The Luis Furuya Kanamori (LFK) was -1.64 indicative of minor asymmetry, which suggests small study effects.

d. Inconsistency was 85.4% for Local Recurrence.

e. The LFK was 1.19 indicative of minor asymmetry, which suggests small study effects.

f. Inconsistency was 90.1% for Reginal Recurrence.

g. The LFK was 1.42 indicative of minor asymmetry, which suggests small study effects.

h. Inconsistency was 83.5% for Disease Free Survival.
