# Supplementary material for: Effectiveness and Safety of Treatments for Early‐Stage Merkel Cell Carcinoma: A Systematic Review and Meta‐Analysis of Randomized and Non‐Randomized Studies
Source: Cancer Med. 2025 Jan 3;14(1):e70553. doi: 10.1002/cam4.70553 (PMC11696246; doi:10.1002/cam4.70553)
Supplement: Supplementary file 5 — Appendix S5. [file CAM4-14-e70553-s003.docx]

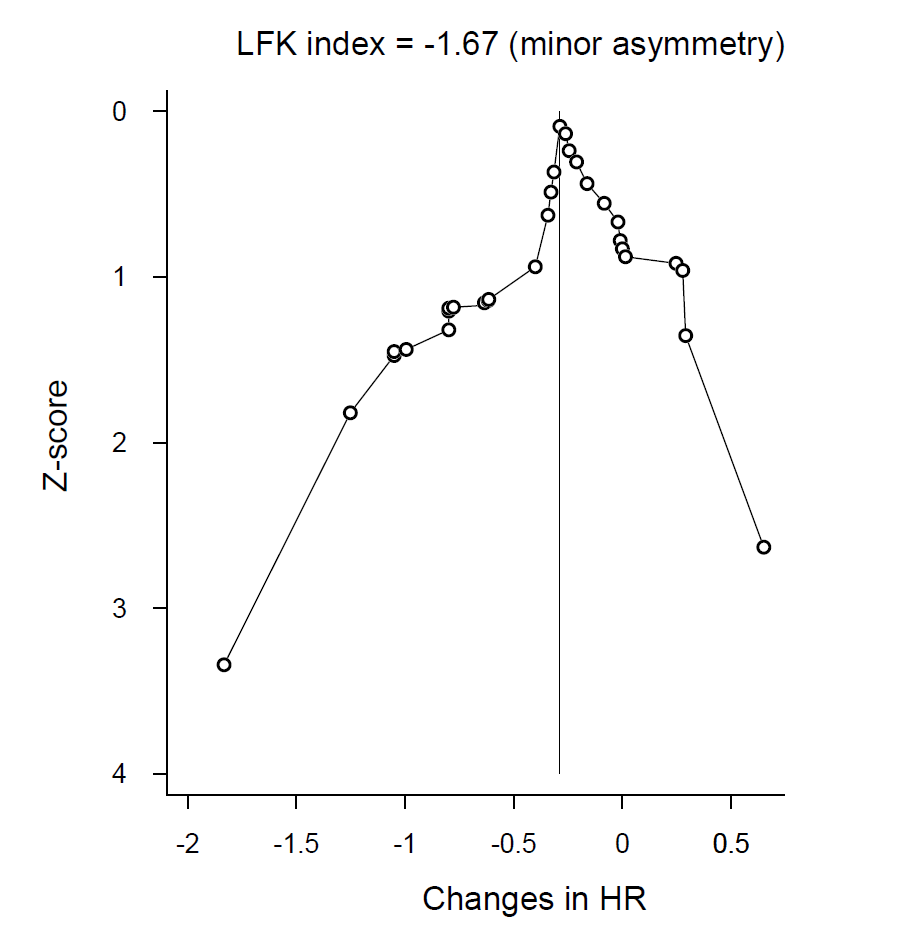


**Figure 1A:** Doi plot for changes in OS for adjuvant RTx.


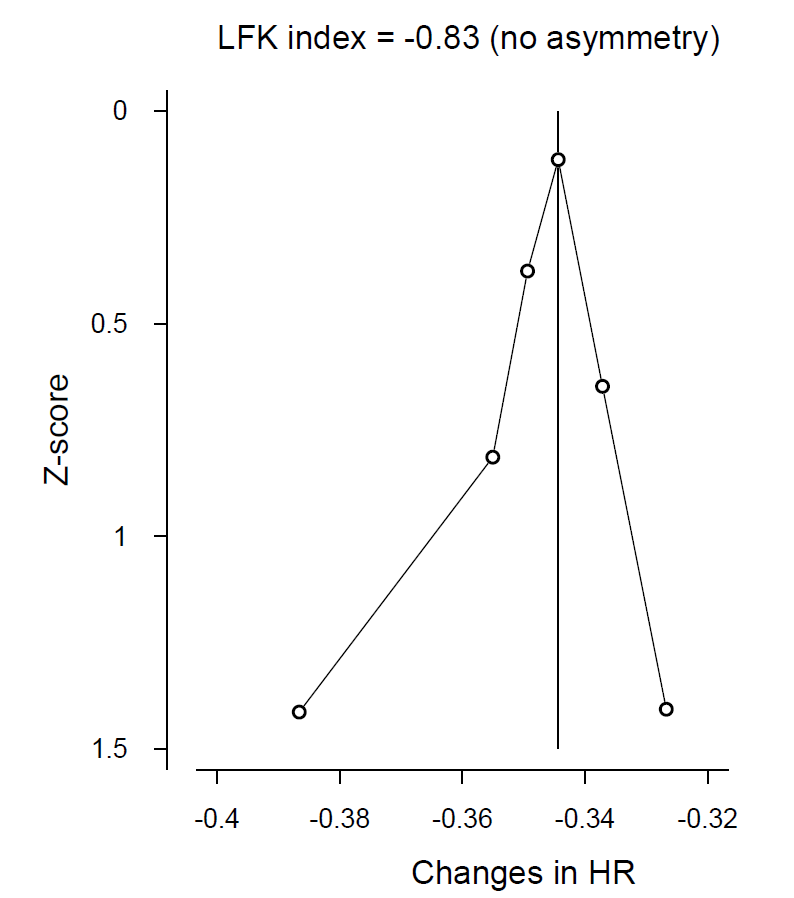


**Figure 1B:** Doi plot for changes in OS for the addition of CTx.


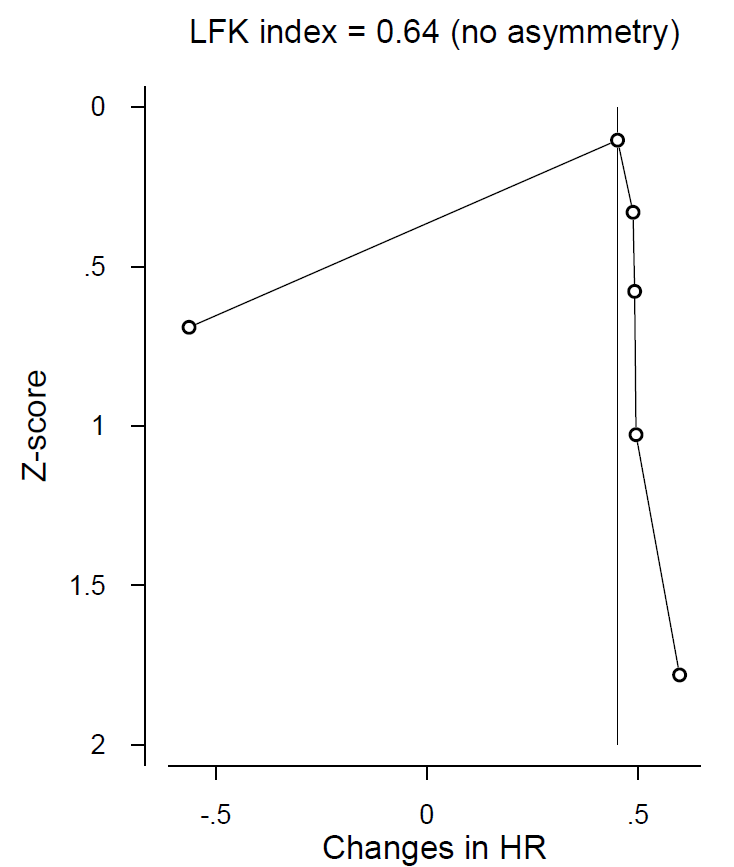


**Figure 1C:** Doi plot for changes in LR.


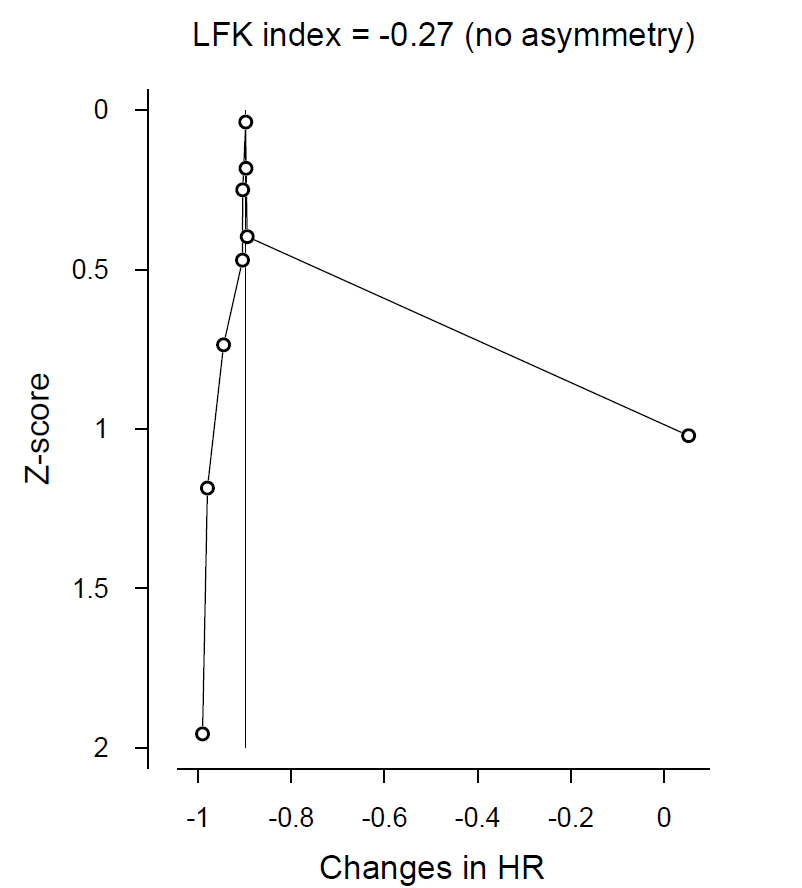


**Figure 1D:** Doi plot for changes in RR.


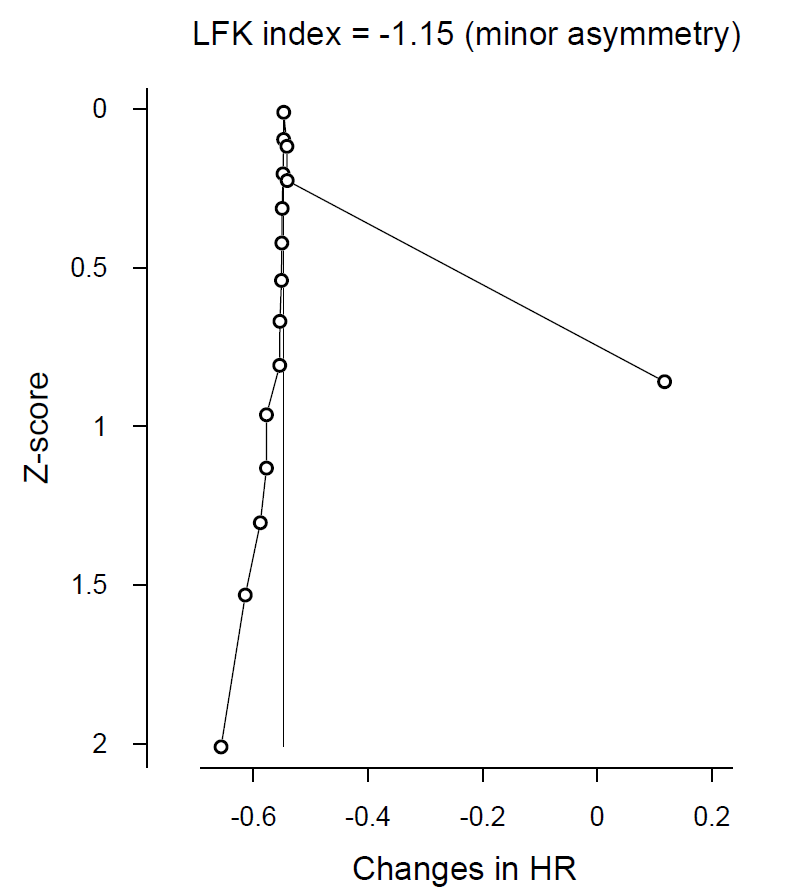


**Figure 1E:** Doi plot for changes in DSS.


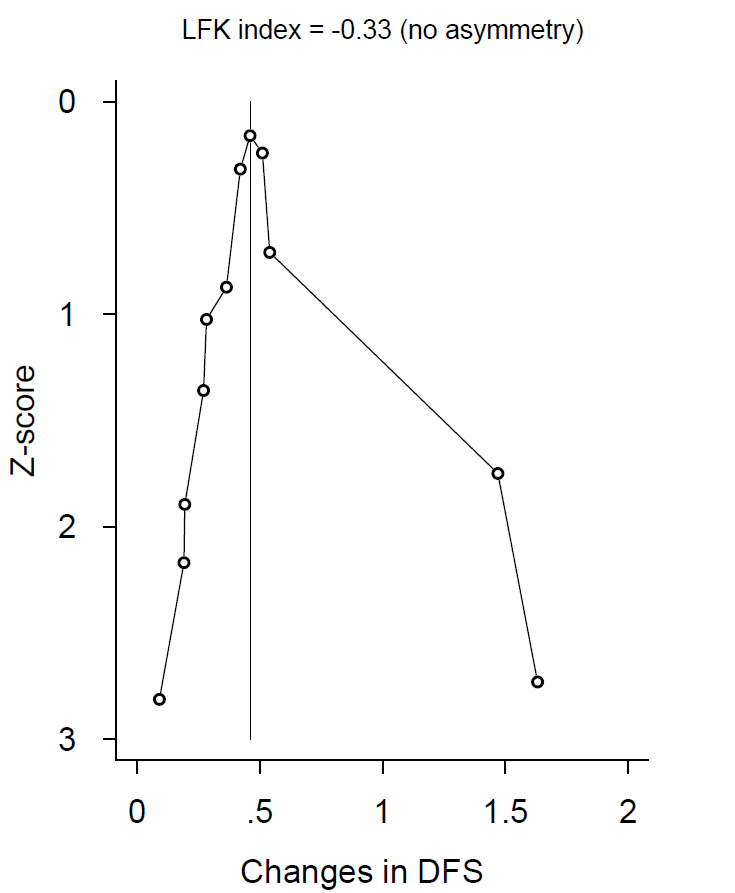


**Figure 1F:** Doi plot for changes in DFS.


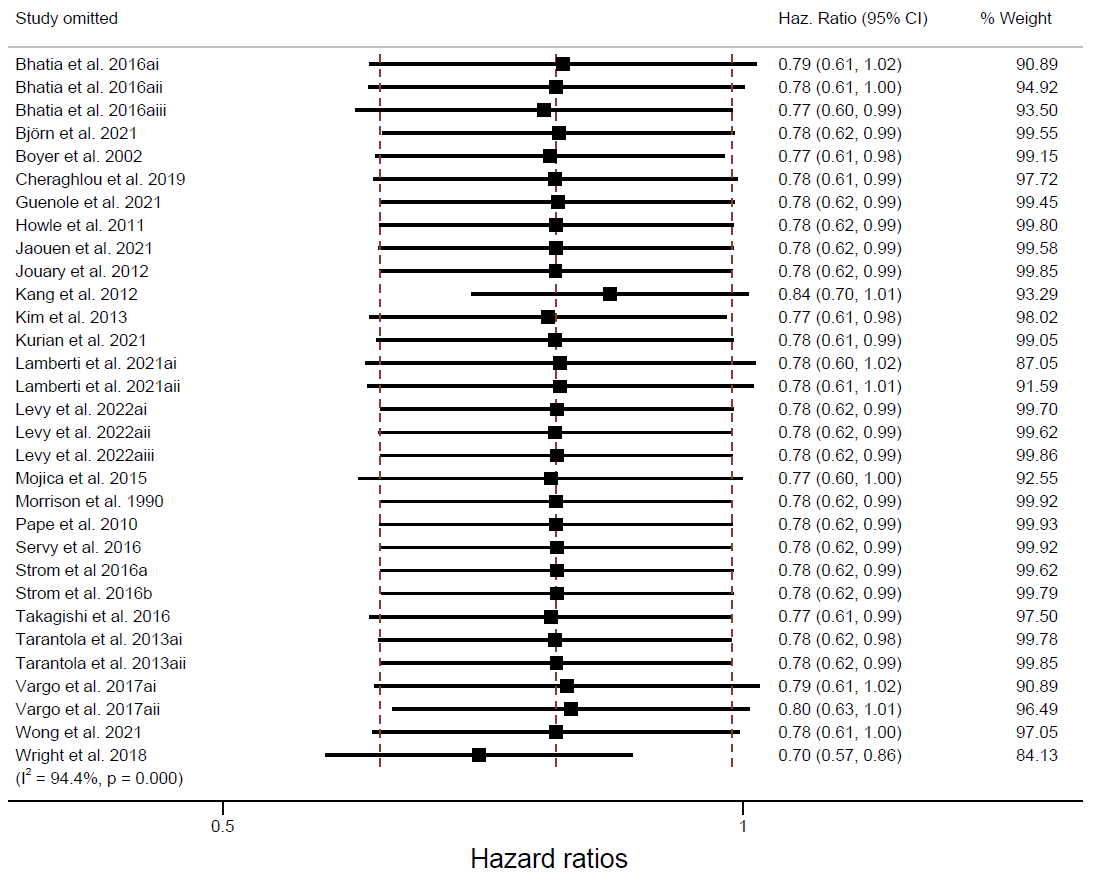


**Figure 2A:** Influence meta-analysis for changes in OS for adjuvant RTx.


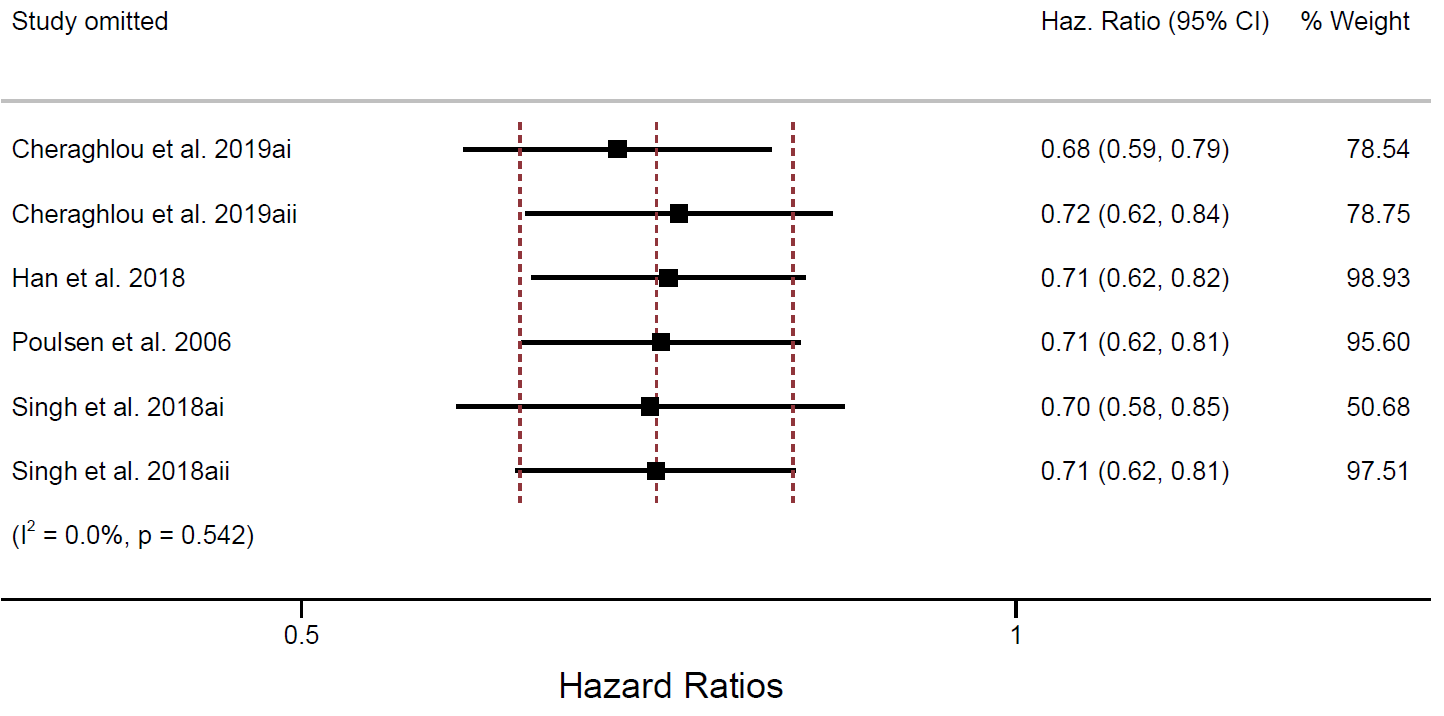


**Figure 2B:** Influence meta-analysis for changes in OS for adjuvant CTx.


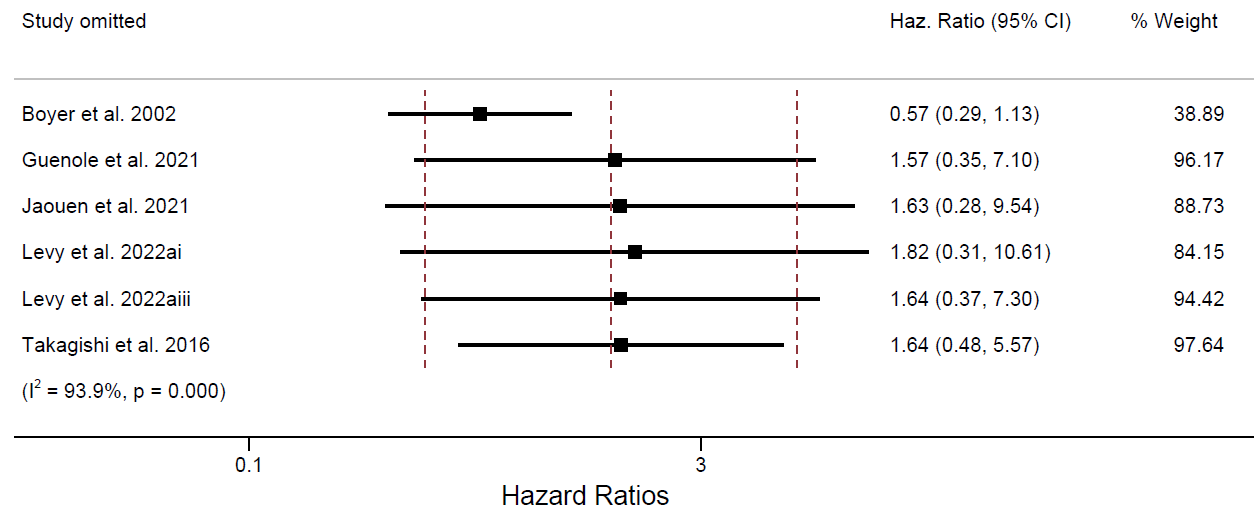


**Figure 2C:** Influence meta-analysis for changes in LR.


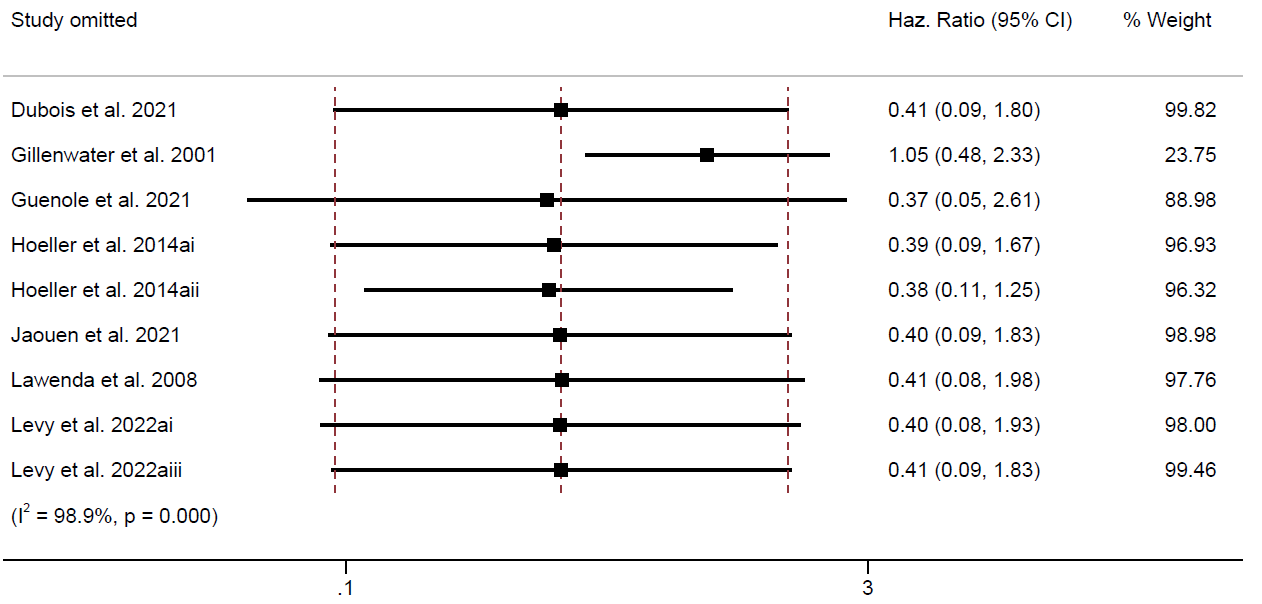


**Figure 2D:** Influence meta-analysis for changes in RR .


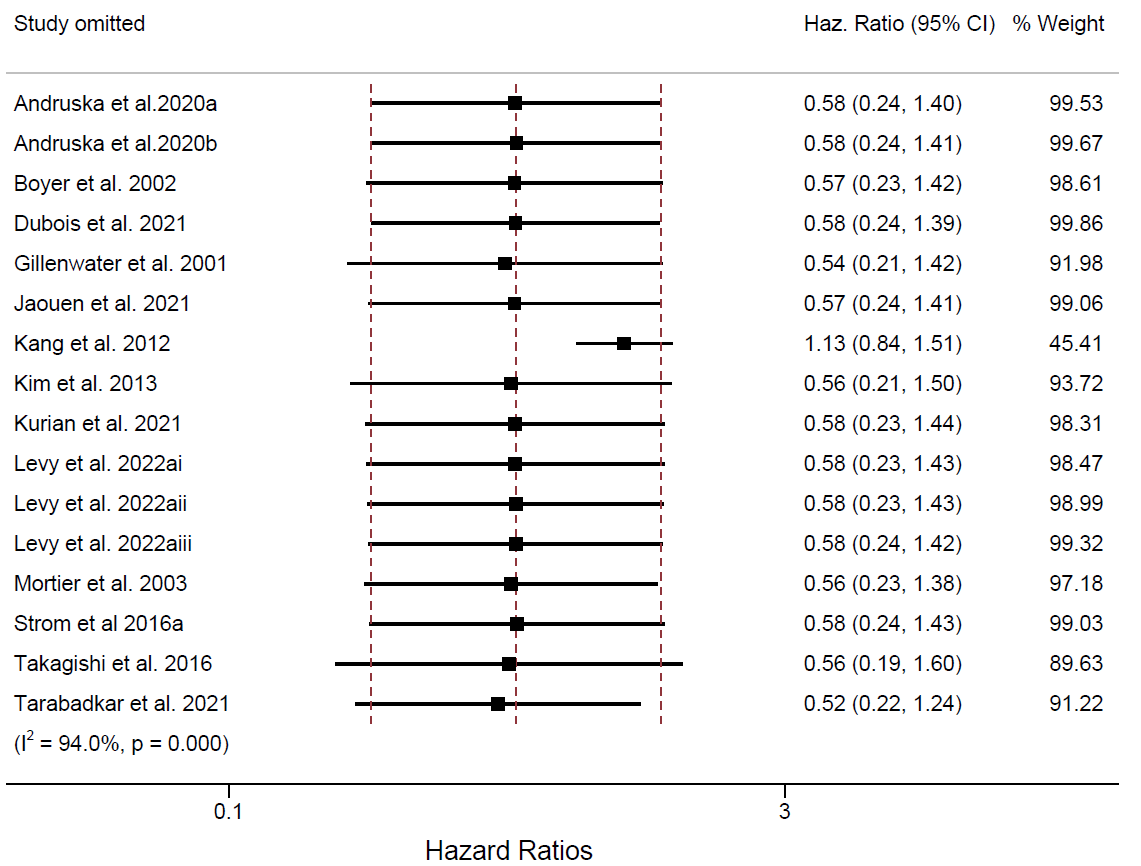


**Figure 2E:** Influence meta-analysis for changes in DSS.


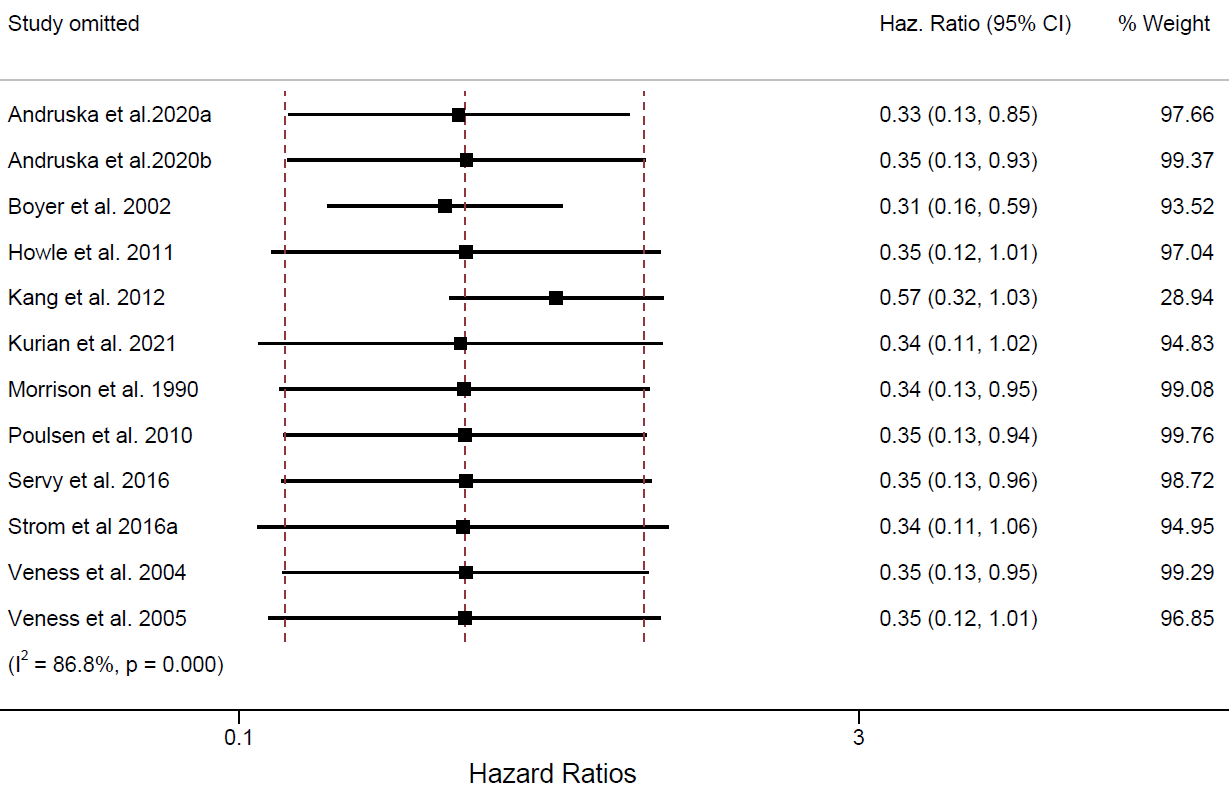


**Figure 2F:** Influence meta-analysis for changes in DFS.


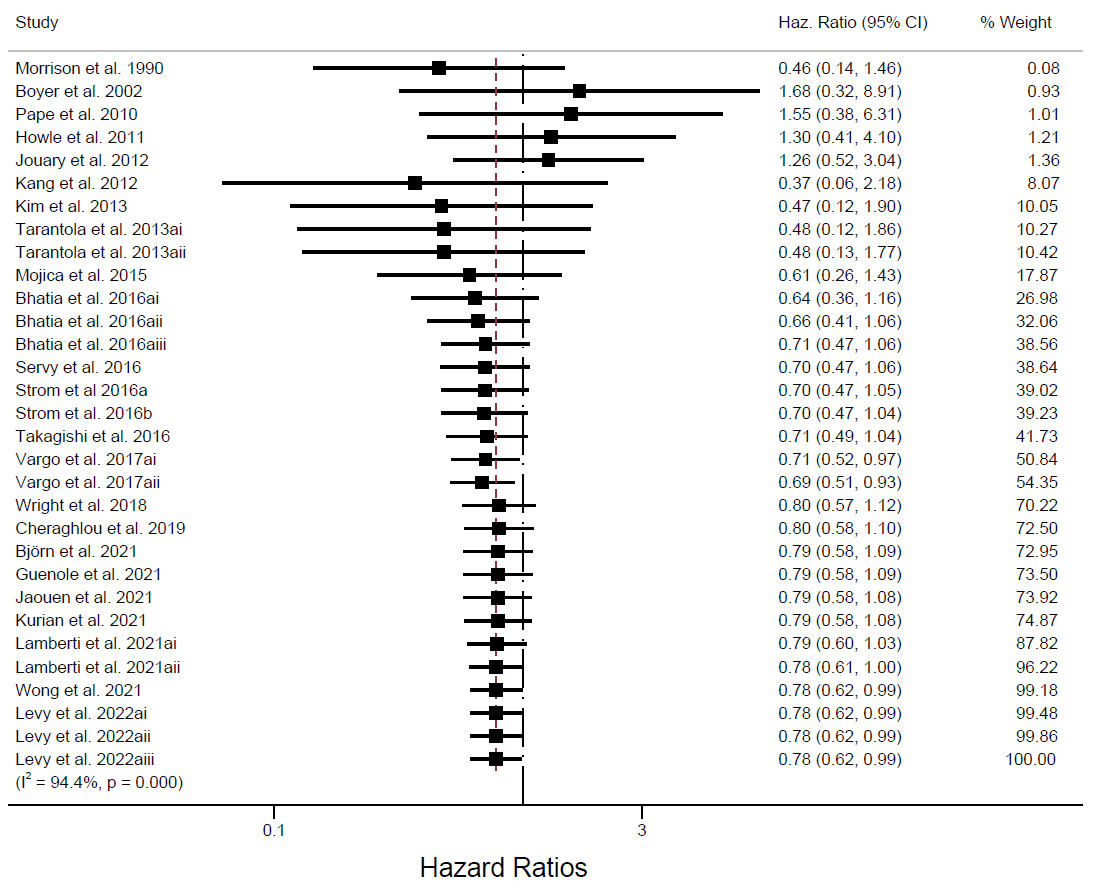


**Figure 3A:** Cumulative meta-analysis for changes in OS for adjuvant RTx.


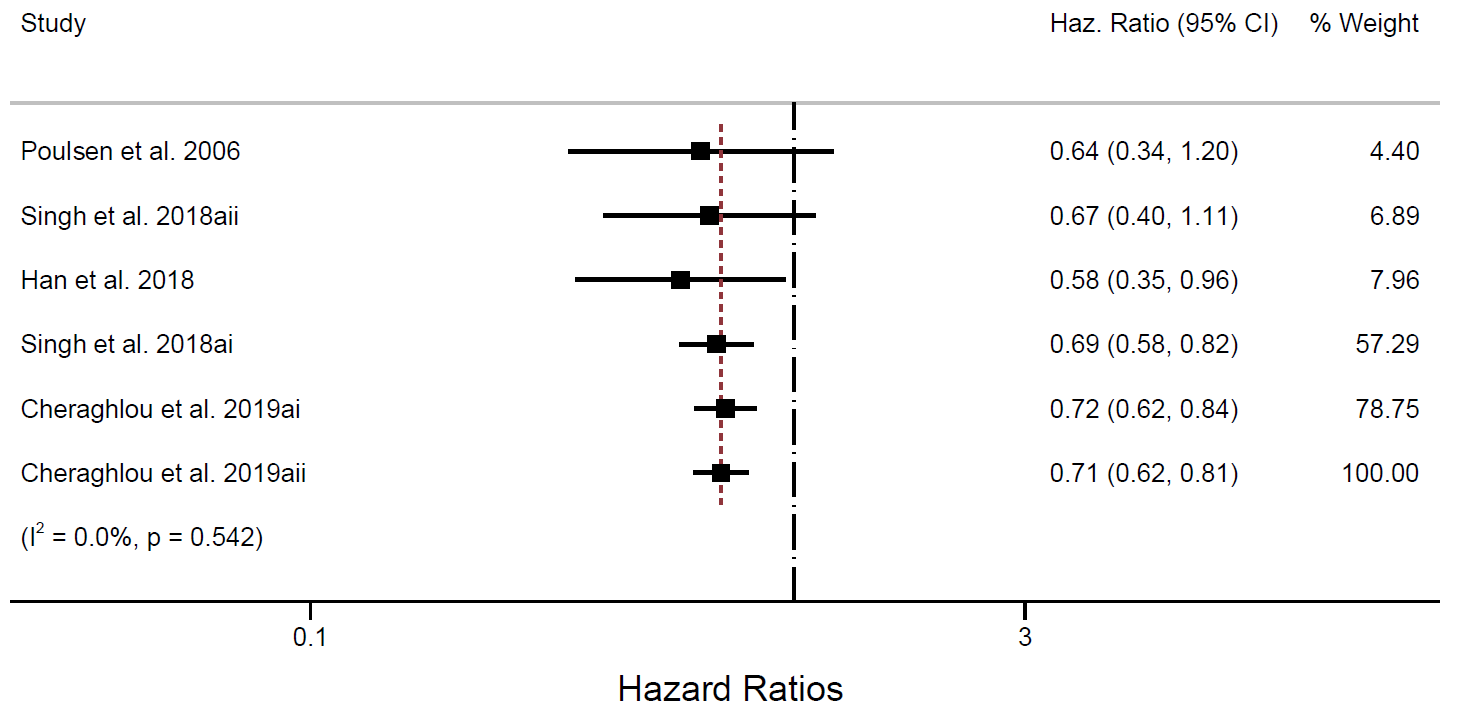


**Figure 3B:** Cumulative meta-analysis for changes in OS for adjuvant CTx.


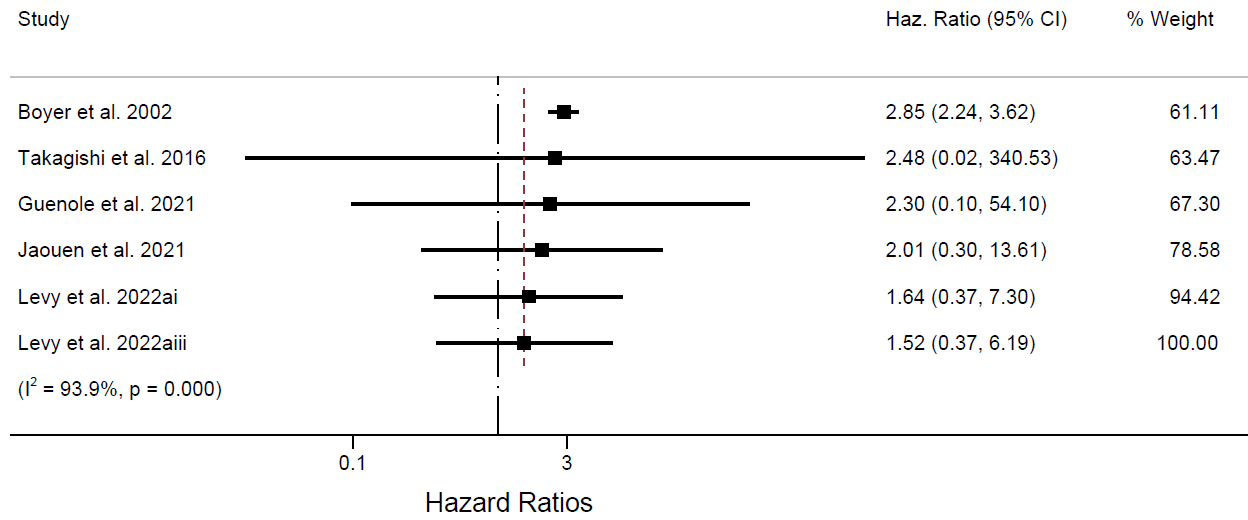


**Figure 3C:** Cumulative meta-analysis for changes in LR.


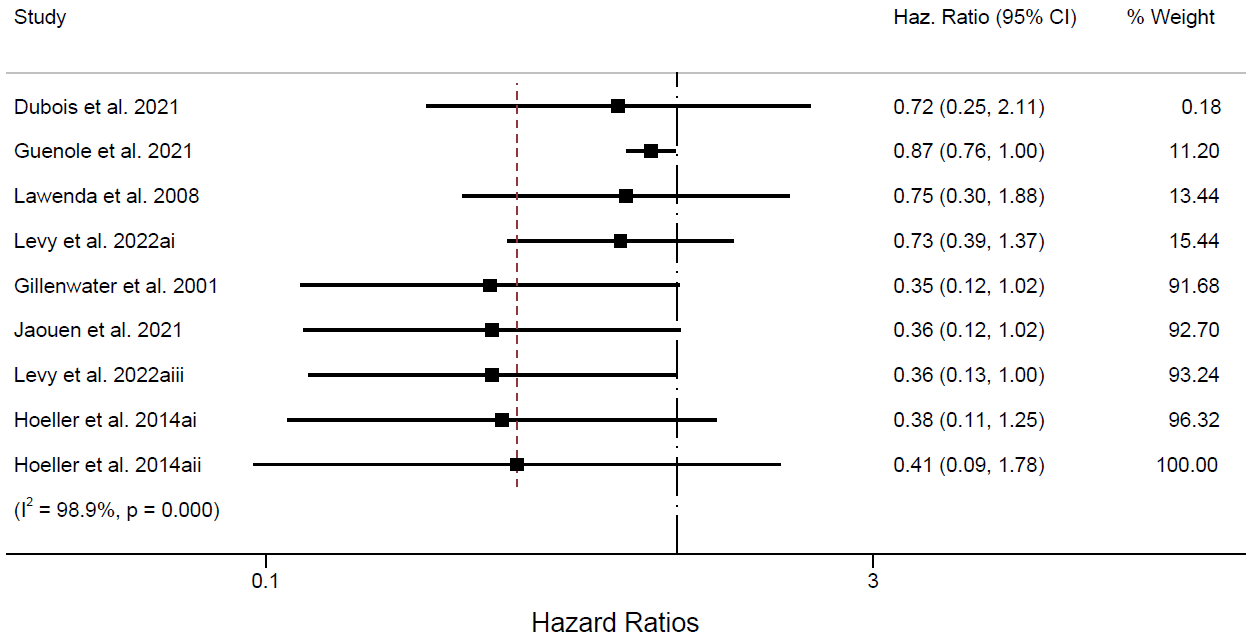


**Figure 3D:** Cumulative meta-analysis for changes in RR.


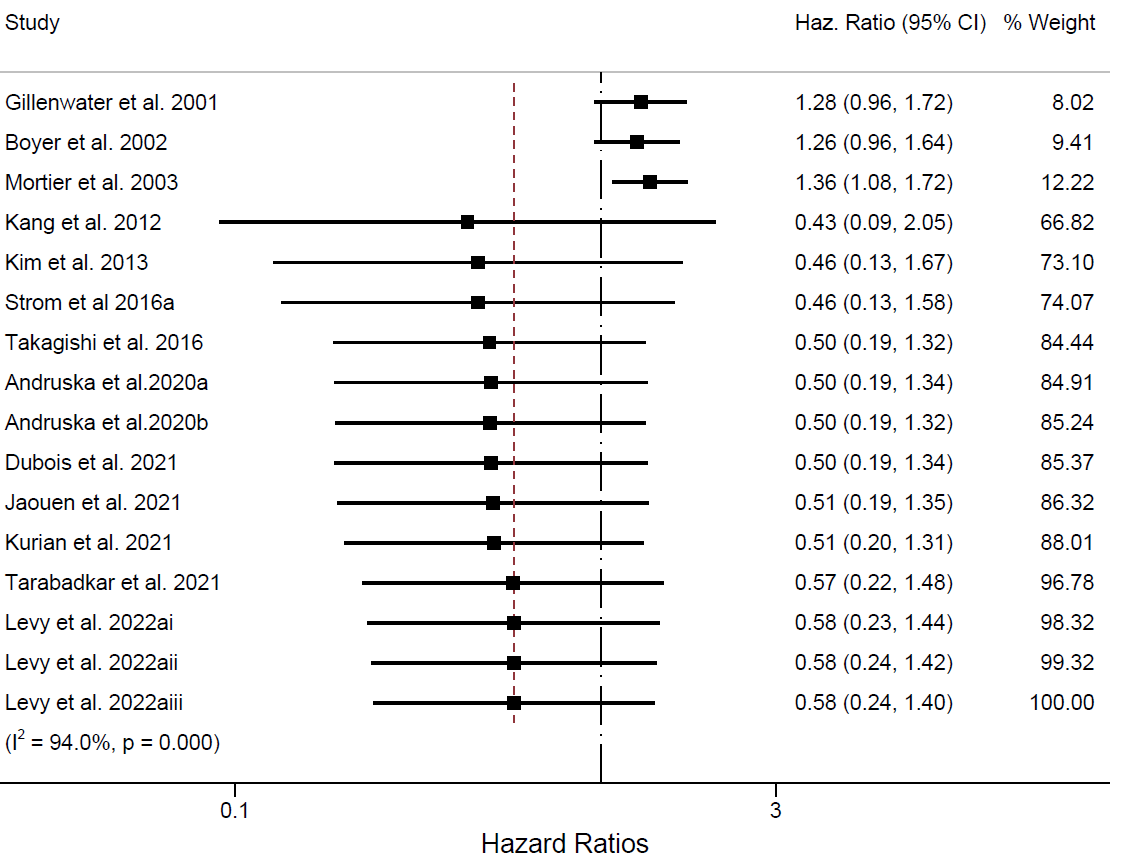


**Figure 3E:** Cumulative meta-analysis for changes in DSS.


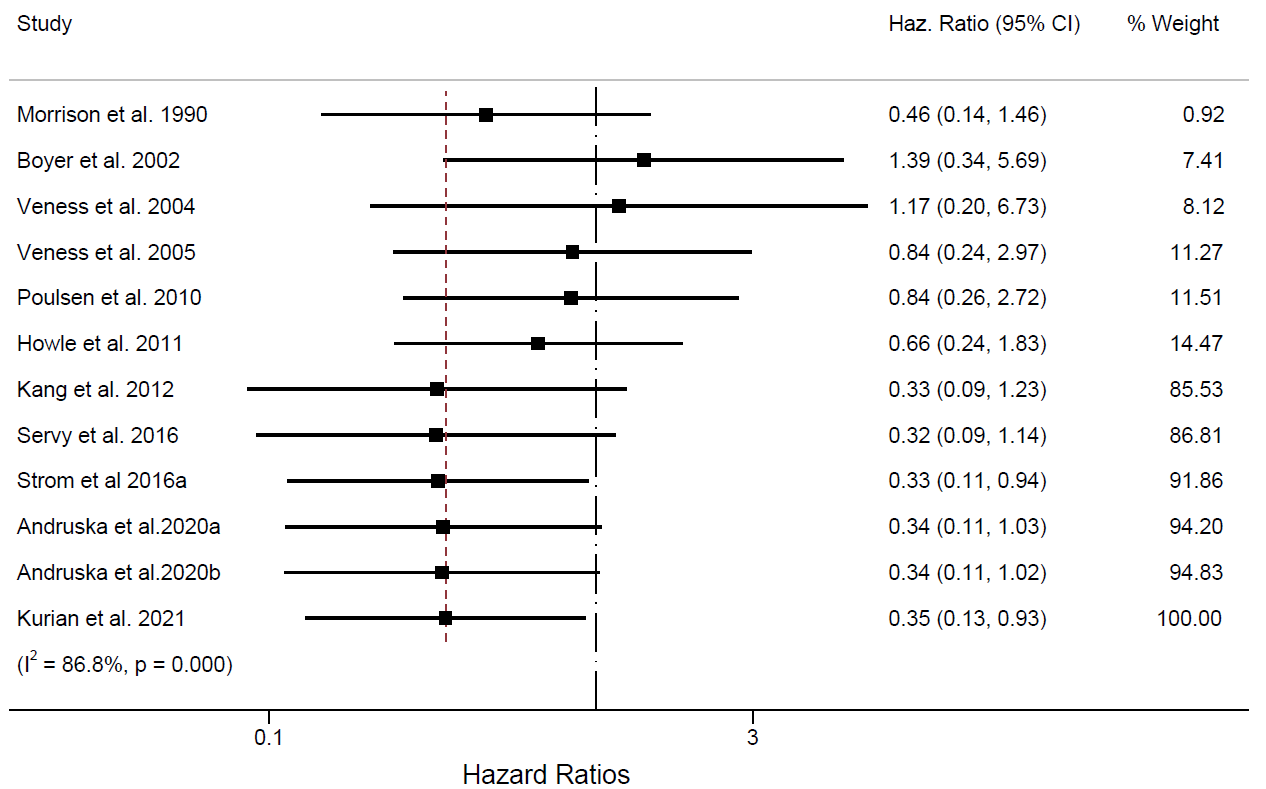


**Figure 3F:** Cumulative meta-analysis for changes in DFS.


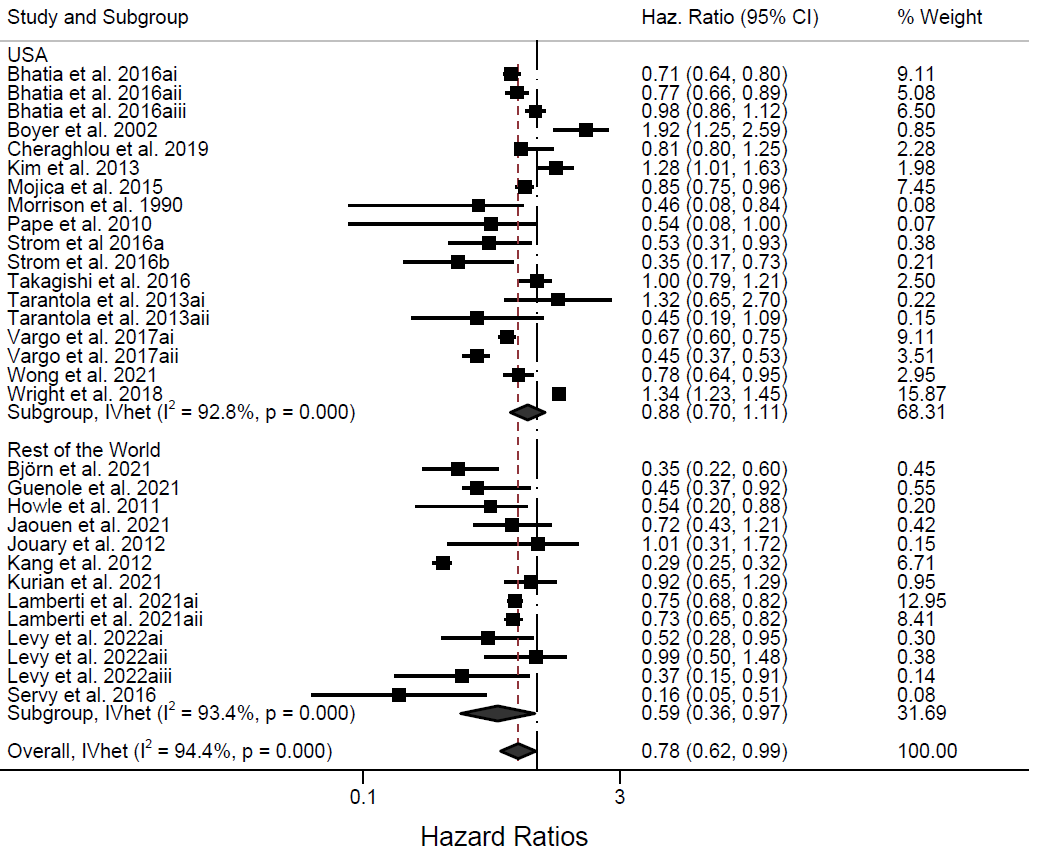


**Figure 4A:** Subgroup analysis of OS (adjuvant RTx) per geographical location


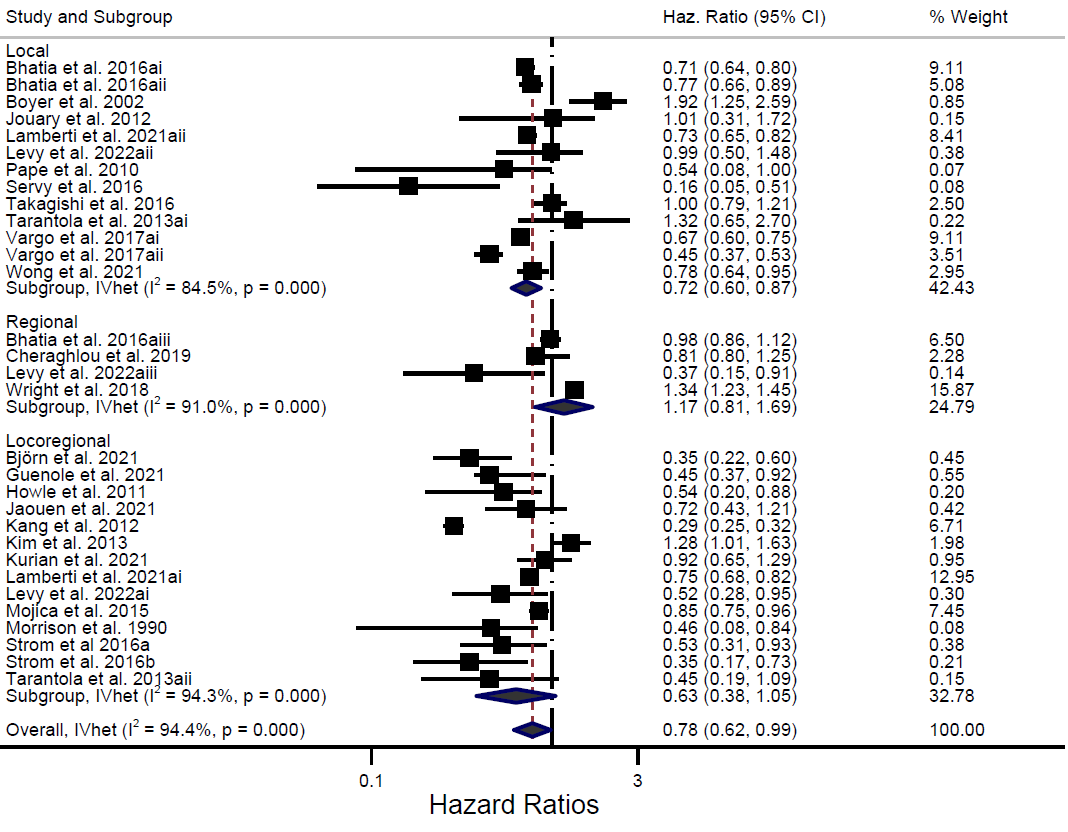


**Figure 4B:** Subgroup analysis of OS (adjuvant RTx) per MCC stage


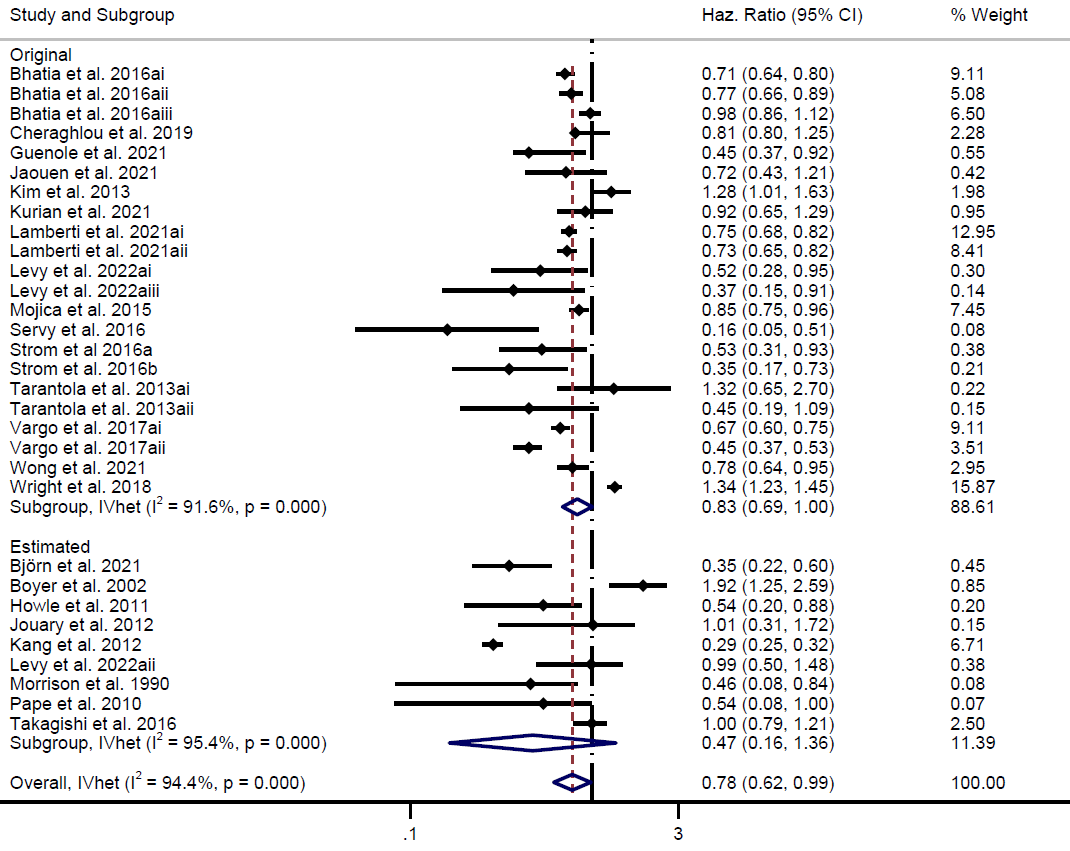


**Figure 4C:** Subgroup analysis of OS (adjuvant RTx) per HR calculation method


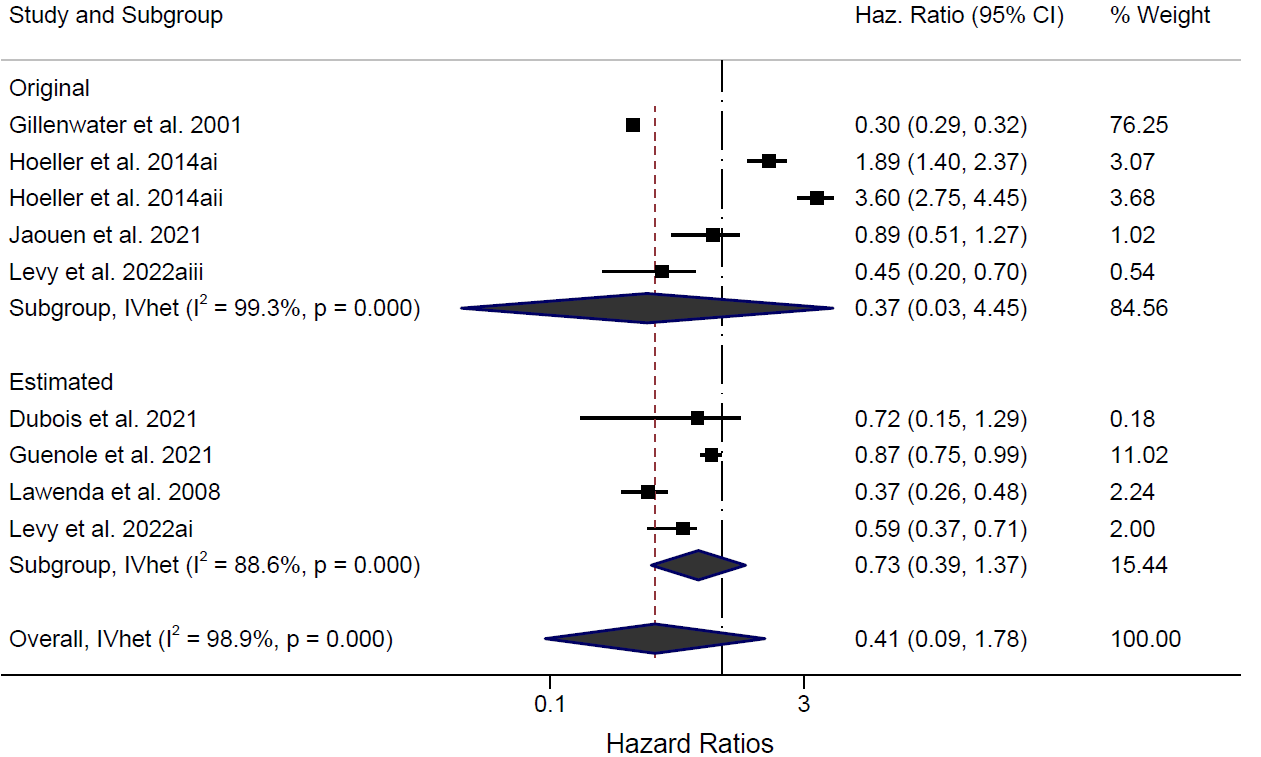


**Figure 5:** Subgroup analysis of RR per HR calculation method


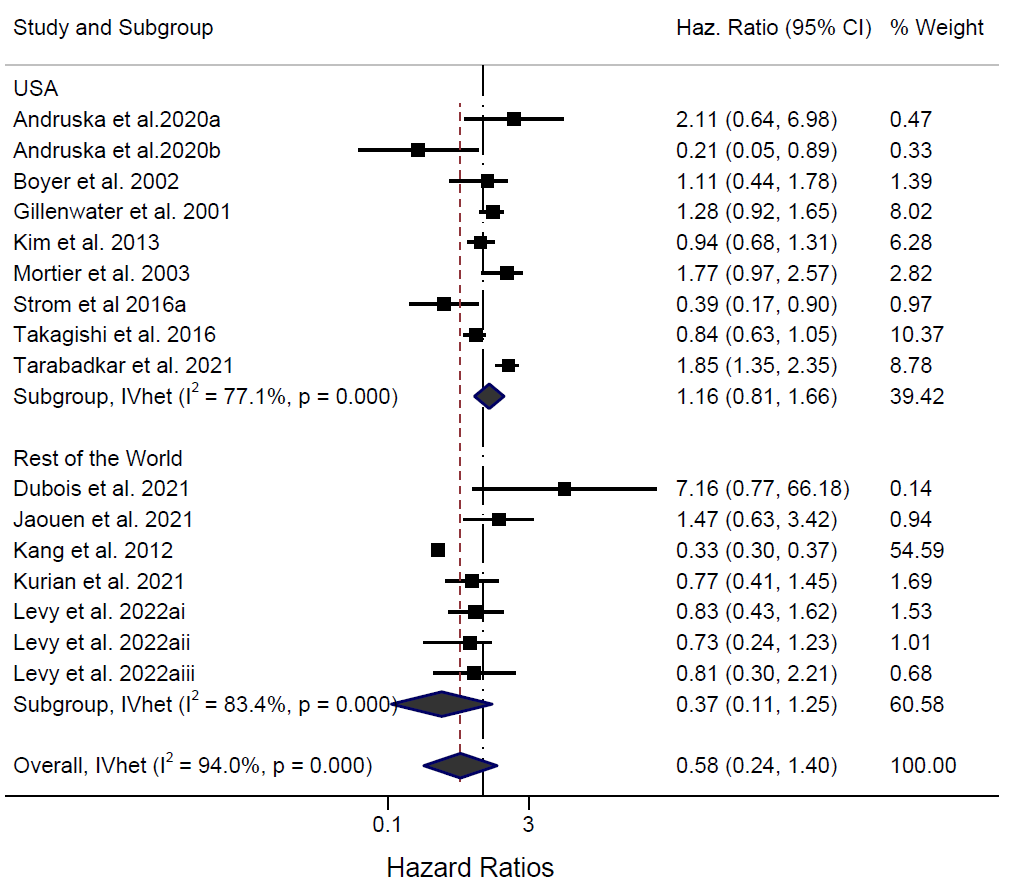


**Figure 6A:** Subgroup analysis of DSS per geographical location


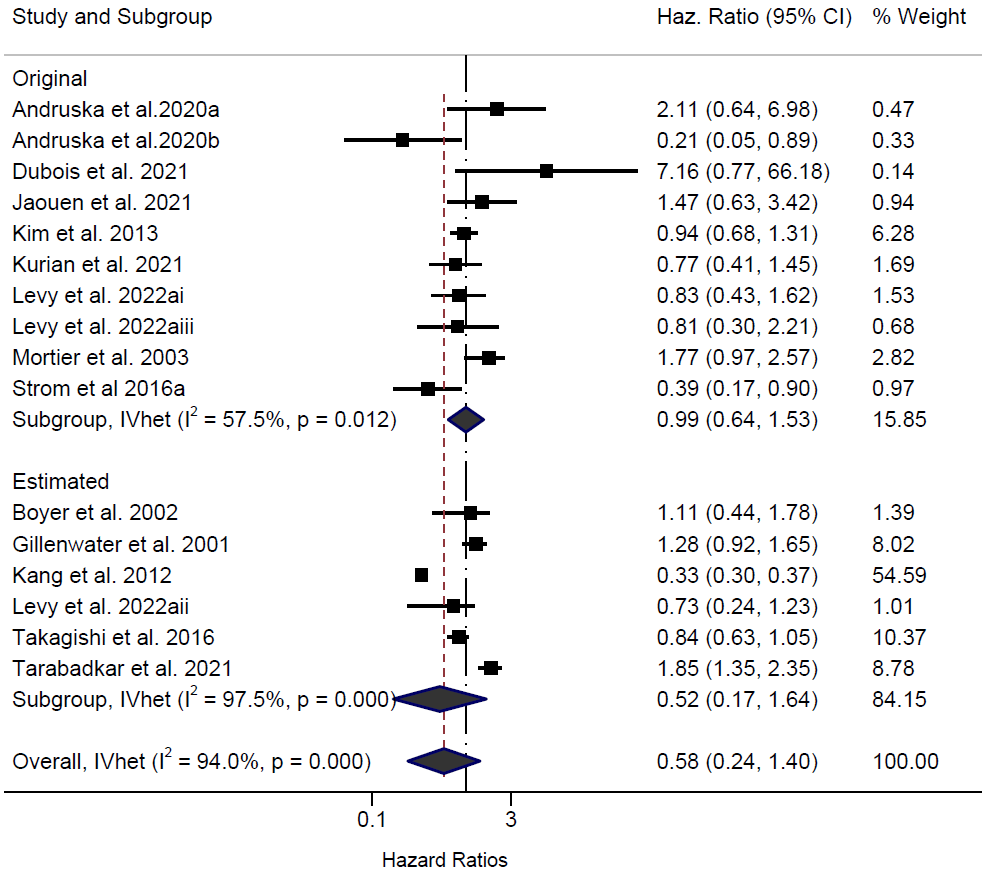


**Figure 6B:** Subgroup analysis of DSS per HR calculation method


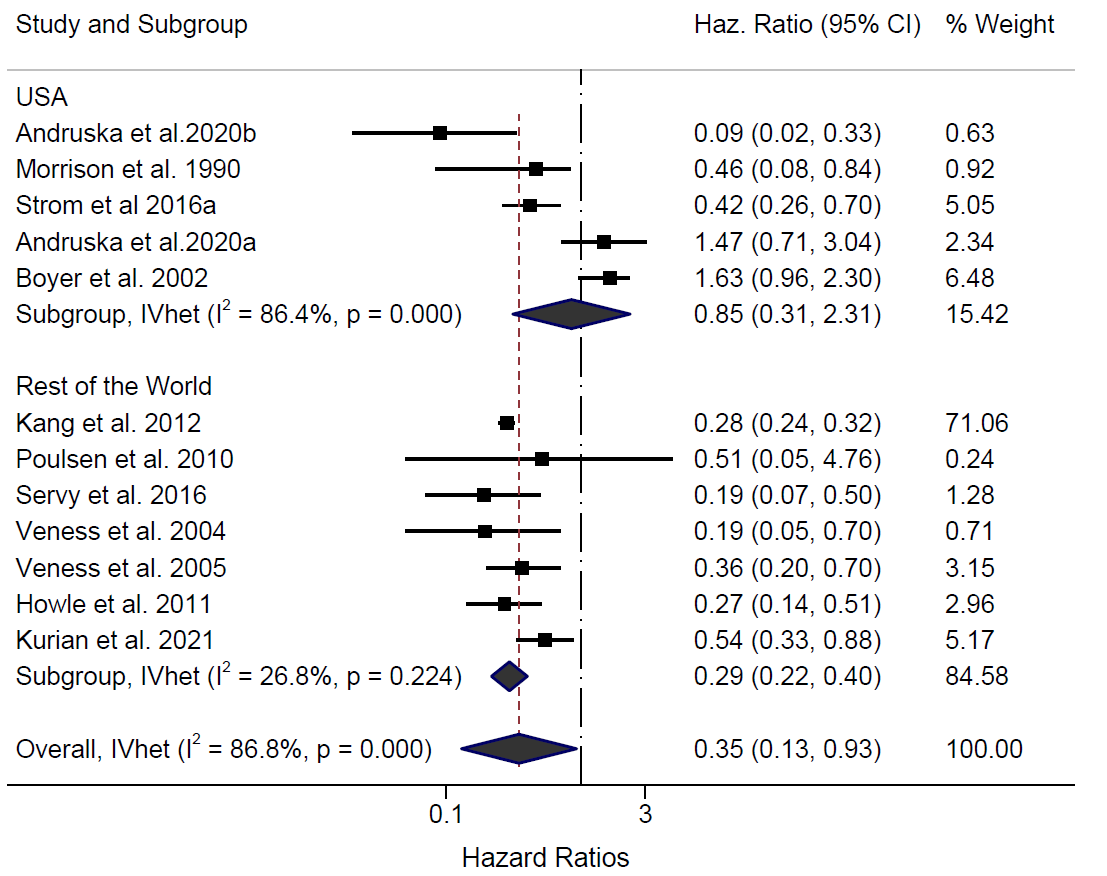


**Figure 7A:** Subgroup analysis of DFS per geographical location


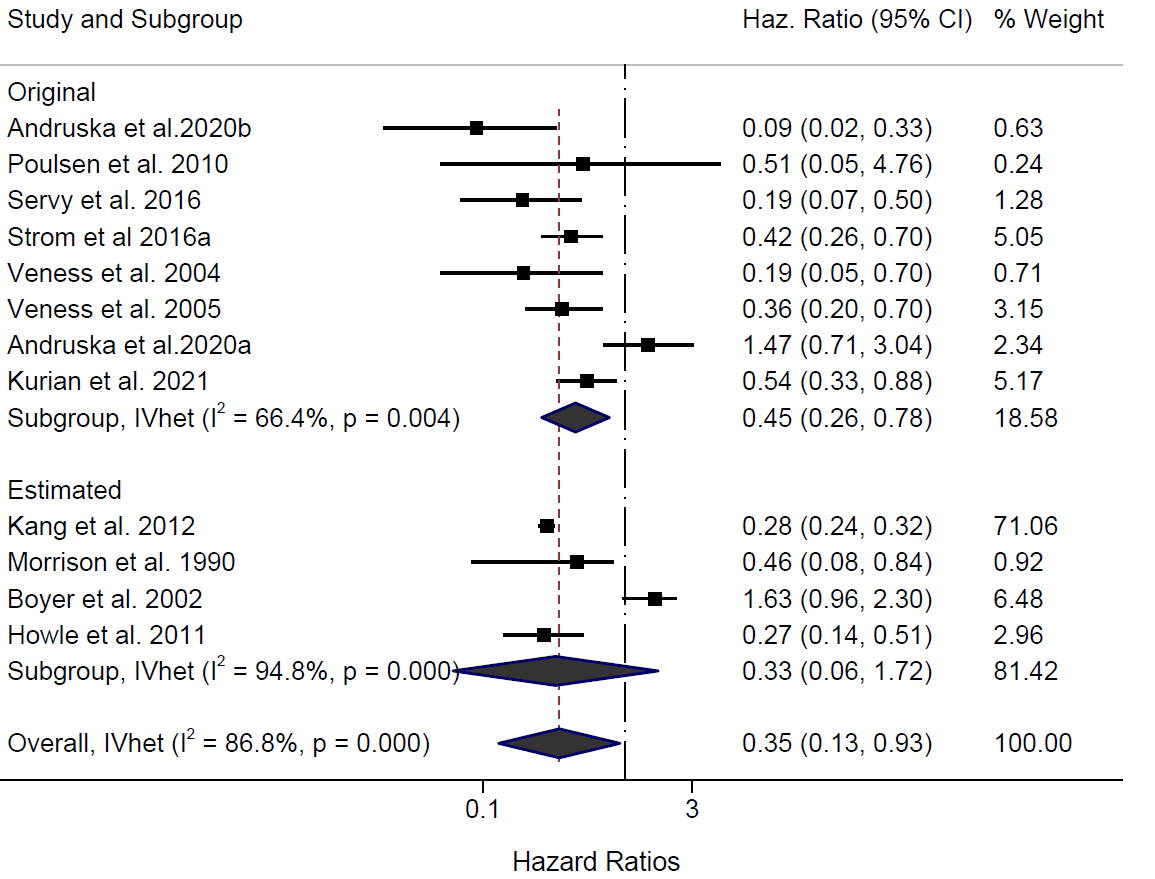


**Figure 7B:** Subgroup analysis of DFS per HR calculation method
