# Supplementary material for: Effectiveness and Safety of Treatments for Early‐Stage Merkel Cell Carcinoma: A Systematic Review and Meta‐Analysis of Randomized and Non‐Randomized Studies
Source: Cancer Med. 2025 Jan 3;14(1):e70553. doi: 10.1002/cam4.70553 (PMC11696246; doi:10.1002/cam4.70553)
Supplement: Supplementary file 6 — Appendix S6. [file CAM4-14-e70553-s005.docx]

| **Table 1:** Magnitude of difference between subgroups for changes in various outcomes | | | | | | | | | | |
| --- | --- | --- | --- | --- | --- | --- | --- | --- | --- | --- |
| **OS (SRx vs. SRx+RTx)** | | | | | | | | | | |
| **Geographical location** | | |  |  |  |  |  |  |  |  |
| **Subgroup** | **HR** | **Diff** | **SE** | **N** | **Variance** | **SEDiff** | **LLDiff** | **ULDiff** | **Zdiff** | **p (Zdiff)** |
| USA | 0.88 | 0.1 | 0.105 | 18 | 0.000613 | 0.050811 | -0.1829 | 0.382902 | 1.968088 | 0.488 |
| Rest of the World | 0.78 |  | 0.16 | 13 | 0.001969 |  |  |  |  |  |
| **Staging** | | | | | | | | | | |
| **Subgroup** | **HR** | **Diff** | **SE** | **N** | **Variance** | **SEDiff** | **LLDiff** | **ULDiff** | **Zdiff** | **p (Zdiff)** |
| Local | 0.7 | 0.3 | 0.069 | 13 | 0.000366 | 0.111652 | -0.16035 | 0.760354 | 2.686913 | 0.00714 |
| Regional | 1 |  | 0.22 | 4 | 0.0121 |  |  |  |  |  |
| **Subgroup** | **HR** | **Diff** | **SE** | **N** | **Variance** | **SEDiff** | **LLDiff** | **ULDiff** | **Zdiff** | **p (Zdiff)** |
| Local | 0.7 | 0.08 | 0.069 | 13 | 0.000366 | 0.0493 | -0.17617 | 0.336172 | 1.622709 | 0.105 |
| Locoregional | 0.78 |  | 0.17 | 14 | 0.002064 |  |  |  |  |  |
| **Subgroup** | **HR** | **Diff** | **SE** | **N** | **Variance** | **SEDiff** | **LLDiff** | **ULDiff** | **Zdiff** | **p (Zdiff)** |
| Regional | 1 | 0.22 | 0.22 | 4 | 0.0121 | 0.119014 | -0.28493 | 0.724933 | 1.848525 | 0.064 |
| Locoregional | 0.78 |  | 0.17 | 14 | 0.002064 |  |  |  |  |  |
| **Subgroup** | **HR** | **Diff** | **SE** | **N** | **Variance** | **SEDiff** | **LLDiff** | **ULDiff** | **Zdiff** | **p (Zdiff)** |
| Original | 0.76 | 0.18 | 0.079 | 22 | 0.000284 | 0.104697 | -0.40293 | 0.762928 | 1.719247 | 0.085 |
| Estimated | 0.58 |  | 0.31 | 9 | 0.010678 |  |  |  |  |  |
| **RR** | | | | | | | | | | |
| **HR calculation methodology** | | | | | | | | | | |
| **Subgroup** | **ES** | **Diff** | **SE** | **N** | **Variance** | **SEDiff** | **LCIDiff** | **UCI Diff** | **Zdiff** | **p (Zdiff)** |
| Original | 0.37 | 0.36 | 0.91 | 5 | 0.16562 | 0.414753 | -0.6636 | 0.812916 | 0.867987 | 0.38 |
| Estimated | 0.73 |  | 0.16 | 4 | 0.0064 |  |  |  |  |  |
| **DSS** | | | | | | | | | | |
| **Geographical location** | | | | | | | | | | |
| **Subgroup** | **ES** | **Diff** | **SE** | **N** | **Variance** | **SEDiff** | **LCIDiff** | **UCI Diff** | **Zdiff** | **p (Zdiff)** |
| USA | 1.16 | 0.79 | 0.22 | 9 | 0.005378 | 0.131879 | 0.262484 | 1.317516 | 5.990341 | <0.001 |
| Rest of World | 0.37 |  | 0.29 | 7 | 0.012014 |  |  |  |  |  |
| **HR calculation methodology** | | | | | | | | | | |
| **Subgroup** | **ES** | **Diff** | **SE** | **N** | **Variance** | **SEDiff** | **LCIDiff** | **UCI Diff** | **Zdiff** | **p (Zdiff)** |
| Original | 0.99 | 0.47 | 0.25 | 10 | 0.00625 | 0.174117 | -0.22647 | 1.166467 | 2.699337 | 0.00695 |
| Estimated | 0.52 |  | 0.38 | 6 | 0.024067 |  |  |  |  |  |
| **DFS** | | | | | | | | | | |
| **Geographical location** | | | | | | | | | | |
| **Subgroup** | **ES** | **Diff** | **SE** | **N** | **Variance** | **SEDiff** | **LCIDiff** | **UCI Diff** | **Zdiff** | **p (Zdiff)** |
| USA | 0.85 | 0.56 | 0.51 | 5 | 0.05202 | 0.228861 | -1.3799 | 2.499897 | 2.446905 | 0.0143 |
| Rest of World | 0.29 |  | 0.05 | 7 | 0.000357 |  |  |  |  |  |
| **HR Calculation methodology** | | | | | | | | | | |
| **Subgroup** | **ES** | **Diff** | **SE** | **N** | **Variance** | **SEDiff** | **LCIDiff** | **UCI Diff** | **Zdiff** | **p (Zdiff)** |
| Original | 0.45 | 0.12 | 0.13 | 8 | 0.002113 | 0.214971 | -0.29569 | 0.535692 | 0.558215 | 0.575 |
| Estimated | 0.33 |  | 0.42 | 4 | 0.0441 |  |  |  |  |  |

Notes: Diff: Magnitude of difference; SE: standard error; UL: Upper 95% limit; LL: lower 95% limit; PI: prediction intervals; CI: confidence intervals; HR: Hazard ratio

**Table 2:** Simple meta-regression results for changes in survival and recurrence outcomes.

| **Variable** | **HR** | **LCI-HR** | **UCI-HR** | **p-value** |
| --- | --- | --- | --- | --- |
| ***OS: SRx vs. SRx + RTx*** | | | | |
| *Length of outcome assessment (in years)* | 1 | 0.95 | 1.05 | 0.94 |
| *Number of Participants* | 1 | 1 | 1 | 0.41 |
| *Male proportion* | 0.01 | 0 | 2.11 | 0.09 |
| *SNLB* | 1.01 | 1 | 1.02 | 0.03 |
| *Stage I (N)* | 1 | 1 | 1 | 0.9 |
| *Stage II (N)* | 1 | 1 | 1 | 0.49 |
| *Stage III (N)* | 1 | 1 | 1 | 0.12 |
| *No. of patients with tumor in head and neck* | 1 | 1 | 1 | 0.99 |
| *No. of patients with tumor in all extremities* | 1 | 1 | 1 | 0.15 |
| *No. of patients with tumor in trunk* | 1 | 1 | 1 | < 0.001 |
| *Tumor depth (mm)* | 1.01 | 1 | 1.02 | < 0.001 |
| *Received WLE (Yes/No)* | 0.71 | 0.46 | 1.1 | 0.12 |
| *Received MMS (Yes/No)* | 1.16 | 0.87 | 1.55 | 0.29 |
| *No. who received SRx only* | 1 | 1 | 1 | 0.77 |
| *No. who received adjuvant RTx* | 1 | 1 | 1 | 0.84 |
| *Total RT dose (Gy)* | 1.57 | 1.14 | 2.17 | 0.01 |
| *Use of CTx (Yes/No)* | 1.08 | 0.67 | 1.76 | 0.74 |
| *No. who developed recurrence at follow-up* | 1 | 0.97 | 1.04 | 0.82 |
| ***OS: Any addition of CTx*** | | | | |
| *Use of CTx (Yes/No)* | 0.99 | 0.76 | 1.28 | 0.89 |
| *No. who received SRx only* | 1 | 0.99 | 1.01 | 0.43 |
| *No. who received adjuvant RTx* | 1 | 1 | 1 | 0.73 |
| ***LR*** | | | | |
| *Use of CTx (Yes/No)* | 0.44 | 0.1 | 1.89 | 0.19 |
| *No. who received adjuvant RTx* | 0.99 | 0.98 | 1 | 0.12 |
| ***RR*** | | | | |
| *Received WLE (Yes/No)* | 0.28 | 0.1 | 0.78 | 0.03 |
| *No. who received SRx only* | 0.99 | 0.98 | 1 | 0.08 |
| *No. who received adjuvant RTx* | 1.01 | 0.98 | 1.03 | 0.68 |
| ***DSS*** | | | | |
| *Length of outcome assessment (in years)* | 1.08 | 0.96 | 1.2 | 0.18 |
| *Number of Participants* | 1.01 | 1 | 1.01 | 0.03 |
| *Age* | 0.92 | 0.83 | 1.01 | 0.07 |
| *Male proportion* | 2.98 | 1.19 | 7.45 | 0.02 |
| *SNLB* | 1 | 1 | 1.01 | 0.08 |
| *No. of patients with tumor in head and neck* | 1.02 | 1 | 1.03 | 0.06 |
| *No. of patients with tumor in all extremities* | 1.01 | 1 | 1.02 | 0.04 |
| *Received WLE (Yes/No)* | 2.29 | 1.25 | 4.21 | 0.01 |
| *Received MMS (Yes/No)* | 0.29 | 0.2 | 0.44 | < 0.001 |
| *Received any type of excision* | 2.85 | 1.4 | 5.8 | 0.01 |
| *No. who received adjuvant RTx* | 1.01 | 1 | 1.02 | 0.01 |
| *Use of CTx (Yes/No)* | 2.29 | 1.06 | 4.98 | 0.04 |
| *Median follow-up (months* | 1.04 | 1 | 1.08 | 0.08 |
| *No who developed recurrence at follow-up* | 1.01 | 0.99 | 1.03 | 0.18 |
| ***DFS*** | | | | |
| *Length of outcome assessment (in years)* | 0.91 | 0.75 | 1.1 | 0.27 |
| *Number of Participants* | 1.01 | 1 | 1.02 | 0.16 |
| *Age* | 0.99 | 0.81 | 1.21 | 0.91 |
| *Male proportion* | 0.11 | 0.01 | 0.88 | 0.04 |
| *No. of patients with tumor in head and neck* | 1.02 | 0.99 | 1.04 | 0.23 |
| *No. of patients with tumor in all extremities* | 1.02 | 1 | 1.05 | 0.05 |
| *No. of patients with tumor in Trunk* | 1.09 | 0.99 | 1.19 | 0.08 |
| *Received WLE (Yes/No)* | 2.31 | 0.68 | 7.91 | 0.16 |
| *Received MMS (Yes/No)* | 1.27 | 0.78 | 2.08 | 0.29 |
| *Received any type of excision* | 2.02 | 1.01 | 4.07 | 0.05 |
| *No. who received adjuvant RTx* | 1.01 | 1 | 1.02 | 0.11 |
| *Use of CTx (Yes/No)* | 0.58 | 0.14 | 2.45 | 0.42 |
| *No who developed recurrence at follow-up* | 0.98 | 0.93 | 1.03 | 0.31 |

| **Table 3:** E- value for selected outcomes | | | | |
| --- | --- | --- | --- | --- |
| **Outcome** | **Control group** | **Intervention group** | **E-value** | **CI** |
| **OS** | SRx | SRx+RTx | 1.66 | 1.09 |
| **OS** | Any | CTx added | 1.85 | 1.58 |
| **LR** | SRx | SRx + RTx | 2.01 | 1 |
| **RR** | SRx | SRx + RTx | 3.09 | 1 |
| **DSS** | SRx | SRx + RTx | 1.26 | 1 |
| **DFS** | SRx | SRx + RTx | 3.52 | 1.28 |
